# Comprehensive Identification of RNA-Binding Domains in Human Cells

**DOI:** 10.1016/j.molcel.2016.06.029

**Published:** 2016-08-18

**Authors:** Alfredo Castello, Bernd Fischer, Christian K. Frese, Rastislav Horos, Anne-Marie Alleaume, Sophia Foehr, Tomaz Curk, Jeroen Krijgsveld, Matthias W. Hentze

**Affiliations:** 1 European Molecular Biology Laboratory (EMBL), Meyerhofstrasse 1, 69117 Heidelberg, Germany; 2 Department of Biochemistry, University of Oxford, South Parks Road, Oxford OX1 3QU, UK; 3 German Cancer Research Center (DKFZ), Im Neuenheimer Feld 280, 69120 Heidelberg, Germany; 4 Faculty of Computer and Information Science, University of Ljubljana, 1001 Ljubljana, Slovenia

## Abstract

Mammalian cells harbor more than a thousand RNA-binding proteins (RBPs), with half of these employing unknown modes of RNA binding. We developed RBDmap to determine the RNA-binding sites of native RBPs on a proteome-wide scale. We identified 1,174 binding sites within 529 HeLa cell RBPs, discovering numerous RNA-binding domains (RBDs). Catalytic centers or protein-protein interaction domains are in close relationship with RNA-binding sites, invoking possible effector roles of RNA in the control of protein function. Nearly half of the RNA-binding sites map to intrinsically disordered regions, uncovering unstructured domains as prevalent partners in protein-RNA interactions. RNA-binding sites represent hot spots for defined posttranslational modifications such as lysine acetylation and tyrosine phosphorylation, suggesting metabolic and signal-dependent regulation of RBP function. RBDs display a high degree of evolutionary conservation and incidence of Mendelian mutations, suggestive of important functional roles. RBDmap thus yields profound insights into native protein-RNA interactions in living cells.

## Highlights

• Experimental generation of an atlas of RNA-binding sites (RBS) in human cells
• RBS overlap with enzymatic cores and protein-protein interaction sites
• About half of the total RBS map to disordered protein regions
• RBS are enriched for phosphorylation, acetylation, and methylation sites

## Graphical Abstract

**Figure:**
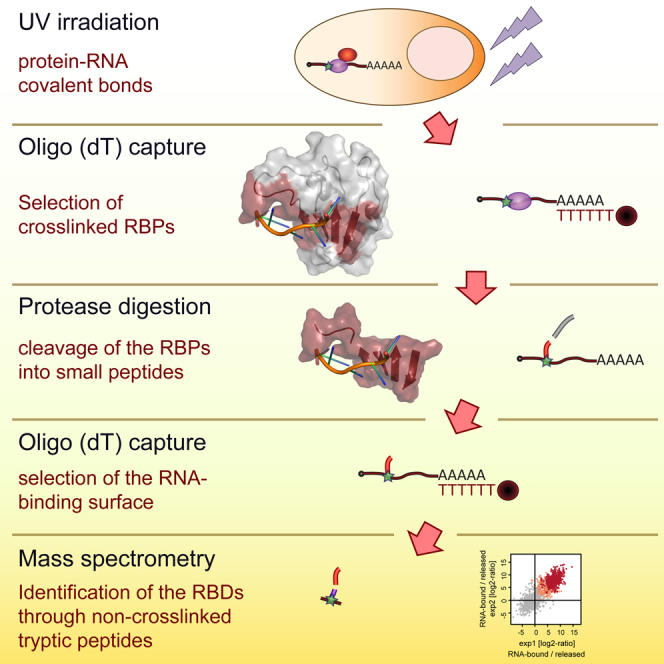

## Teaser

Many recently discovered RNA-binding proteins (RBPs) do not show architectural similarities with classical RBPs, and their modes of interaction with RNA were unclear. We developed and employed RBDmap as a method for the comprehensive determination of the RNA-interacting sites of RBPs, identifying more than a thousand such sites. These data yield unprecedented insight into RNA-protein interactions in cells with implications for numerous biological contexts.

## Introduction

RNA metabolism relies on the dynamic interplay of RNAs with RNA-binding proteins (RBPs) forming ribonucleoprotein complexes, which control RNA fate from synthesis to decay (Glisovic et al., 2008). Due to their central role in cell biology, it is unsurprising that mutations in RBPs underlie numerous hereditary diseases (Castello et al., 2013a, Lukong et al., 2008).

Many RBPs are modular, built from a limited pool of RNA-binding domains (RBDs), including the RNA recognition motif (RRM) and other canonical RBDs (Lunde et al., 2007). These domains have been characterized biochemically and structurally, furthering our understanding of protein-RNA interactions. The identification of unorthodox RBPs lacking canonical RBDs expands the scope of physiologically important protein-RNA interactions (e.g., Jia et al., 2008).

System-wide approaches to identify RBPs have recently been developed, including immobilization of RNA probes (Butter et al., 2009) or proteins (Scherrer et al., 2010, Tsvetanova et al., 2010), followed by in vitro selection of their interaction partners. These experiments identified numerous proteins previously unknown to bind RNA. While informative, in vitro protein-RNA interactions may arise non-physiologically from the electrostatic properties of RNA. To address this limitation, in vivo UV crosslinking has been used to covalently stabilize native protein-RNA interactions occurring in living cells. After cell lysis, proteins covalently bound to polyadenylated [poly(A)] RNAs are isolated by oligo(dT) selection and identified by quantitative mass spectrometry (Baltz et al., 2012, Castello et al., 2012). This approach (named RNA interactome capture) identified over a thousand RBPs in HeLa and HEK293 cells, hundreds of which were previously unknown to bind RNA. Subsequently, similar data sets were obtained from mouse embryonic stem cells, *Saccharomyces cerevisiae*, and *Caenorhabditis elegans* (Beckmann et al., 2015, Kwon et al., 2013, Matia-González et al., 2015, Mitchell et al., 2013), confirming earlier findings and further uncovering the repertoire of RBPs.

Several of the unorthodox RBPs identified in these studies have been characterized for their physiological roles in RNA biology. These include metabolic enzymes (Beckmann et al., 2015), regulators of alternative splicing (Papasaikas et al., 2015, Tejedor et al., 2015), the E3 ubiquitin ligase TRIM25 (Choudhury et al., 2014), or the FAST kinase domain-containing protein 2 (FASTKD2) (Popow et al., 2015). However, the RNA-binding regions of these unorthodox RBPs remain largely unknown.

To identify the interaction sites of such proteins with RNA, UV crosslinking followed by extensive RNase treatment has been used to detect the peptide mass shift induced by the crosslinked RNA remnant via mass spectrometry (Schmidt et al., 2012). While conceptually simple, the mass heterogeneity of the nucleotide remnant has rendered this approach challenging in practice. Some RBDs have been characterized in vitro using this approach (reviewed in Schmidt et al., 2012), and a sophisticated algorithm allowed assignment of 257 binding sites from 124 proteins in yeast (Kramer et al., 2014). While informative, this data set is strongly enriched for interactions mediated by RRMs, because the challenging identification of peptides with aberrant mass spectra requires both abundance and high crosslinking efficiency for detection. Nonetheless, 10% of the identified interaction sites mapped to non-canonical RBDs, supporting the existence of unanticipated modes of RNA binding.

Here, we develop and exploit RBDmap as a method for the in vivo identification of RBDs on a proteome-wide scale. We identified 1,174 high-confidence RNA-binding sites in 529 RBPs from HeLa cells, generating an unprecedented atlas of RNA-binding architectures in vivo.

## Results and Discussion

### Proteome-wide Mapping of RBDs by RBDmap

**Figure 1:**
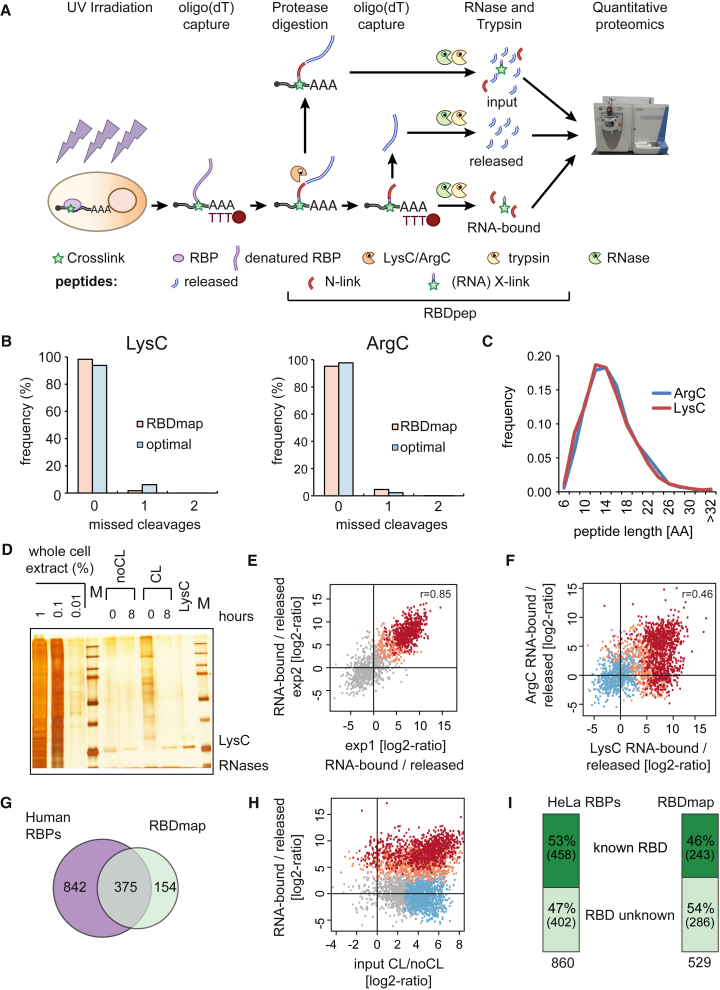
In Vivo Identification of RBDs by RBDmap (A) Schematic representation of the RBDmap workflow. (B) LysC- and ArgC-mediated proteolysis was monitored without trypsin treatment. The protease digestion under RBDmap conditions or in buffers typically used in MS studies (optimal) were compared to in silico digestions defining 0% miscleavage. The missed cleavages were calculated and plotted. (C) Distribution of MS-identified LysC/ArgC fragments based on their number of amino acids. (D) Silver staining shows the protein pattern of purified RBPs prior to and after LysC treatment (crosslinking: CL). (E) Scatter plot comparing the peptide intensity ratios between RNA-bound and released fractions. The peptides enriched in the RNA-bound fraction at 1% (RBDpep) and 10% FDR (candidate RBDpep) are shown in red and salmon, respectively (Pearson correlation coefficient: r). (F) Peptide intensity ratios between LysC and ArgC experiments computed from three biological replicates. The dots represent released peptides (blue), RBDpeps (red), candidate RBDpeps (salmon), and background peptides (gray). (G) Venn diagram comparing the proteins within the RBDmap data set and the HeLa, HEK293, and Huh-7 RNA interactomes. (H) Comparison of the peptide intensity ratios from three biological replicates between UV-irradiated and non-irradiated inputs (x axis) and between RNA-bound and released fractions (y axis) (color code as above). (I) Number of proteins harboring recognizable or unknown RBDs in the HeLa mRNA interactome (left) and in RBDmap dataset (right). See also Table S1 and Figure S1.

To define how RBPs bind to RNA in living cells, we extended RNA interactome capture (Castello et al., 2013b) by addition of an analytical protease digestion step followed by a second round of oligo(dT) capture and mass spectrometry (Figure 1A). First, UV light is applied to cell monolayers to covalently stabilize native protein-RNA interactions taking place at “zero” distance (Pashev et al., 1991). While UV exposure using dosages exceeding those used here can potentially promote protein-protein crosslinking (Davidenko et al., 2016, Suchanek et al., 2005), we could not detect such crosslinks under our conditions, evidenced by the lack of UV-dependent, high molecular weight complexes in RNase-treated samples (Figures S1A and S4A; Strein et al., 2014).

Proteins crosslinked to poly(A) RNA are isolated using oligo(dT) magnetic beads and purified by stringent washes that include 500 mM LiCl and chaotropic detergents (0.5% LiDS), efficiently removing non-covalent binders (Castello et al., 2012, Castello et al., 2013b). After elution, RBPs are proteolytically digested by either LysC or ArgC. These proteases were selected as best suited for RBDmap by an in silico simulation of their predicted cleavage patterns of known HeLa RBPs (Castello et al., 2012) and their compatibility with subsequent tryptic digestion (Figure S1B). Analysis by mass spectrometry (MS) of LysC- and ArgC-treated samples revealed an excellent match with the in silico predictions, as reflected by the low number of missed cleavages (Figures 1B and 1C). The extensive proteolysis of HeLa RBPs is achieved without compromising RNA integrity (Figures 1D and S1C–S1E). The average peptide length after LysC and ArgC treatment is ∼17 amino acids, which defines the resolution of RBDmap (Figure 1C). Note that the extensive protease treatment disrupts protein integrity, and thus protein-protein complexes that might have withstood the experimental conditions will be released into the supernatant.

We collected an input sample aliquot after UV irradiation, oligo(dT) selection, and protease digestion, which in principle should reflect the RNA interactome (Figure 1A). When compared to a non-irradiated specificity control, the resulting high-confidence RBPs overlap 82% with the previously published human RNA interactomes (Baltz et al., 2012, Beckmann et al., 2015, Castello et al., 2012). This high concordance shows that LysC and ArgC treatments are fully compatible with the RNA interactome capture protocol. The remaining two thirds of the LysC or ArgC-treated samples were subjected to a second round of oligo(dT) purification leading to two peptide pools (Figure 1A): (1) peptides released from the RNA into the supernatant, and (2) peptides remaining covalently bound to the RNA, representing the RNA-binding sites of the respective RBPs. Importantly, subsequent tryptic digestion of the RNA-bound LysC/ArgC fragments yields two classes of peptides: the portion that still remains crosslinked to the RNA (X-link) and its neighboring peptides (N-link) (Figure 1A). While the directly crosslinked peptides (X-link) are difficult to identify due to the heterogeneous mass shift induced by the residual nucleotides (Kramer et al., 2014, Schmidt et al., 2012), the native peptides adjacent to the crosslinking site (N-link) can be identified by standard MS and peptide search algorithms. The original RNA-bound region of the RBP (i.e., RBDpep; Figure 1A), which includes both the crosslinked peptide (X-link) and its unmodified neighboring peptides (N-link), is then re-derived in silico by extending the MS-identified peptides to the two nearest LysC or ArgC cleavage sites.

Analysis of the RNA-bound and released fractions by quantitative proteomics shows high correlation of the resulting peptide intensity ratios between independent biological replicates. These ratios follow a bimodal distribution with one mode representing the released peptides (gray) and the other the RNA-bound ones (red; Figures 1E and S1F). We detected 909 and 471 unique N-link peptides as significantly enriched in the RNA-bound fractions of LysC- or ArgC samples, respectively (1% false discovery rate, FDR) (Figure S1G). Notably, computed RNA-bound/released peptide intensity ratios also correlate between the LysC and ArgC data sets (Figure 1F), supporting the robustness of the workflow. Due to their different specificities, each protease also contributes unique 1% FDR RBDpeps to the complete peptide superset (Figure S1G), covering 529 RBPs that highly overlap with human RNA interactomes (Figure 1G) (Baltz et al., 2012, Beckmann et al., 2015, Castello et al., 2012). Proteins within the RBDmap data set range from low to high abundance (Figure S1H), following a similar distribution as the input fraction and the HeLa RNA interactome (Castello et al., 2012). Thus, RBDmap is not selective for highly abundant proteins. There were 154 additional RBPs that were identified here, helped by the reduction of sample complexity and of experimental noise by the additional proteolytic step and the second oligo(dT) capture. In agreement with this explanation, the relative abundance of corresponding RBDpeps is higher in the RNA-bound fractions than in the “input” samples (Figures 1H and S1I). Thus, RBDmap detects RNA-binding regions within hundreds of RBPs in one approach, even if it does not cover all RBPs identified by RNA interactome capture (Figure 1G). Proteins will be missed by RBDmap when (1) binding to non-polyadenylated RNAs, (2) displaying low crosslinking efficiency, (3) interacting with the phospho-sugar backbone, but not the nucleotide bases, or (4) lacking suitable cleavage sites for trypsin within the LysC and ArgC proteolytic fragments and hence lacking MS-identifiable N-link peptides. Thus, the distribution of arginines (R) and lysines (K) will influence whether a given RBP can be studied by RBDmap, and we used two different proteases to maximize the identification of RBDpeps.

About half of the RBPs covered by RBDpeps harbor well-established RBDs and play known functions in RNA biology, reflected by a strong and significant enrichment of RNA-related protein domains and biological processes comparable to the HeLa RNA interactome (Figures 1I and S1J). Note that the reduced RBP coverage of RBDmap compared to RNA interactome capture equally affects both well-established and unorthodox RBPs (Figures 1I and S1J).

### RBDmap “Rediscovers” Classic RBDs

**Figure 2:**
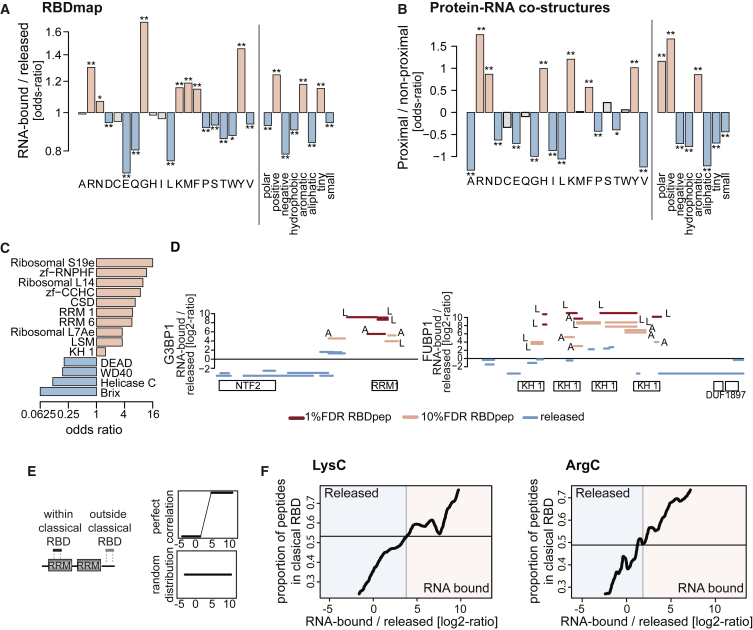
Identification of Well-Established RBDs by RBDmap (A) Amino acid enrichment within RBDpeps (salmon) over released (blue) proteolytic fragments (^∗^, 10% FDR and ^∗∗^, 1% FDR). (B) Amino acid enrichment within RNA-binding protein surfaces (≤4.3 Å to the RNA) over distant regions (>4.3 Å from the RNA) extracted from protein-RNA co-structures. (C) Bar plot showing the odds ratio of the most enriched known RBDs. (D) Distribution of RBDpeps and released fragments in a classical RBP. The x axis represents the protein sequence from N to C terminus, and the y axis shows the RNA-bound/released peptide intensity ratios. The protein domains are shown in boxes under the x axis (LysC: L and ArgC: A). (E) Schematic representation of RBDpeps mapping within or outside of classical RBDs (left). The idealized outcome of a perfect correlation between RBDpeps and classical RBDs (top right) and random distribution are shown (bottom right). (F) Computed ratio of peptides mapping within known RBDs versus outside RBDs, regarding their peptide RNA-bound to released ratios. The horizontal line represents the baseline for uncorrelated data (i.e., the proportion of peptides mapping to classical RBD in the whole validation set in absence of enrichment; see E bottom). See also Table S2 and Figure S2.

Interestingly, RNA-bound and released proteolytic fragments display distinct chemical properties. Released peptides are rich in negatively charged and aliphatic residues, which are generally underrepresented in RNA-binding protein surfaces (Figures 2A, 2B, and S2A). Conversely, RBDpeps are significantly enriched in amino acids typically involved in protein-RNA interactions, including positively charged and aromatic residues. These data show that the chemical properties of the RBDpeps resemble those expected of bona fide RNA-binding surfaces. As a notable exception, glycine (G) is enriched in RBDpeps, but depleted from protein-RNA interfaces derived from available structures (Figures 2A and 2B). Flexible glycine tracks can contribute to RNA binding via shape-complementarity interactions as described for RGG boxes (Phan et al., 2011). Hence, lack of glycine at binding sites of protein-RNA co-structures reflects the technical limitations of crystallographic studies regarding disordered protein segments.

Validating the RBDmap data, classical RBDs such as RRM, KH, cold shock domain (CSD), and Zinc finger CCHC, are strongly enriched in the RNA-bound fraction (Figure 2C). This enrichment can also be appreciated at the level of individual protein maps (Figures 2D and S2B–S2D). To evaluate the capacity of RBDmap to identify bona fide RBDs, we focused on RBPs that harbor at least one classical RBD (as listed in Lunde et al., 2007). MS-identified peptides from these proteins were classified as “within” or “outside” a classical RBD, according to their position within the proteins’ architecture (Figure 2E). The relative fraction of peptides within versus outside of the RBD was then plotted for each possible RNA-bound/released intensity ratio (Figure 2F). Correct re-identification of classical RBDs would lead to an ascending line (i.e., within/outside ratios should grow in parallel to the RNA-bound/release ratios; Figure 2E), while a random distribution of peptides within and outside of classical RBDs would yield a horizontal line (i.e., within/outside ratios do not vary in accordance with the RNA-bound/released ratios; Figure 2E). As shown in Figure 2F, the relative fraction of peptides mapping within classical RBDs increases in parallel with the RNA-bound/released ratios. Thus, RBDmap correctly assigns RNA-binding activity to well-established RBDs.

Unexpected initially, helicase domains are underrepresented in the RNA-bound fraction (Figure 2C). However, the high number of released helicase peptides likely reflects (1) the transitory and dynamic interactions that helicases establish with RNA, (2) the large protein segments of the domain situated far from the RNA, and (3) the predominance of interactions with the phospho-sugar backbone over nucleotide bases (Figures S2C–S2E) (Bono et al., 2006). Nevertheless, high-confidence RBDpeps are found at the exit of the helicase tunnel, as discussed below (Figures S2C–S2E).

### High-Resolution Determination of RNA-Binding Sites

**Figure 3:**
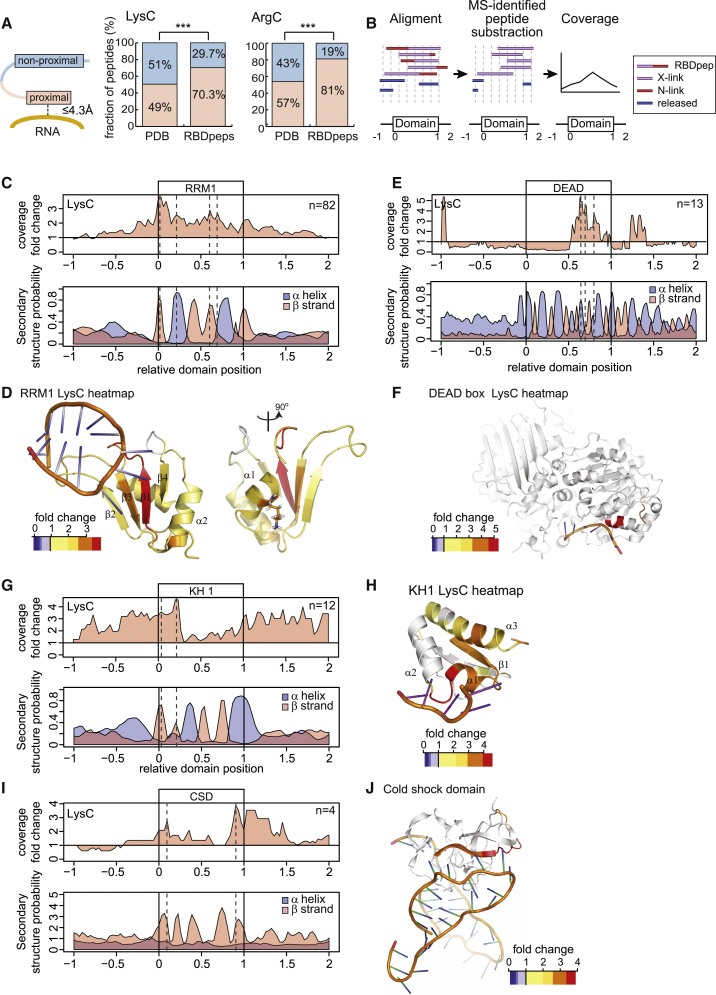
RBDmap Identifies RNA-Binding Regions with High Accuracy (A) Schematic representation of proximal and non-proximal peptides (left). The proteins within protein-RNA co-structures were digested in silico with LysC or ArgC and predicted fragments aligned with the RBDpep supersets. The left bars represent the proportion of proximal and non-proximal LysC/ArgC fragments in the complete structure superset (random probability). The right bars show the % of aligned RBDpeps that are RNA proximal or non-proximal (^∗∗∗^p < 0.001). (B) Schematic representation of the X-link peptide coverage analysis. (C) x axis represents the relative position of the RRM (from 0 to 1) and their upstream (−1 to 0) and downstream (1 to 2) regions. The ratio of the X-link over released peptides at each position of the RRM and surrounding regions using the LysC data set was plotted (top). The secondary structure prediction for each position of the RRM and flanking regions is shown (bottom). (D) The ratio of X-link over released peptides was plotted in a representative RRM-RNA structural model (PDB 2FY1) using a heatmap color code. (E) As in (C), but for the DEAD-box domain. (F) As in (D), but using the PDB 2J0S as a DEAD-box helicase model. (G) As in (C), but for the KH domain. (H) As in (D), but using the PDB 4B8T as a model for a KH domain. (I) As in (C), but for the CSD. (J) As in (D), but with the PDB 3TS2 as a model for a CSD. See also Table S2 and Figure S3.

For direct validation of the RBDmap data, we selected all those RBPs for which protein-RNA co-structures are available within the Protein Data Bank (PDB) repository. These were “digested” in silico with either LysC or ArgC, and the predicted proteolytic fragments were considered as “proximal” to RNA when the distance to the closest RNA molecule is 4.3 Å or less; otherwise, they were categorized as non-proximal (Figure 3A). About half of all LysC and ArgC fragments are proximal to RNA by this criterion, reflecting that many RBP structures are incomplete and focused on the RBDs (average protein coverage ∼50%). By contrast, 70.3% (LysC) and 81% (ArgC), respectively, of RBDpeps qualify as proximal, showing that RBPmap highly significantly enriches for peptides in close proximity to the RNA (Figure 3A). Several factors suggest that the pool of peptides classified as proximal in the analyzed structures even underestimates the performance of RBDmap: (1) in several structures of RBPs that harbor two or more RBDs, only one of the RBDs displays the interaction with RNA (e.g., PDB 3NNC) (Teplova et al., 2010). At least in some of these cases, structures lack RNA contacts of RBDs that likely occur in vivo. (2) Proteins are normally co-crystallized with short nucleic acids (5 to 8 nucleotides), and their physiological RNA partners likely establish additional interactions with the RBP. (3) RNA-protein co-structures usually reflect one interaction state, while protein-RNA interactions are typically more dynamic in vivo (Ozgur et al., 2015, Safaee et al., 2012).

RBDmap also correctly assigns RNA-binding regions within large protein complexes such as the nuclear cap-binding complex. The small nuclear cap-binding protein (NCBP) 2 (or CBP20) directly contacts mRNA via the cap structure (m7GpppG), while the larger NCBP1 (CBP80) interacts with NCBP2 (Mazza et al., 2002). In agreement, RBDmap defines the RNA-binding region of NCBP2 within the m7GpppG-binding pocket and no RBDpep is assigned to the large NCBP1 (Figure S3A). Moreover, RBDmap defines the corresponding RNA-binding sites within NCBP2 (Mazza et al., 2002) and its cytoplasmic counterpart eIF4E (Brown et al., 2007) (Figure S3B), in spite of their low sequence identity. The glutamyl-prolyl-tRNA synthetase (EPRS) represents a large non-canonical RBP that harbors two tRNA synthase domains separated by three WHEP motifs (Figures S3C and S3D). The first and second WHEP motif bind the GAIT RNA element present in the 3′ UTRs of a number of pro-inflammatory mRNAs (Jia et al., 2008), in complete agreement with the RBDmap data.

To test whether RNA-binding assignments of RBDmap can reach near single-amino acid resolution, we collected the complete set of RBDpeps and released peptides mapping to a given RBD class (e.g., RRM) and assessed their relative position within the domain (from 0 to 1) as well as its adjacent upstream (from −1 to 0) and downstream regions (from 1 to 2) (Figure 3B). The MS-identified part (N-link) of each RBDpep was then subtracted to infer the RNA-crosslinked (X-link) moiety(s), which cannot be identified by conventional MS due to their nucleotide remnant (Figures 1A and 3B). The X-link/released peptide ratio was calculated for each position in the domain, where high prevalence of X-link over released peptides will indicate RNA binding (Figure 3B). The high accuracy of this analysis is illustrated by the example profile obtained for RRMs. As shown in Figures 3C, 3D, and S3E, the highest X-link/released peptide ratio points to β strand 1, 2, and 3 as partners in the interaction with RNA, in agreement with the dozens of RNA-RRM co-structures available. Note that the LysC and ArgC proteases dissected the RRM in a differential manner: while LysC points to β strand 1 and 3, ArgC identifies β strand 2 as RNA-binding site, reflecting that the mapping capacity by these proteases depends on the distribution of lysines and arginines. Moreover, these data support the complementarity of the LysC and ArgC data sets to build accurate and comprehensive RNA-binding maps. Unexpectedly, we observed two discrete peaks of high X-link/released peptide ratio within the α helices placed at the back of the RRM. These peaks coincide with amino acids projected from the α helix to the RNA in several structures (Figure S3F) (Safaee et al., 2012, Teplova et al., 2010) and hence confirm the accuracy of RBDmap.

This analysis also successfully assigned correct RNA-binding sites to KH, DEAD-box helicase, and CSD, as shown in Figures 3E–3J, S3G, and S3H. The DEAD box helicase domain establishes interactions primarily with the phospho-sugar backbone of the RNA, while nucleotide bases project away from the protein core (Figure S3I). X-link peptide coverage of RBDmap for the DEAD box domain identifies one alpha helix in the helicase tunnel exit that coincides with the only position in RNA-protein co-crystals where multiple amino acids establish direct contacts with nucleotide bases. Interestingly, different binding orientations of the double-stranded RNA-binding motif (DSRM) have been observed in structural studies (Figure S3J) (Fu and Yuan, 2013, Ramos et al., 2000). The X-link peptide coverage analysis of the DSRM domain highlights the loop separating the second and third β strands as interaction partners with the double-stranded RNA (Figures S3J and S3K). Note that this loop is shown in several RNA-protein co-structures to be projected into the minor grove of the double-stranded RNA helix, establishing numerous interactions with the Watson-Crick paired bases (Lunde et al., 2007). In summary, RBDmap faithfully re-identifies the protein surfaces of canonical RBDs that contact nucleotide bases.

### Identification of Non-canonical RBDs

**Figure 4:**
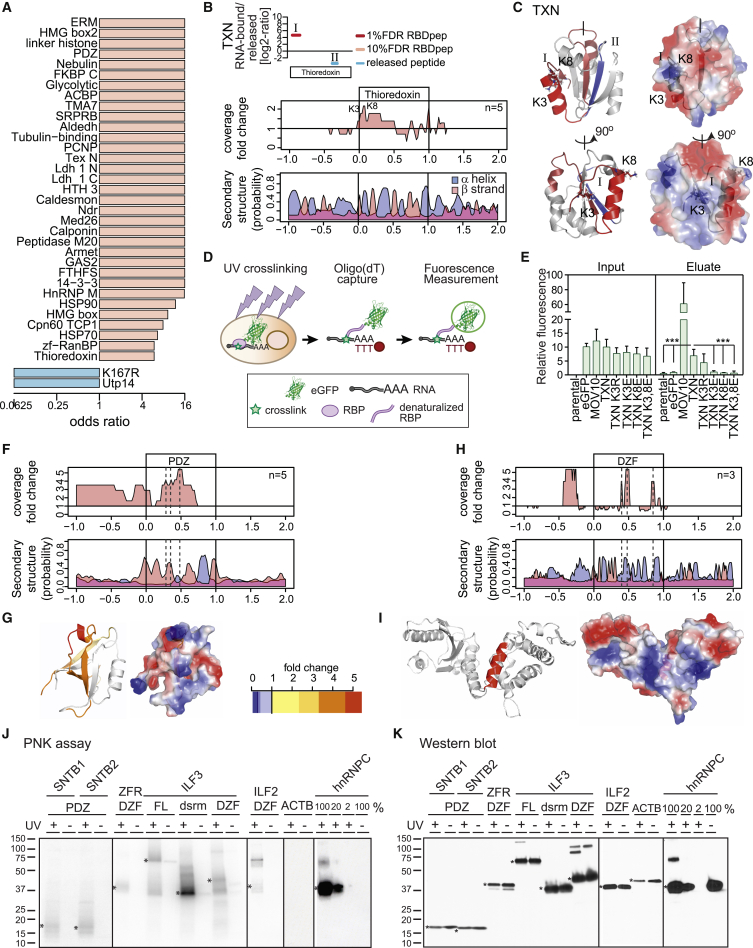
Globular RBDs Discovered by RBDmap (A) Odds ratios for the most highly enriched RBDs. (B) RBDpep and released peptides mapping to TXN as in Figure 2D (top). The ratio of the X-link over released peptide coverage at each position of the TXN fold as in Figure 3C is shown (middle). The secondary structure prediction for each position of the TXN fold and flanking regions is shown (bottom). (C) Crystal structure of human TXN (PDB 3M9J), K3 and K8 are highlighted, and the identified RBDpep is shown in red. (D) Schematic representation of the protocol for measurement of RNA-binding using eGFP fusion proteins. (E) Relative total (input) or RNA-bound (eluate) green fluorescence signal from cells expressing different eGFP fusion proteins (^∗∗∗^p < 0.01, t test, and n = 9). (F) As in (B), but for PDZ domain. (G) Ratio of X-link over released peptides plotted as a heatmap in a PDZ homology model. (H) As in (B), but for DZF domain. (I) As in (G), but using a DZF homology model. (J) Autoradiography of FLAG-HA tagged proteins after PNK assay. (K) Western blotting using an antibody against the HA tag. The polypeptides of the expected molecular masses are indicated by asterisks. See also Tables S2, S3, and S5 and Figure S4.

For more than half of the RBPs characterized by RBDmap, no functional or domain annotation related to RNA biology is currently available (Figures 1I and S1J). RBDpeps identify dozens of unorthodox globular RBDs associated with different molecular functions, including DNA binding, enzymatic cores, mediators of protein-protein interactions, or of protein localization (Figure 4A; Table S2). As an illustrative example, thioredoxin (TXN) catalyzes disulfide bond formation and has recently been discovered in RNA interactomes (Beckmann et al., 2015, Castello et al., 2012). RBDmap identifies an RBDpep at the N terminus of TXN (Figure 4B; Table S1) that overlaps with two solvent-exposed lysines (K3 and 8) highlighted as potential binding sites in the X-link coverage analysis for the TXN fold (Figures 4B and 4C). To evaluate this assignment functionally, we expressed TXN-eGFP fusion proteins in HeLa cells. Following in vivo UV crosslinking, oligo(dT) capture, and stringent washes, green fluorescence in eluates was measured to quantify RNA binding (Figure 4D) (Castello et al., 2013b, Strein et al., 2014). We used unfused eGFP as negative control and the well-established RNA-binding helicase MOV10 as a positive control for RNA binding (Gregersen et al., 2014). Although all the fusion proteins are expressed at similar levels in cells, only TXN-eGFP and MOV10-YFP co-purify with poly(A) RNAs significantly above background (Figure 4E). Mutation of K3 and/or K8 to glutamic acid (E) totally abrogates TXN RNA-binding activity. Conversely, conservative mutation to arginine (R) is tolerated. These results experimentally validate the accurate identification of a previously unknown RNA-binding region by RBDmap.

We also noticed clusters of RBDpeps within enzymes. Peptidyl prolyl *cis*/*trans* isomerases are classified based on their domain architecture into two groups: PPI and FKBP. This protein superfamily has close links to RNA metabolism, and two members, PPIE and PPIL4, harbor classical RRMs (Mesa et al., 2008). However, RNA interactome studies found 11 additional members of this family that lack RRMs as RBPs, suggesting the existence of a still unknown mechanism of RNA binding (Castello et al., 2012). RBDmap reveals this RNA-binding activity within both the PPI and FKBP folds (Tables S1 and S2). Although lacking sufficient peptide coverage to perform an X-link peptide analysis, we noticed two clusters of RBDpeps at the N- and C-termini of the FKBP fold that are located far apart in primary sequence, but close in 3D structure (Figures S4B and S4C). The mapped candidate RBD opposes the catalytic site.

Furthermore, we noticed clusters of RBDpeps in six chaperones of the heat shock protein (HSP) 90 and 70 families (Figure S4D). HSPs are induced by cellular stress and prevent protein misfolding and subsequent aggregation, which typically occur in disordered regions of RBPs in health and disease (Weber and Brangwynne, 2012). Indeed, HSPs have been functionally linked to RNA metabolism and translation (Iwasaki et al., 2010, Willmund et al., 2013). Chaperone domain binding to RNA may help to increase the local concentration of the chaperone machinery at ribonucleoprotein complexes to avoid the accumulation of pathological aggregates.

Apparently, numerous enzymes of intermediary metabolism bind RNA through regions in close proximity to their substrate-binding pockets. Specifically, the di-nucleotide binding domain (or Rossmann fold) and mono-nucleotide binding folds emerge as bona fide RBDs with 12 proteins mapped by RBDmap (Table S3), extending earlier observations (Cieśla, 2006, Nagy and Rigby, 1995). RBDpeps mapping to Aldolase (ALDO) A and C delimit the fructose 1,6 bisphosphate interacting domain (Figures S4E and S4F), suggesting that RNA and metabolite may compete for this binding pocket. Overall, the RBDpeps identified within metabolic enzymes show that the few well-characterized examples such as aconitase 1 (iron regulatory protein 1, IRP1), glyceraldehyde-3-phophate dehydrogenase, and thymidylate synthase may represent the tip of the iceberg of a more general engagement of metabolic enzymes with RNA (reviewed in Castello et al., 2015).

RBDmap also uncovers RNA-binding activities within PDZ, 14-3-3, ERM, and the tubulin-binding domains, which are involved in protein-protein interactions and protein localization (Figures 4F, 4G, and S4G–S4I). Due to the high peptide coverage of the PDZ domain, we could generate an X-link analysis (Figures 4F and 4G). This map shows a discrete RNA-binding site within a basic cavity formed by a short α helix and two β strands.

RBDmap also identifies RNA-binding sites within domains of unknown function such as NDR and DZF. N-myc downstream-regulated genes (NDRGs) represent a family of proteins with unknown function. NDRG1 is a metastasis suppressor relevant for cancer progression and prognosis (Chang et al., 2014), its exact molecular function has remained unknown. RBDmap resolves a conserved RNA-binding region within the NDR domain of NDRG1, NDRG2, and NDRG4. RBDpeps reproducibly map to the helix-loop-β strand structure at the C terminus of the NDR fold (Figures S4J and S4K). DZF is predicted to harbor nucleotidyltransferase activity (Kuchta et al., 2009) and to promote protein dimerization (Wolkowicz and Cook, 2012). The X-link peptide coverage analysis maps the RNA-binding region to a deep, basic cleft between two symmetrical domain subunits (Figures 4H and 4I). The RNA-binding activity of the DZF domain is compatible with its proposed nucleotidyltransferase function.

To independently assess RNA-binding of PDZ and DZF domains, we used the T4 polynucleotide kinase (PNK) assay as an orthogonal approach. In brief, cells are irradiated with UV light and, after lysis, RNA is trimmed with RNase I. Proteins of interest are immunoprecipitated under stringent conditions and the presence of RNA revealed by 5′ end phosphorylation with PNK and [γ-^32^P]-ATP, followed by SDS-PAGE and autoradiography. We generated Tet-inducible HeLa cell lines expressing the PDZ domain of β-1-syntrophin (SNTB) 1 and SNTB2, as well as the DZF domains of Zinc finger RNA-binding protein (ZFR) and interleukin enhancer-binding factor (ILF) 2 and ILF3, all fused to a FLAG-HA tag. As positive controls, we used the full-length ILF3 (FL), its DSRM domain alone, and hnRNPC, while actin (ACTB) was used as a negative control. The PNK assay shows radioactive bands of the expected molecular weight for all tagged PDZ and DFZ domains and only when UV light was applied to the cultured cells (Figures 4J and 4K). By contrast, no signal is detectable for the control ACTB. As expected, the DSRM domain of ILF3 also displays RNA-binding activity. Taken together, these data corroborate the RBDmap assignment of PDZ and DZF domains as RBDs.

Even if functional studies will have to define the physiological roles of these unconventional RBDs in the future, their biological relevance warrants consideration. It is possible that these RBDs may endow RBPs with “moonlighting” activities in posttranscriptional regulation, akin to cytosolic aconitase (IRP1) (Muckenthaler et al., 2008). Alternatively, the RBDs could serve as “docking sites” for regulatory or scaffolding RNAs that inhibit, activate, or modify protein functions. In analogy, innate immune effectors such as PKR, TLR3, TLR7, TLR8, or RIG-I, can be controlled by pathogen-derived RNAs (Barbalat et al., 2011, Yu and Levine, 2011). RNA may also serve to recruit proteins to RNPs, akin to NEAT1 RNA in paraspeckle formation (Clemson et al., 2009). The identification of these RBDs and the mapping of the RNA-interaction sites for hundreds of proteins serve as a critical step toward definition of the biological functions of these RBPs in detail.

### Disordered Regions Emerge as Frequent RNA Interaction Sites In Vivo

**Figure 5:**
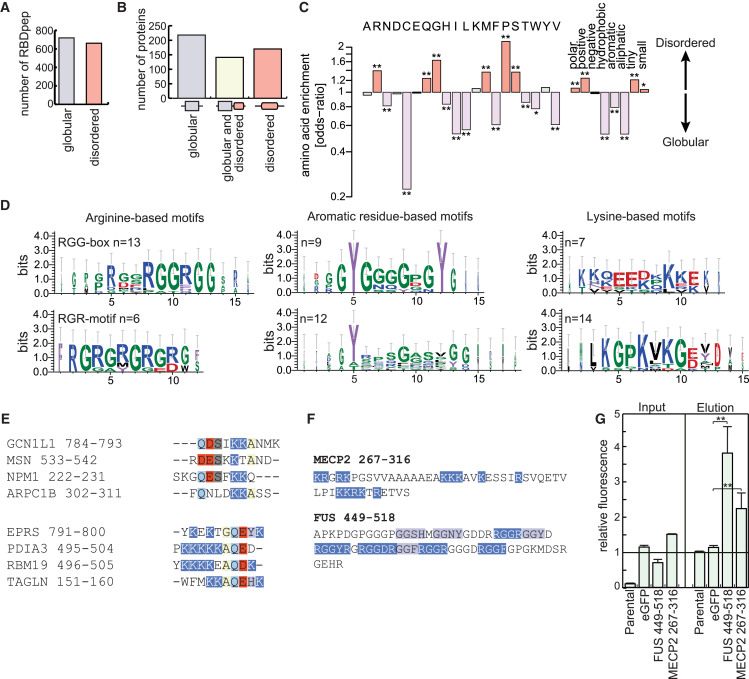
Disordered Protein Regions as RBDs (A) Number of RBDpeps mapping to globular and disordered domains. (B) Number of proteins mapped by at least one RBDpep solely in globular domains, in globular and disordered domains, or only in disordered motifs. (C) Amino acid enrichment between globular (violet) and disordered (pink) RBDs (^∗^, 10% FDR and ^∗∗^, 1% FDR). (D) Multiple sequence alignment of short, disordered RBDpeps with clustal omega. The sequence logos were extracted from aligned disordered fragments. (E) Examples of alignment of K-rich protein motifs. (F) Disordered RNA-binding motifs from FUS and MECP2 expressed as eGFP fusion. (G) Relative total (input) or RNA-bound (eluate) green fluorescence signal from cells expressing FUS_449–518_-eGFP, MECP2_267–316_-eGFP, or unfused eGFP as a negative control (^∗∗^p < 0.01, t test, and n = 6). See also Figure S5.

A high proportion of the human RBPs lack native 3D structure (Castello et al., 2012), and these disordered regions can occasionally engage in non-canonical protein-RNA interactions (45 examples reviewed in Järvelin et al., 2016). In some instances, these interactions can induce co-folding of both molecules (Phan et al., 2011). While this mode of interaction emerged recently, the scope of disordered motifs involved in RNA-binding remained unknown. Strikingly, half of the RBDpeps map to disordered regions, and RBDmap identifies a disordered RBD as the sole detectable RNA-binding site for 170 RBPs (Figures 5A 5B,, and S5A). Disordered RBDpeps largely mirror the chemical properties of the whole RBDpep superset, apart from the expected enrichment for disorder-promoting residues (proline [P], serine [S], and glycine [G]), as well as R and glutamine (Q) (Figures 5C and S5B).

Detailed analysis identifies clusters of disordered RBDpeps that can be classified on the basis of sequence motifs. While a few R-rich, RGG, and SR repeats have previously been shown to bind RNA experimentally (Järvelin et al., 2016), RBDmap expands the RNA-binding role of these motifs by dozens of additional examples (Figures 5D and S5C). The superset of RNA-binding RGG boxes can be subclassified by the lengths of the glycine linkers (Thandapani et al., 2013). Because glycines can position arginines and contribute to RNA binding providing shape complementarity, G-linker length could serve in setting the motif’s specificity for RNA. In agreement, both arginine and glycine substitutions impair RGG-RNA recognition (Phan et al., 2011).

Aromatic residues are typically found in hydrophobic cores. However, histidines (H), phenylalanines (F), and especially tyrosines (Y) occur within the RNA-binding disordered regions (Figures 5D and S5C). YGG repeats (also called [G/S]Y[G/S]) can promote protein aggregation in vitro, inducing hydrogel formation and amyloid-like fibers, as well as dynamic phase transitions in vivo (Han et al., 2012, Kato et al., 2012). Since YGG repeats are identified as a potential RNA-binding motif in our data set, it will be important to elucidate whether their RNA-binding capacity is affected by the aggregation state and, conversely, whether RNA-binding to such disordered linear motifs can affect phase transitions and granule formation (Zhang et al., 2015).

Lysine (K) combines with negatively charged residues, G, P, or Q, to form distinctive RNA-binding motifs (Figures 5D and S5C). The stoichiometry and distances between lysines and other amino acids are similar across analogous K-rich motifs present in non-homologous proteins (Figure 5E). Several copies of a repeat combining basic and acidic residues within the neuroblast differentiation-associated protein AHNAK are identified by RBDmap (Figure S5D), suggesting that low complexity regions can contribute to modular RNA-binding architectures, similar to globular RBDs. Interestingly, the K-rich regions within RBPs display similarities with the basic tails of DNA-binding proteins. The large capture radius of these disordered regions play important roles in transcription factor activity by favoring “hopping” and “sliding” over 3D diffusion to reach their target sequences (Vuzman et al., 2010). K-rich sequences may play similar roles in RBPs.

To validate the disordered regions identified by RBDmap as bona fide RNA-binding motifs, we fused the RGG-rich and the K-rich sequences from FUS and Methyl-CpG-binding protein 2 (MECP2), respectively, to eGFP and tested the fusion proteins with the same assay as in Figure 4D: both short motifs suffice to confer RNA-binding to eGFP (Figures 5F and 5G).

The biological function and mode of interaction of disordered regions with RNA should be further investigated.

### Uncovering Biological Properties of RBDs

**Figure 6:**
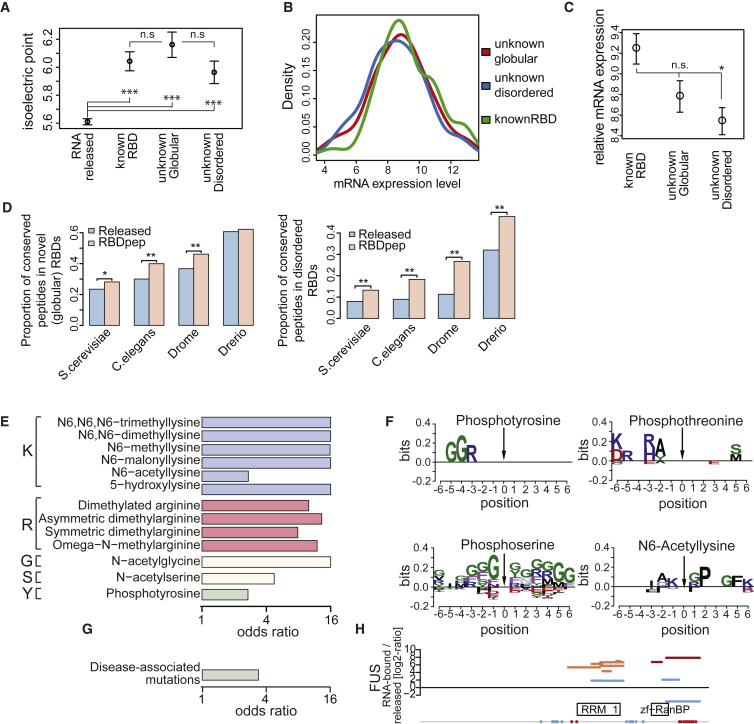
Features of Known and Previously Unknown RBDs (A) Dots show the mean isoelectric point of all LysC and ArgC fragments (the bars represent SEM) (^∗∗∗^p < 0.01 and not statistically significant: n.s.). (B) Density plot comparing mRNA abundances of known RBPs and previously unknown globular and disordered RBPs. (C) Dots show the mean of the mRNA abundance of the protein groups described in (B) (^∗^p < 0.05 and not statistically significant: n.s.). (D) Bar plot showing the conservation of RBDpeps and released fragments using *Homo sapiens* as reference (^∗^p < 0.05 and ^∗∗^p < 0.01). (E) Odds ratios for the most enriched PTMs in RBDpeps versus released fragments. (F) Sequence logos of conserved amino acids around posttranslational modifications. A position weight matrix is computed from all 12-mer sequences around the modified residue (10% FDR amino acids are shown). (G) Bar plot showing the odds ratio of Mendelian mutations occurring in RNA-bound over released fragments. (H) RBDmap of FUS. The position of the disease-associated mutations is represented as red or blue colored circles if mapping within or outside an RBDpep, respectively. See also Table S4.

Previously unknown RNA-binding globular and disordered regions display similar mean isoelectric points as known RBDs (Figure 6A), while their released counterparts exhibit a significantly lower isoelectric point, as expected. Thus, (1) both previously unknown and well-characterized RBDs share common chemical properties, (2) they differ from released fragments, and (3) the unorthodox RBDs do not artificially associate with RNA due to an abnormally high isoelectric point. Established RBPs and proteins harboring previously unknown globular and disordered RBDs display very similar mRNA abundance profiles, ranging from low to high levels, with a slight tendency to lower abundance for the unconventional folded and disordered RNA-binding regions (Figures 6B and 6C). Thus, proteins with unorthodox RBDs are not biased toward high abundance. Notably, RBDpeps in both globular and disordered RBDs are more highly conserved throughout evolution than their released counterparts (Figure 6D), suggesting functional relevance.

Cross-referencing of the RBDpep data sets with databases of curated posttranslational modifications shows that RNA-binding sites represent hot spots for defined post-translational modifications (PTMs, p = 2.025 × 10^−08^), including tyrosine phosphorylation, methylation, acetylation, and malonylation (Figure 6E). This finding suggests that, reminiscent of chromatin remodeling, RBDs are posttranslationally regulated and respond to signaling and metabolic cues. The conserved amino acid contexts of these PTMs implicate sequence-selective modifying enzymes (Figure 6F). Interestingly, acetylation frequently occurs in a lysine two positions upstream of a conserved proline (Figure 6F). Proline isomerization in the basic tail of histone H3 is regulated by acetylation of adjacent lysines and has notable consequences for protein conformation (Howe et al., 2014). Our results suggest the possibility that this regulatory mechanism could also apply to RBP regulation.

Our data also show that Mendelian disease mutations cluster within RBDs compared to natural variants (p = 0.0001796) (Figure 6G; Table S4). For example, one RBDpep maps to an RGG-box in FUS that is a hotspot for disease-associated mutations (Figure 6H) (Shang and Huang, 2016), and the RNA-binding activity of this region is validated here by an orthogonal approach (Figures 4D and 5G). Interestingly, a mutation in this region (R495X) causes amyotrophic lateral sclerosis (ALS) and correlates with impaired interaction of FUS with the SMN complex and reduced localization to nuclear gems (Yamazaki et al., 2012). The relationship between altered RNA-binding and disease phenotypes in this and other proteins deserves further exploration.

### Conclusions

RBDmap provides an unprecedented identification of RNA-binding regions of RBPs in living cells. It describes 1,174 high confidence (1% FDR) RNA-binding sites within 529 proteins. These sites have been validated as a whole by stringent statistical analyses (Figure 1) and cross-correlation with well-established RBPs and domains, previously studied by biochemical and structural means (Figures 2 and 3). We also validated a small number of previously unknown RBDs (TXN, PDZ, DZF, and the disordered regions of MECP2 and FUS) individually, applying orthogonal methods (Figures 4 and 5). Against this background, we recommend similar validation experiments for any individual RBD of interest before further in depth analyses.

Our data suggest that multifunctional globular domains, which combine RNA-binding with enzymatic functions or protein-protein interaction surfaces, are commonplace, not rare exceptions. These invoke additional functions for RNA, including the (allosteric or competitive) control of catalytic activities and of protein-protein interactions. Moreover, disordered regions are found to play common roles in native protein-RNA interactions, comprising half of the total RNA-binding sites identified.

The RNA-binding motifs identified here share physico-chemical features of well-established RBDs, are conserved across evolution, and represent hot spots for posttranslational modifications and disease-associated mutations. Individually and in combination, these features suggest important biological roles.

As a method, RBDmap can now be applied to other cell types and organisms such as *S. cerevisiae*, *Caenorhabditis elegans*, or *Drosophila melanogaster* to study the evolution of RBDs. It can also be applied to cells subjected to different experimental conditions to investigate the responses of RBPs to physiological cues such as e.g., stress, starvation, or differentiation.

## Experimental Procedures

### RBDmap

Initial UV crosslinking and oligo(dT) purification followed the mRNA interactome capture protocol (Castello et al., 2013b). Complete proteolytic digestions were performed with LysC or ArgC for 8 hr at 37°C. Polyadenylated RNA and crosslinked peptides were diluted in 20 mM Tris-HCl, 500 mM LiCl, 1 mM DTT, and 0.5 mM EDTA and recaptured on oligo(dT) beads. The supernatant was processed for MS (released peptides). oligo(dT) beads were washed as in Castello et al. (2013b). All fractions were treated with trypsin and labeled with stable isotopes in vitro (Boersema et al., 2008). Peptides were analyzed on a liquid chromatography-tandem MS (LC-MS/MS) platform. The R-scripts used for the analyses can be found in the R/Bioconductor data-package RBDmapHeLa (http://www.bioconductor.org). RBDmap data can be accessed under http://www-huber.embl.de/users/befische/RBDmap.

### MS, Protein Identification, and Quantification

Proteins were processed following standard protocols, and the resulting peptides were labeled with stable isotopes in vitro, fractionated, and analyzed on a nano-HPLC system (Proxeon) or nano-Acquity UPLC system (Waters) coupled directly to an LTQ Orbitrap Velos (Thermo Fisher Scientific).

### Data Analysis

A complete description of data analysis can be found in the Supplemental Information.

### Fluorescence-Based Method to Measure RNA-Binding In Vivo and PNK Assay

Tet-on HeLa cells expressing eGFP fusion proteins were generated as described elsewhere (Castello et al., 2012). Upon induction, cells were UV irradiated and subjected to small scale RNA interactome capture (Castello et al., 2013b). Eluates were measured in a plate reader. For PNK assays, cell monolayers were irradiated with 150 mJ/cm2 UV_254_ (Castello et al., 2013b). After cell lysis and RNase treatment, FLAG-HA tagged proteins were immunoprecipitated with an anti-FLAG antibody coupled to magnetic beads (M8823, Sigma Aldrich) and processed as in Beckmann et al. (2015). More detailed information can be found in the Supplemental Information.

## Author Contributions

A.C., B.F., and M.W.H. contributed to the conception and design of the project. A.C., R.H., and A.-M.A. carried out the experimental work. C.K.F., S.F., and J.K. performed the proteomic analyses. B.F., T.C., A.C., C.K.F., J.K., and M.W.H. performed the data analyses. A.C. and M.W.H. wrote the manuscript with input from all authors.

## Accession Numbers

The accession number for the proteomics data reported in this paper is ProteomeXchange Consortium (http://www.proteomexchange.org): PXD000883.

## Supplemental Information

Document S1. Supplemental Experimental Procedures, Figures S1–S5, and Tables S2, S3, and S5

Table S1. List of RBDs and Their Respective Peptides, Identified by RBDmap, Related to Figures 1 and S1

Table S4. Mendelian Mutations Occurring within the RNA-Bound Fragments of RBPs and Their Associated Diseases, Related to Figure 6

Document S2. Article plus Supplemental Information

## References

[bib1] Baltz A.G., Munschauer M., Schwanhäusser B., Vasile A., Murakawa Y., Schueler M., Youngs N., Penfold-Brown D., Drew K., Milek M. (2012). The mRNA-bound proteome and its global occupancy profile on protein-coding transcripts. Mol. Cell.

[bib2] Barbalat R., Ewald S.E., Mouchess M.L., Barton G.M. (2011). Nucleic acid recognition by the innate immune system. Annu. Rev. Immunol..

[bib3] Beckmann B.M., Horos R., Fischer B., Castello A., Eichelbaum K., Alleaume A.M., Schwarzl T., Curk T., Foehr S., Huber W. (2015). The RNA-binding proteomes from yeast to man harbour conserved enigmRBPs. Nat. Commun..

[bib4] Boersema P.J., Aye T.T., van Veen T.A., Heck A.J., Mohammed S. (2008). Triplex protein quantification based on stable isotope labeling by peptide dimethylation applied to cell and tissue lysates. Proteomics.

[bib5] Bono F., Ebert J., Lorentzen E., Conti E. (2006). The crystal structure of the exon junction complex reveals how it maintains a stable grip on mRNA. Cell.

[bib6] Brown C.J., McNae I., Fischer P.M., Walkinshaw M.D. (2007). Crystallographic and mass spectrometric characterisation of eIF4E with N7-alkylated cap derivatives. J. Mol. Biol..

[bib7] Butter F., Scheibe M., Mörl M., Mann M. (2009). Unbiased RNA-protein interaction screen by quantitative proteomics. Proc. Natl. Acad. Sci. USA.

[bib8] Castello A., Fischer B., Eichelbaum K., Horos R., Beckmann B.M., Strein C., Davey N.E., Humphreys D.T., Preiss T., Steinmetz L.M. (2012). Insights into RNA biology from an atlas of mammalian mRNA-binding proteins. Cell.

[bib9] Castello A., Fischer B., Hentze M.W., Preiss T. (2013). RNA-binding proteins in Mendelian disease. Trends Genet..

[bib10] Castello A., Horos R., Strein C., Fischer B., Eichelbaum K., Steinmetz L.M., Krijgsveld J., Hentze M.W. (2013). System-wide identification of RNA-binding proteins by interactome capture. Nat. Protoc..

[bib11] Castello A., Hentze M.W., Preiss T. (2015). Metabolic enzymes enjoying new partnerships as RNA-binding proteins. Trends Endocrinol. Metab..

[bib12] Chang X., Xu X., Ma J., Xue X., Li Z., Deng P., Zhang S., Zhi Y., Chen J., Dai D. (2014). NDRG1 expression is related to the progression and prognosis of gastric cancer patients through modulating proliferation, invasion and cell cycle of gastric cancer cells. Mol. Biol. Rep..

[bib13] Choudhury N.R., Nowak J.S., Zuo J., Rappsilber J., Spoel S.H., Michlewski G. (2014). Trim25 is an RNA-specific activator of Lin28a/TuT4-mediated uridylation. Cell Rep..

[bib14] Cieśla J. (2006). Metabolic enzymes that bind RNA: yet another level of cellular regulatory network?. Acta Biochim. Pol..

[bib15] Clemson C.M., Hutchinson J.N., Sara S.A., Ensminger A.W., Fox A.H., Chess A., Lawrence J.B. (2009). An architectural role for a nuclear noncoding RNA: NEAT1 RNA is essential for the structure of paraspeckles. Mol. Cell.

[bib16] Davidenko N., Bax D.V., Schuster C.F., Farndale R.W., Hamaia S.W., Best S.M., Cameron R.E. (2016). Optimisation of UV irradiation as a binding site conserving method for crosslinking collagen-based scaffolds. J. Mater. Sci. Mater. Med..

[bib17] Fu Q., Yuan Y.A. (2013). Structural insights into RISC assembly facilitated by dsRNA-binding domains of human RNA helicase A (DHX9). Nucleic Acids Res..

[bib18] Glisovic T., Bachorik J.L., Yong J., Dreyfuss G. (2008). RNA-binding proteins and post-transcriptional gene regulation. FEBS Lett..

[bib19] Gregersen L.H., Schueler M., Munschauer M., Mastrobuoni G., Chen W., Kempa S., Dieterich C., Landthaler M. (2014). MOV10 Is a 5′ to 3′ RNA helicase contributing to UPF1 mRNA target degradation by translocation along 3′ UTRs. Mol. Cell.

[bib20] Han T.W., Kato M., Xie S., Wu L.C., Mirzaei H., Pei J., Chen M., Xie Y., Allen J., Xiao G., McKnight S.L. (2012). Cell-free formation of RNA granules: bound RNAs identify features and components of cellular assemblies. Cell.

[bib21] Howe F.S., Boubriak I., Sale M.J., Nair A., Clynes D., Grijzenhout A., Murray S.C., Woloszczuk R., Mellor J. (2014). Lysine acetylation controls local protein conformation by influencing proline isomerization. Mol. Cell.

[bib22] Iwasaki S., Kobayashi M., Yoda M., Sakaguchi Y., Katsuma S., Suzuki T., Tomari Y. (2010). Hsc70/Hsp90 chaperone machinery mediates ATP-dependent RISC loading of small RNA duplexes. Mol. Cell.

[bib23] Järvelin A.I., Noerenberg M., Davis I., Castello A. (2016). The new (dis)order in RNA regulation. Cell Commun. Signal..

[bib24] Jia J., Arif A., Ray P.S., Fox P.L. (2008). WHEP domains direct noncanonical function of glutamyl-Prolyl tRNA synthetase in translational control of gene expression. Mol. Cell.

[bib25] Kato M., Han T.W., Xie S., Shi K., Du X., Wu L.C., Mirzaei H., Goldsmith E.J., Longgood J., Pei J. (2012). Cell-free formation of RNA granules: low complexity sequence domains form dynamic fibers within hydrogels. Cell.

[bib26] Kramer K., Sachsenberg T., Beckmann B.M., Qamar S., Boon K.L., Hentze M.W., Kohlbacher O., Urlaub H. (2014). Photo-cross-linking and high-resolution mass spectrometry for assignment of RNA-binding sites in RNA-binding proteins. Nat. Methods.

[bib27] Kuchta K., Knizewski L., Wyrwicz L.S., Rychlewski L., Ginalski K. (2009). Comprehensive classification of nucleotidyltransferase fold proteins: identification of novel families and their representatives in human. Nucleic Acids Res..

[bib28] Kwon S.C., Yi H., Eichelbaum K., Föhr S., Fischer B., You K.T., Castello A., Krijgsveld J., Hentze M.W., Kim V.N. (2013). The RNA-binding protein repertoire of embryonic stem cells. Nat. Struct. Mol. Biol..

[bib29] Lukong K.E., Chang K.W., Khandjian E.W., Richard S. (2008). RNA-binding proteins in human genetic disease. Trends Genet..

[bib30] Lunde B.M., Moore C., Varani G. (2007). RNA-binding proteins: modular design for efficient function. Nat. Rev. Mol. Cell Biol..

[bib31] Matia-González A.M., Laing E.E., Gerber A.P. (2015). Conserved mRNA-binding proteomes in eukaryotic organisms. Nat. Struct. Mol. Biol..

[bib32] Mazza C., Segref A., Mattaj I.W., Cusack S. (2002). Large-scale induced fit recognition of an m(7)GpppG cap analogue by the human nuclear cap-binding complex. EMBO J..

[bib33] Mesa A., Somarelli J.A., Herrera R.J. (2008). Spliceosomal immunophilins. FEBS Lett..

[bib34] Mitchell S.F., Jain S., She M., Parker R. (2013). Global analysis of yeast mRNPs. Nat. Struct. Mol. Biol..

[bib35] Muckenthaler M.U., Galy B., Hentze M.W. (2008). Systemic iron homeostasis and the iron-responsive element/iron-regulatory protein (IRE/IRP) regulatory network. Annu. Rev. Nutr..

[bib36] Nagy E., Rigby W.F. (1995). Glyceraldehyde-3-phosphate dehydrogenase selectively binds AU-rich RNA in the NAD(+)-binding region (Rossmann fold). J. Biol. Chem..

[bib37] Ozgur S., Buchwald G., Falk S., Chakrabarti S., Prabu J.R., Conti E. (2015). The conformational plasticity of eukaryotic RNA-dependent ATPases. FEBS J..

[bib38] Papasaikas P., Tejedor J.R., Vigevani L., Valcárcel J. (2015). Functional splicing network reveals extensive regulatory potential of the core spliceosomal machinery. Mol. Cell.

[bib39] Pashev I.G., Dimitrov S.I., Angelov D. (1991). Crosslinking proteins to nucleic acids by ultraviolet laser irradiation. Trends Biochem. Sci..

[bib40] Phan A.T., Kuryavyi V., Darnell J.C., Serganov A., Majumdar A., Ilin S., Raslin T., Polonskaia A., Chen C., Clain D. (2011). Structure-function studies of FMRP RGG peptide recognition of an RNA duplex-quadruplex junction. Nat. Struct. Mol. Biol..

[bib41] Popow J., Alleaume A.M., Curk T., Schwarzl T., Sauer S., Hentze M.W. (2015). FASTKD2 is an RNA-binding protein required for mitochondrial RNA processing and translation. RNA.

[bib42] Ramos A., Grünert S., Adams J., Micklem D.R., Proctor M.R., Freund S., Bycroft M., St Johnston D., Varani G. (2000). RNA recognition by a Staufen double-stranded RNA-binding domain. EMBO J..

[bib43] Safaee N., Kozlov G., Noronha A.M., Xie J., Wilds C.J., Gehring K. (2012). Interdomain allostery promotes assembly of the poly(A) mRNA complex with PABP and eIF4G. Mol. Cell.

[bib44] Scherrer T., Mittal N., Janga S.C., Gerber A.P. (2010). A screen for RNA-binding proteins in yeast indicates dual functions for many enzymes. PLoS ONE.

[bib45] Schmidt C., Kramer K., Urlaub H. (2012). Investigation of protein-RNA interactions by mass spectrometry--techniques and applications. J. Proteomics.

[bib46] Shang Y., Huang E.J. (2016). Mechanisms of FUS mutations in familial amyotrophic lateral sclerosis. Brain Res..

[bib47] Strein C., Alleaume A.M., Rothbauer U., Hentze M.W., Castello A. (2014). A versatile assay for RNA-binding proteins in living cells. RNA.

[bib48] Suchanek M., Radzikowska A., Thiele C. (2005). Photo-leucine and photo-methionine allow identification of protein-protein interactions in living cells. Nat. Methods.

[bib49] Tejedor J.R., Papasaikas P., Valcárcel J. (2015). Genome-wide identification of Fas/CD95 alternative splicing regulators reveals links with iron homeostasis. Mol. Cell.

[bib50] Teplova M., Song J., Gaw H.Y., Teplov A., Patel D.J. (2010). Structural insights into RNA recognition by the alternate-splicing regulator CUG-binding protein 1. Structure.

[bib51] Thandapani P., O’Connor T.R., Bailey T.L., Richard S. (2013). Defining the RGG/RG motif. Mol. Cell.

[bib52] Tsvetanova N.G., Klass D.M., Salzman J., Brown P.O. (2010). Proteome-wide search reveals unexpected RNA-binding proteins in *Saccharomyces cerevisiae*. PLoS One.

[bib53] Vuzman D., Azia A., Levy Y. (2010). Searching DNA via a “Monkey Bar” mechanism: the significance of disordered tails. J. Mol. Biol..

[bib54] Weber S.C., Brangwynne C.P. (2012). Getting RNA and protein in phase. Cell.

[bib55] Willmund F., del Alamo M., Pechmann S., Chen T., Albanèse V., Dammer E.B., Peng J., Frydman J. (2013). The cotranslational function of ribosome-associated Hsp70 in eukaryotic protein homeostasis. Cell.

[bib56] Wolkowicz U.M., Cook A.G. (2012). NF45 dimerizes with NF90, Zfr and SPNR via a conserved domain that has a nucleotidyltransferase fold. Nucleic Acids Res..

[bib57] Yamazaki T., Chen S., Yu Y., Yan B., Haertlein T.C., Carrasco M.A., Tapia J.C., Zhai B., Das R., Lalancette-Hebert M. (2012). FUS-SMN protein interactions link the motor neuron diseases ALS and SMA. Cell Rep..

[bib58] Yu M., Levine S.J. (2011). Toll-like receptor, RIG-I-like receptors and the NLRP3 inflammasome: key modulators of innate immune responses to double-stranded RNA viruses. Cytokine Growth Factor Rev..

[bib59] Zhang H., Elbaum-Garfinkle S., Langdon E.M., Taylor N., Occhipinti P., Bridges A.A., Brangwynne C.P., Gladfelter A.S. (2015). RNA controls polyQ protein phase transitions. Mol. Cell.

