# Supplementary material for: Comprehensive Identification of RNA-Binding Domains in Human Cells
Source: Mol Cell. 2016 Aug 18;63(4):696–710. doi: 10.1016/j.molcel.2016.06.029 (PMC5003815; doi:10.1016/j.molcel.2016.06.029)
Supplement: Document S1. Supplemental Experimental Procedures, Figures S1–S5, and Tables S2, S3, and S5 [file mmc1.pdf]

Molecular Cell, Volume *63*

Supplemental Information

Comprehensive Identification of RNA-Binding Domains in Human Cells

Alfredo Castello, Bernd Fischer, Christian K. Frese, Rastislav Horos, Anne-Marie Alleaume, Sophia Foehr, Tomaz Curk, Jeroen Krijgsveld, and Matthias W. Hentze

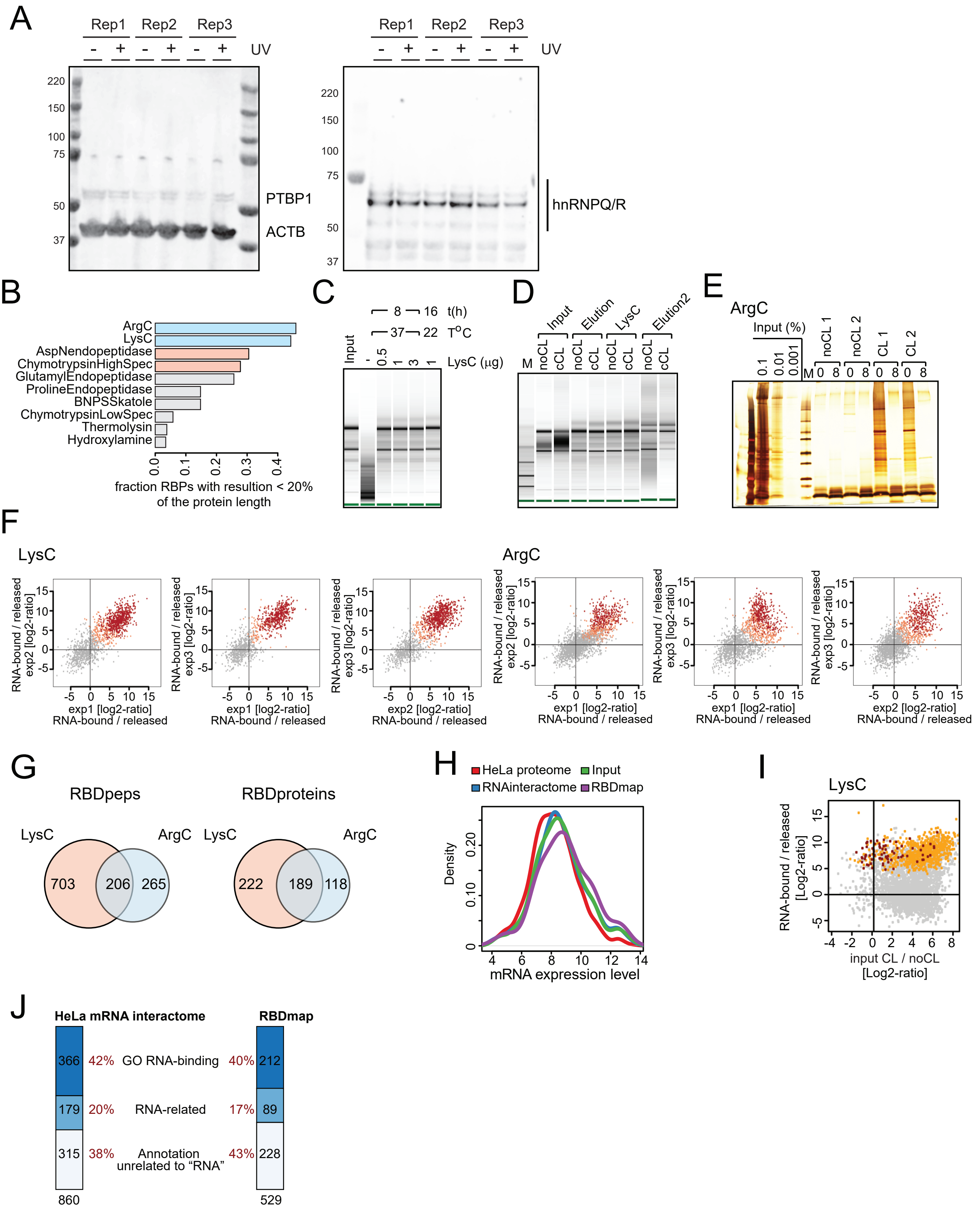
A
Rep1
Rep2
Rep3
UV
PTBP1
ACTB
hnRNPQ/R
B
ArgC
LysC
AspNendopeptidase
ChymotrypsinHighSpec
GlutamylEndopeptidase
ProlineEndopeptidase
BNPSSkatole
ChymotrypsinLowSpec
Thermolysin
Hydroxylamine
fraction RBPs with resultion < 20% of the protein length
C
Input
t(h)
T°C
LysC (µg)
D
M
Input
Elution
LysC
Elution2
noCL
cCL
E
ArgC
Input (%)
noCL 1
noCL 2
CL 1
CL 2
F
LysC
ArgC
RNA-bound / released exp2 [log2-ratio]
RNA-bound / released exp3 [log2-ratio]
exp1 [log2-ratio] RNA-bound / released
exp2 [log2-ratio] RNA-bound / released
G
RBDpeps
RBDproteins
LysC
ArgC
703
206
265
222
189
118
H
HeLa proteome
Input
RNAinteractome
RBDmap
Density
mRNA expression level
I
LysC
RNA-bound / released [Log2-ratio]
input CL / noCL [Log2-ratio]
J
HeLa mRNA interactome
RBDmap
366
42%
GO RNA-binding
40%
212
179
20%
RNA-related
17%
89
315
38%
Annotation unrelated to “RNA”
43%
228
860
529

Figure S1

Figure S1. Identification of RBDs by RBDmap. Related to Figure 1 and Table S1.
A) Western blot against ACTB, PTBP1 and hnRNPQ/R using whole cell lysates of UV_{254} irradiated and non-irradiated HeLa cells from three independent biological replicates. B) Computational simulation of protease efficiencies in RBDmap experiments. The RBPs of the HeLa mRNA interactome (Castello et al., 2012) were digested *in silico* using the different proteases available for MS experiments. The peptides identified in (Castello et al., 2012) were used as a proxy for protein coverage of an RBDmap experiment performed with the same cell line. We then selected the peptides that do not span the cleavage sites predicted for each protease and assumed the existence of the putative RNA-binding site at the centre of each RBP to calculate the best theoretical RBD resolution associated with each protease. The fractural number of proteins mapped for which the RBD was resolved to at least 20% of the actual protein length is represented. C) RNA integrity analysis under different LysC digestion conditions of oligo(dT)-purified samples (input). Samples were treated with proteinase K and monitored by bioanalyser. D) RNA analysis using bioanalyser of a representative LysC RBDmap experiment. E) Protein quality control of two independent experiments using ArgC. Poly(A) RNA extracted from UV irradiated (CL) and non-irradiated (noCL) cells was purified by oligo(dT) selection. Co-purified proteins were treated with 1µg of ArgC and analysed by silver staining prior to and after protease digestion. Optimization of LysC digestion of UV-irradiated oligo(dT) purified samples (input) applying different protease concentrations, incubation times and temperatures. F) Scatter plots comparing the peptide intensity ratios between RNA-bound and released fractions of three independent LysC and ArgC experiments. The peptides enriched in the RNA-bound over the released fraction at 1% and 10% FDR, respectively, are shown in red and salmon. G) Venn diagram comparing LysC and ArgC datasets at the peptide or protein level at 1% FDR. H) Density of mRNA levels of the whole HeLa proteome (red), the HeLa RNA interactome (Castello et al., 2012) (blue), the input sample (i.e. equivalent to the HeLa mRNA interactome - green), and proteins assigned with at least one 1% FDR RNA-binding site by RBDmap (purple). I) Scatter plot comparing the average peptide intensity ratios from three biological replicates between UV irradiated and non-irradiated samples (X axis) and between RNA-bound and released fractions (Y axis). Red represents RBDpeps (1% FDR) belonging to newly discovered proteins, while yellow peptides represent the rest of RBDpeps. J) Number of proteins annotated with the GO term RNA-binding, with a GO term related to RNA, or with an annotation unrelated to RNA in the HeLa mRNA interactome (left) and in RBDmap datasets (right).

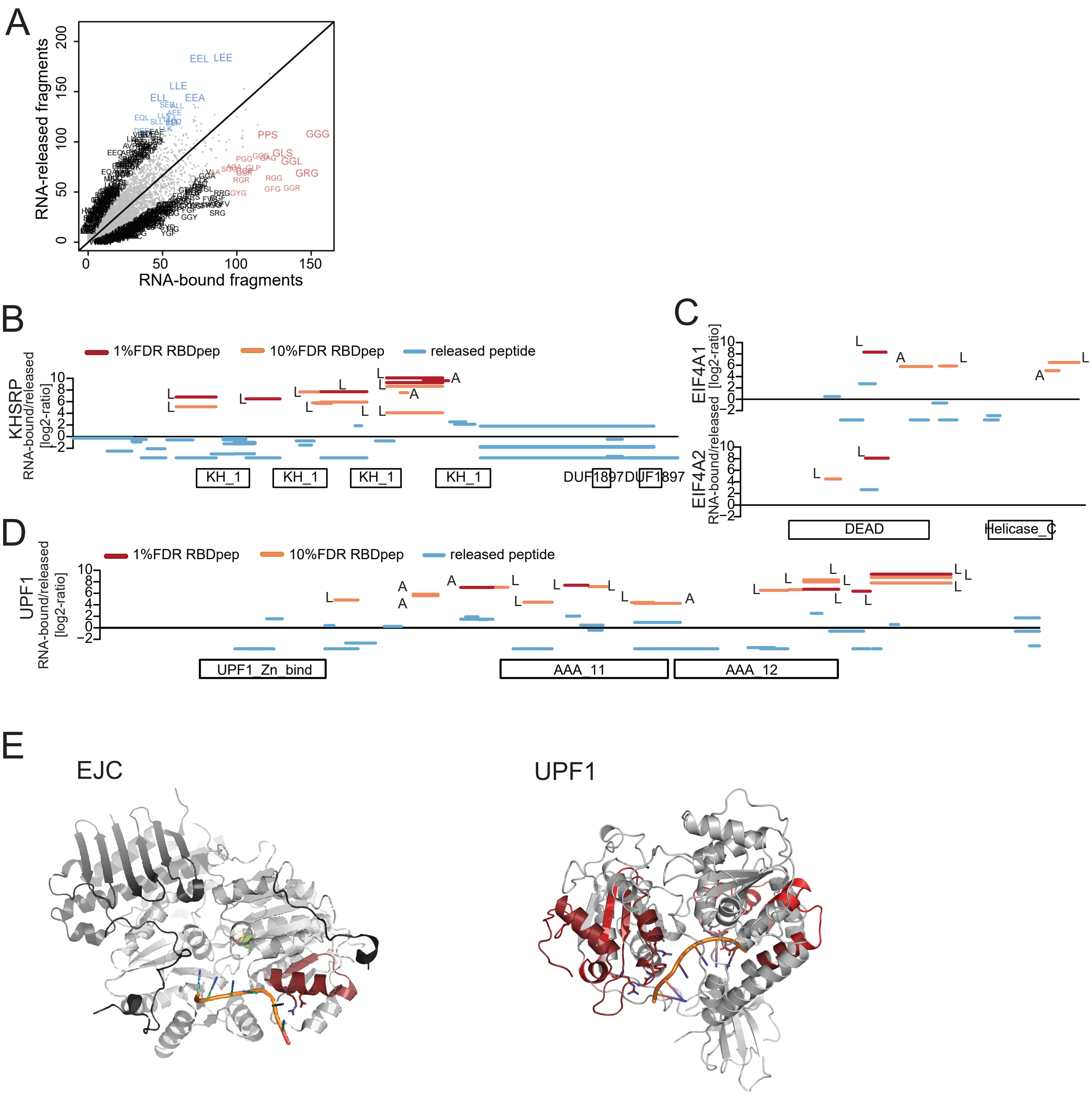

Figure S2. Benchmarking RBDmap. Related to Figure 2 and Table S2.
A) Enrichment of peptide trimers in RNA-bound (X axis) and released (Y axis) proteolytic fragments. In salmon and blue are the most abundant trimers in RNA-bound or released fractions. B-D) LysC and ArgC proteolytic fragment distribution of an illustrative KH-domain (B), DEAD box- (C) or AAA_11/AAA_12- (D) containing RBP. X axes represent proteins from N- to C-termini, while the Y axes show the RNA-bound/released peptide intensity ratios. Positions of the protein domains are shown in boxes under the X axis. E) The RBDpep (red) conserved between EIF4A1 and EIF4A2 was placed in the structure of their homolog EIF4A3 (light grey), which was crystalized in a complex with MAGOH, Y14 and barentz (dark grey) forming the exon junction complex (EJC, PDB 2j0s) (Bono et al., 2006). This region is highly conserved between the three homologs (EIF4A3 LDYGQ-HVVAGTPGRVFD-MIRRRSLRTR; EIF4A1, LQMEAPHIIVGTPGRVFDMLNRRYLSPK EIF4A2 LQAEAPHIVVGTPGRVFDMLNRRYLSPK) and is placed at the exit of the RNA tunnel (left panel). Right panel shows the RBDpeps (red) within UPF1, projected in the crystal structure of UPF1 with RNA (PDB 2xzo) (Chakrabarti et al., 2011).

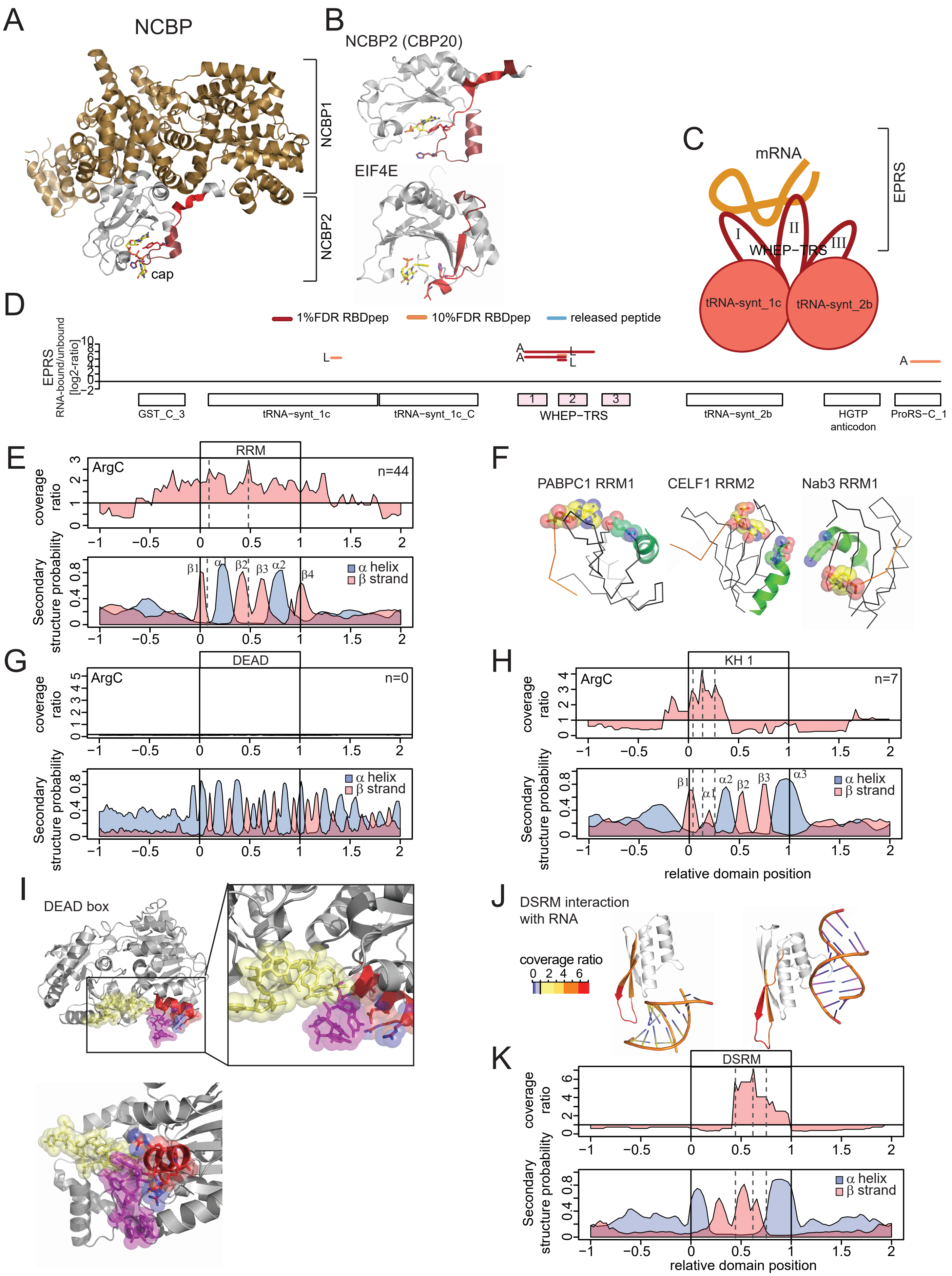

A
NCBP
NCBP1
NCBP2
cap
B
NCBP2 (CBP20)
EIF4E
C
mRNA
EPRS
I
II
III
WHEP-TRS
tRNA-synt_1c
tRNA-synt_2b
D
1%FDR RBDpep
10%FDR RBDpep
released peptide
EPRS
RNA-bound/unbound
[log2-ratio]
GST_C_3
tRNA-synt_1c
tRNA-synt_1c_C
WHEP-TRS
tRNA-synt_2b
HGTP anticodon
ProRS-C_1
E
RRM
ArgC
n=44
coverage ratio
Secondary structure probability
α helix
β strand
F
PABPC1 RRM1
CELF1 RRM2
Nab3 RRM1
G
DEAD
ArgC
n=0
H
KH 1
ArgC
n=7
relative domain position
I
DEAD box
J
DSRM interaction with RNA
coverage ratio
K
DSRM
relative domain position

Figure S3

Figure S3. RBDmap identifies well-established RNA-binding surfaces in known RBPs with high accuracy. Related to Figure 3 and S2.

A) Crystal structure of the nuclear cap-binding complex bound to the cap structure (PDB 1h2t) (Mazza et al., 2001). NCBP2 is depicted in grey and NCBP1 in gold. RBDpeps are shown in red. B) Location of the RBDpep in NCBP2 (PDB 1h2t) (Mazza et al., 2001) and its cytoplasmic homolog EIF4E (PDB 2v8x). C) Schematic representation of the reported interaction mechanism of EPRS with mRNAv (Jia et al., 2008). D) The RBDpep distribution of the EPRS protein matches the biochemical and functional data reported in (Arif et al., 2009; Jia et al., 2008; Mukhopadhyay et al., 2008). E) X axis represents the relative position of the RRM (from 0 to 1) and their upstream (-1 to 0) and downstream (1 to 2) regions. The ratio of the X-link over released peptides at each position of the RRM and surrounding regions using the ArgC dataset was computed and plotted (top). Secondary structure prediction for each position of the RRM and flanking regions (bottom). F) Crystal structures showing the interaction of amino acids in the α-helices of the RRM with the RNA (PDBs 4f02, 3nnc, 2l41). These structures agree with the LysC X-link coverage analysis in Figure 3C. G) As in (E) but for DEAD box domain. H) As in (E) but for KH1. I) Detail of eIF4A3 (DEAD-box) interacting with RNA (PDB 2j0s). RNA is shown in pale yellow, except for the ribonucleotides that are contacted by amino acids projected from the DEAD-box domain, which are shown in magenta. The protein region enriched in the X-link peptide coverage analysis is shown in red. J) The ratio of X-link over released peptides was plotted for two structures in which the DSRM domain is bound to double stranded RNA in different orientations (PDBs 3vyx, 3adl) using a heat map color code. K) As in (E) but for DSRM.

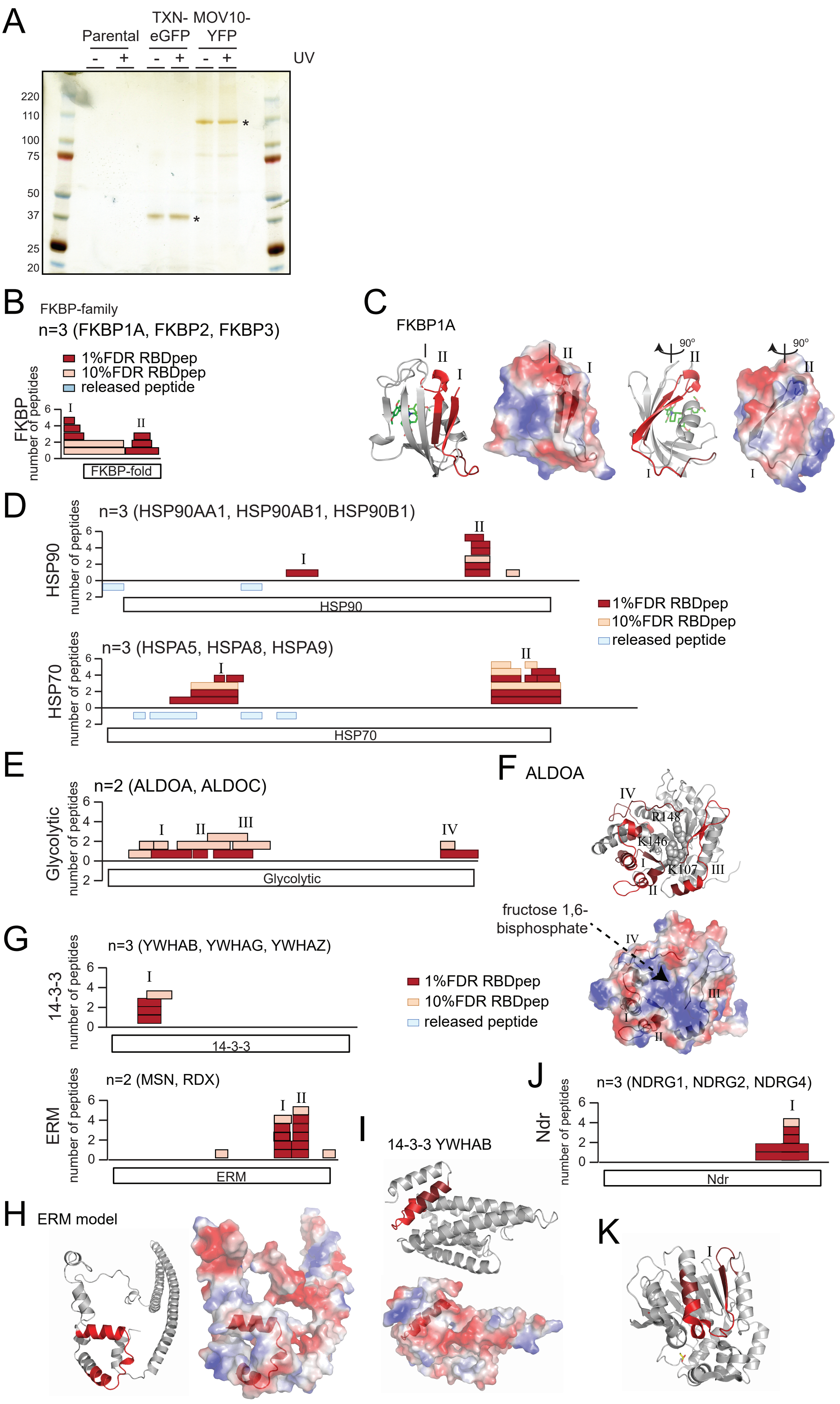
A
Parental
TXN-eGFP
MOV10-YFP
UV
220
110
100
75
50
37
25
20
B FKBP-family
n=3 (FKBP1A, FKBP2, FKBP3)
1%FDR RBDpep
10%FDR RBDpep
released peptide
FKBP
number of peptides
FKBP-fold
C FKBP1A
D
n=3 (HSP90AA1, HSP90AB1, HSP90B1)
HSP90
n=3 (HSPA5, HSPA8, HSPA9)
HSP70
E
n=2 (ALDOA, ALDOC)
Glycolytic
F ALDOA
R148
K146
K107
fructose 1,6-bisphosphate
G
n=3 (YWHAB, YWHAG, YWHAZ)
14-3-3
n=2 (MSN, RDX)
ERM
H ERM model
I 14-3-3 YWHAB
J
n=3 (NDRG1, NDRG2, NDRG4)
Ndr
K

Figure S4

Figure S4. Novel globular RBDs. Related to Figure 4, Table S2 and S3.

A) HeLa Flip-In Trex (parental), TXN-eGFP and MOV10-YFP were induced overnight with tetracycline. Cells were UV-irradiated or with 254 nm UV light or left untreated. Lysates from these cells were used for immunoprecipitation of GFP/YFP fusion proteins with GFP_Trap_A, and eluates were analyzed by silver staining. B) RBDpep distribution across all the FKBP protein family members characterized by RBDmap (FKBP1A, FKBP2, FKBP3). C) Crystal structure of FKBP1 bound to a synthetic ligand (PDB 1bl4). The electrostatic potential of the protein surface is shown in blue for basic and red for acidic surfaces. D) As in (B) but for HSP90 (top) and HSP70 (bottom) protein family members. E) As in (B), but for aldolase A and C. F) Ribbon diagram of ALDOA (top), where amino acids involved in the interaction with fructose 1,6 bisphosphate are shown as spheres (PDB 2ld). RBDpeps are shown in red. The electrostatic potential of the protein surface is shown in the bottom panel (blue, basic; red, acidic). G) As in (B) but for 14-3-3 and ERM protein families. H, I and K) Ribbon diagrams and the electrostatic potential of ERM (H), 14-3-3 (I) and Ndr (K) using homology models generated with Phyre2 (Kelley and Sternberg, 2009). J) As (B) but for NDRG protein family.

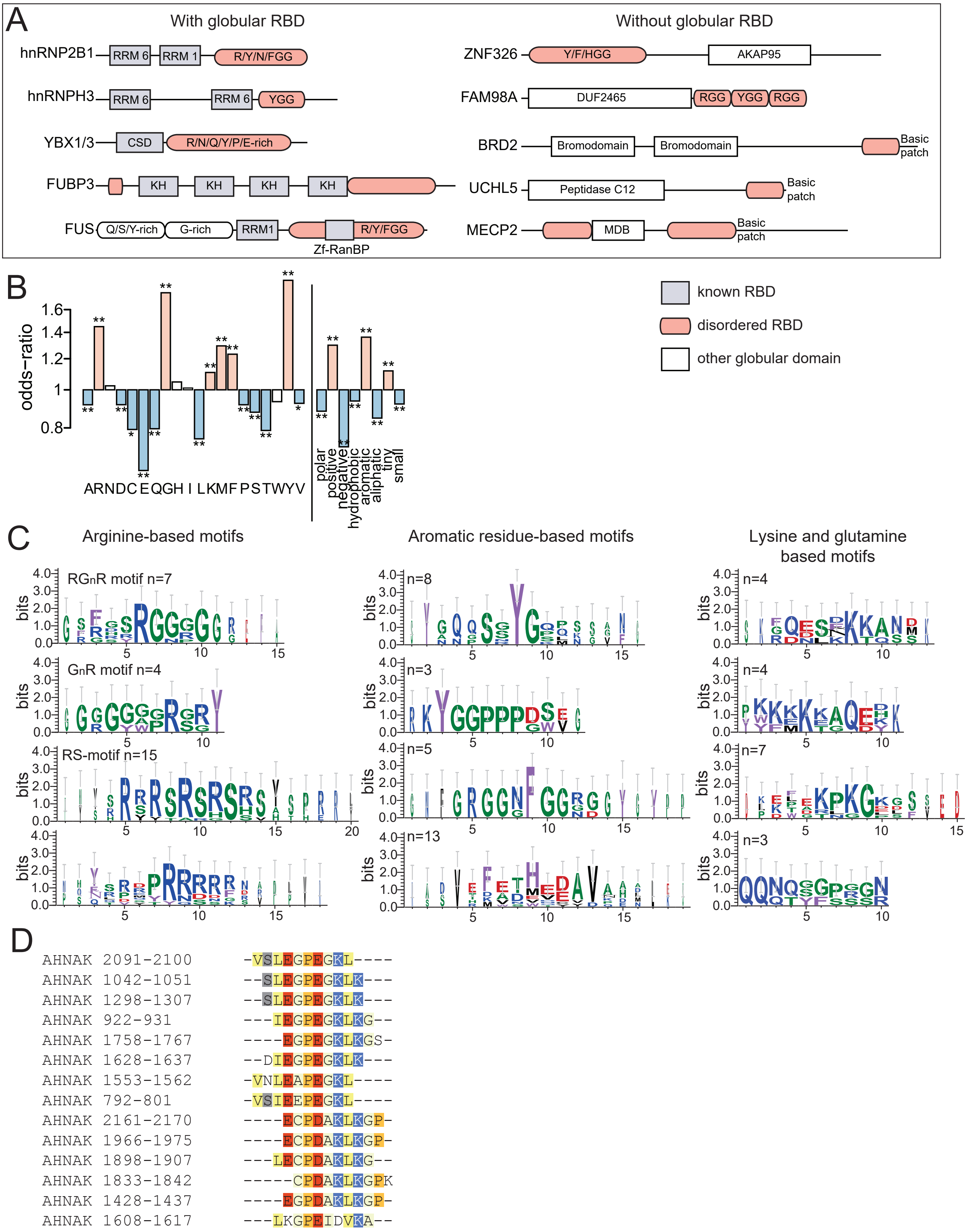

Figure S5. Disordered RNA-binding domains. Related to Figure 5

A) Schematic representation of the protein architecture of proteins harboring RNA-binding globular domains (violet) or/and disordered domains (pink). B) Amino acid enrichment within disordered RNA-bound over released proteolytic fragments mapping to disordered domains; *, 10% FDR; **, 1% FDR. C) Sequence logos extracted from aligned disordered motifs for R-based motifs, aromatic residue-based motifs and K/Q-based motifs. D) Complex pattern (VSLEGPEGKLKGP) found in multiple RBDpeps across AHNAK protein.

Pfam.name	p.value	odds.ratio	p.adj	boundPep	releasedPep
RRM_1	1.52E-82	5.953171222	1.69E-79	310	252
Pfam-B_2662	3.76E-30	4.490952613	2.10E-27	134	127
RRM_6	3.01E-20	5.749713541	1.12E-17	70	50
Pfam-B_1366	8.51E-20	6.740684806	2.37E-17	61	37
Pfam-B_4694	5.77E-13	3.724269703	1.08E-10	64	70
Pfam-B_14250	5.81E-13	9.182760264	1.08E-10	32	14
Pfam-B_7552	3.89E-11	5.513247616	6.20E-09	37	27
Pfam-B_11139	4.95E-11	16	6.91E-09	19	3
Pfam-B_6593	1.86E-10	16	2.31E-08	14	0
Pfam-B_2256	3.86E-10	3.80115872	4.31E-08	48	51
Pfam-B_2745	1.90E-09	7.990690535	1.93E-07	24	12
Pfam-B_659	2.13E-09	3.16435457	1.98E-07	54	69
DEAD	7.61E-09	0.203219496	6.53E-07	9	170
Pfam-B_12180	3.05E-08	6.556799279	2.27E-06	23	14
Pfam-B_15812	3.05E-08	6.556799279	2.27E-06	23	14
Pfam-B_1591	3.93E-08	5.140209469	2.74E-06	27	21
Pfam-B_19749	4.87E-08	16	3.20E-06	12	1
Pfam-B_19654	3.26E-07	6.877186121	2.02E-05	19	11
CSD	1.57E-06	6.759939713	9.20E-05	17	10
Pfam-B_1402	4.61E-06	16	0.000244857	9	1
ERM	4.61E-06	16	0.000244857	9	1
Pfam-B_6773	5.00E-06	16	0.000253519	10	2
Pfam-B_7751	1.02E-05	9.516635177	0.000495781	12	5
HMG_box_2	1.38E-05	16	0.000617555	7	0
Ribosomal_S19e	1.38E-05	16	0.000617555	7	0
Pfam-B_10135	1.46E-05	4.445449893	0.00062774	19	17
K167R	3.35E-05	0.0625	0.001382788	0	48
zf-CCHC	3.61E-05	8.717395407	0.001439234	11	5
Pfam-B_17918	3.80E-05	3.076407469	0.001463008	27	35
Pfam-B_2594	5.07E-05	9.900453408	0.001885413	10	4
Helicase_C	5.53E-05	0.116237005	0.001990639	2	67
zf-RNPHF	6.80E-05	11.86996167	0.002372407	9	3
Pfam-B_19575	0.000183073	3.312635041	0.006191206	20	24
HSP70	0.000271583	6.598431054	0.008914314	10	6
Pfam-B_5861	0.000337941	13.8327341	0.009014141	7	2
Pfam-B_16169	0.000337941	13.8327341	0.009014141	7	2
Pfam-B_18189	0.000339242	16	0.009014141	5	0
FKBP_C	0.000339242	16	0.009014141	5	0
Nebulin	0.000339242	16	0.009014141	5	0
PDZ	0.000339242	16	0.009014141	5	0
Linker_histone	0.000339242	16	0.009014141	5	0
Pfam-B_2659	0.000339242	16	0.009014141	5	0

Pfam-B_2097	0.000416153	3.965246813	0.010800626	14	14
WD40	0.000535201	0.188725376	0.013574652	3	62
HMG_box	0.000928926	9.222087255	0.023037374	7	3
Pfam-B_14494	0.001004204	0.093077819	0.024362871	1	42
Pfam-B_24	0.001328519	0.0625	0.030257706	0	32
Pfam-B_1911	0.001313311	11.84630165	0.030257706	6	2
HSP90	0.001313311	11.84630165	0.030257706	6	2
Pfam-B_3213	0.001708963	0.20917825	0.031265611	3	56
Tubulin-binding	0.00169282	16	0.031265611	5	1
Aldedh	0.001678458	16	0.031265611	4	0
SRPRB	0.001678458	16	0.031265611	4	0
Pfam-B_3205	0.001678458	16	0.031265611	4	0
Pfam-B_7330	0.001678458	16	0.031265611	4	0
TMA7	0.001678458	16	0.031265611	4	0
Pfam-B_1973	0.001678458	16	0.031265611	4	0
Pfam-B_14365	0.001678458	16	0.031265611	4	0
ACBP	0.001678458	16	0.031265611	4	0
Pfam-B_1644	0.001678458	16	0.031265611	4	0
Glycolytic	0.001678458	16	0.031265611	4	0
Pfam-B_2863	0.002108423	0.0625	0.037951613	0	30
Pfam-B_5724	0.002350131	0.10295157	0.041630888	1	38
Pfam-B_743	0.002585364	5.271311938	0.045082282	8	6
Pfam-B_741	0.00279851	4.449440301	0.048048267	9	8
Utp14	0.003240473	0.0625	0.053568835	0	27
Pfam-B_3767	0.003240473	0.0625	0.053568835	0	27
Cpn60_TCP1	0.003264051	7.899135924	0.053568835	6	3
Ribosomal_L14	0.004933175	9.868100083	0.079788745	5	2
Pfam-B_12462	0.005141681	0.0625	0.081973092	0	25
Pfam-B_17350	0.005321264	0.0625	0.083641283	0	26
Pfam-B_9281	0.008164941	0.0625	0.090807969	0	23
KH_1	0.007561801	1.559757252	0.090807969	57	146
LSM	0.00719125	3.558467397	0.090807969	9	10
Ribosomal_L7Ae	0.00719125	3.558467397	0.090807969	9	10
Pfam-B_14992	0.006766738	5.923626238	0.090807969	6	4
Thioredoxin	0.006766738	5.923626238	0.090807969	6	4
Pfam-B_3064	0.006766738	5.923626238	0.090807969	6	4
zf-RanBP	0.006766738	5.923626238	0.090807969	6	4
HnRNP_M	0.007035324	15.77814588	0.090807969	4	1
14-3-3	0.008299653	16	0.090807969	3	0
FTHFS	0.008299653	16	0.090807969	3	0
Pfam-B_3286	0.008299653	16	0.090807969	3	0
Pfam-B_7699	0.008299653	16	0.090807969	3	0
GAS2	0.008299653	16	0.090807969	3	0

WHEP-TRS	0.008299653	16	0.090807969	3	0
Armet	0.008299653	16	0.090807969	3	0
Peptidase_M20	0.008299653	16	0.090807969	3	0
Calponin	0.008299653	16	0.090807969	3	0
Med26	0.008299653	16	0.090807969	3	0
Ndr	0.008299653	16	0.090807969	3	0
Caldesmon	0.008299653	16	0.090807969	3	0
HTH_3	0.008299653	16	0.090807969	3	0
Ldh_1_C	0.008299653	16	0.090807969	3	0
Ldh_1_N	0.008299653	16	0.090807969	3	0
Pfam-B_1356	0.008299653	16	0.090807969	3	0
Tex_N	0.008299653	16	0.090807969	3	0
Pfam-B_6296	0.008299653	16	0.090807969	3	0
PCNP	0.008299653	16	0.090807969	3	0
Pfam-B_17673	0.008299653	16	0.090807969	3	0
Pfam-B_2728	0.008299653	16	0.090807969	3	0
Pfam-B_4483	0.008299653	16	0.090807969	3	0
Brix	0.008464474	0.0625	0.091712167	0	24

Table S2. Related to Figure 2, 3 and 4 and Table S1 and S3.
RBDs enriched in RBDmap LysC and ArgC experiments.

Gene name	Full protein name	Substrate	Class
HIBADH	3-hydroxyisobutyrate dehydrogenase, mitochondrial	NAD/NADH	di-nulceotide
PHGDH	D-3-phosphoglycerate dehydrogenase	NAD/NADH	di-nulcleotide
HADH	Trifunctional enzyme subunit alpha, mitochondrial	NAD/NADH	di-nucleotide
IDH2	Isocitrate dehydrogenase [NADP], mitochondrial	NADP/NADPH	di-nucleotide
NME1	Nucleoside diphosphate kinase A	ATP/ADP	mono-nucleotide
ADK	Adenosine kinase	ATP + adenosine > ADP + AMP	mon-nucleotide
MDH1	Malate dehydrogenase, cytoplasmic	NAD/NADH	di-nucleotide
MDH2	Malate dehydrogenase, mitochondrial	NAD/NADH	di-nucleotide
LDHB	L-lactate dehydrogenase B chain	NAD/NADH	di-nucleotide
ALDH18A1	Delta-1-pyrroline-5-carboxylate synthase	ATP/ADP	mono-nucleotide
ALDH6A1	Methylmalonate-semialdehyde dehydrogenase [acylating], mitochondrial	NAD/NADH	di-nucleotide
ALDH7A1	Alpha-aminoadipic semialdehyde dehydrogenase	NAD/NAHD; NADP/NADPH	di-nucleotide

Table S3. Related to Figure 4 and S4 and Table S2.
List of metabolic enzymes binding mono-nucleotides or di-nucleotides characterized by RBDmap.

PDB id	resolution	LysC data set	ArgC data set
1a9n	2.38	TRUE	TRUE
1aud	NMR		TRUE
1dz5	NMR		TRUE
1e8o	3.2	TRUE	TRUE
1fje	NMR	TRUE	TRUE
1fxl	1.8	TRUE	TRUE
1g2e	2.3	TRUE	TRUE
1k1g	NMR	TRUE	TRUE
1m8y	2.6	TRUE	TRUE
1rgo	NMR	TRUE	
1rkj	NMR	TRUE	TRUE
2adc	NMR	TRUE	TRUE
2fy1	NMR	TRUE	TRUE
2gxb	2.25	TRUE	TRUE
2hyi	2.3	TRUE	TRUE
2i2y	NMR	TRUE	TRUE
2j0q	3.2	TRUE	TRUE
2j0s	2.21	TRUE	TRUE
2kg1	NMR	TRUE	TRUE
2kxn	NMR	TRUE	TRUE
2l3j	NMR	TRUE	
2leb	NMR	TRUE	TRUE
2lec	NMR	TRUE	TRUE
2m8d	NMR	TRUE	TRUE
2py9	2.56	TRUE	TRUE
2rs2	NMR	TRUE	
2vod	2.1	TRUE	TRUE
2xb2	3.4	TRUE	TRUE
2xzm	3.93	TRUE	TRUE
2xzn	3.93	TRUE	TRUE
2xzo	2.4	TRUE	TRUE
2y9a	3.6	TRUE	TRUE
2y9b	3.6	TRUE	
2y9c	3.6	TRUE	TRUE
2y9d	3.6	TRUE	
2yh1	NMR	TRUE	TRUE
3a6p	2.92	TRUE	
3adl	2.2	TRUE	
3d2s	1.7	TRUE	TRUE
3ex7	2.3	TRUE	TRUE
3g9y	1.4	TRUE	TRUE
3nnc	2.2	TRUE	TRUE

3o2z	4	TRUE	TRUE
3o30	4	TRUE	TRUE
3o58	4	TRUE	TRUE
3o5h	4	TRUE	TRUE
3q0q	2	TRUE	TRUE
3q0r	2	TRUE	TRUE
3q0s	2	TRUE	TRUE
3q2t	3.06		TRUE
3rc8	2.9	TRUE	TRUE
3rw6	2.3	TRUE	TRUE
3siv	3.3	TRUE	TRUE
3snp	2.8	TRUE	
3ts2	2.01	TRUE	
3vyx	2.29	TRUE	TRUE
4b3g	2.85	TRUE	
4b8t	NMR	TRUE	TRUE
4boc	2.65	TRUE	TRUE
4bpe	3.7	TRUE	TRUE
4bpn	3.703	TRUE	TRUE
4bpo	3.7	TRUE	TRUE
4bpp	3.7	TRUE	TRUE
4ed5	2	TRUE	TRUE
4f02	2	TRUE	TRUE
4f3t	2.25	TRUE	TRUE
4krf	2.1	TRUE	

Table S5. Related to Figure 2 and 3.
List of PDB protein-RNA structures used for RBDmap validation.

ADDITIONAL FIGURE LEGENDS

Table S1. Related to Figure 1 and Figure S1.
List of RBDs and their respective peptides, identified by RBDmap.

Table S4. Related to Figure 6.
Mendelian mutations occurring within the RNA-bound fragments of RBPs and their associated diseases.

SUPPLEMENTAL EXPERIMENTAL PROCEDURES

Considerations regarding the design of RBDmap

RBDmap was designed to offer the following advances over existing methods: 1) identification of the domains of RBPs engaged with RNA in living cells, offering high-resolution RBD maps. 2) Characterization of hundreds of RBPs on a proteome-wide scale, providing the capacity for RBD "discovery" from both well-established RBPs and proteins previously unrelated to RNA. RBDmap scores endogenous protein-RNA interactions in a physiological context, since native protein-RNA pairs are covalently linked upon irradiation of cell monolayers. Note that UV crosslinking can only occur between nucleotides and amino acids in direct contact. In contrast to chemical crosslinking, UV crosslinking does not promote detectable protein-protein crosslinks (Figure S1A, Figure S4A) (Castello et al., 2013b; Pashev et al., 1991; Strein et al., 2014). 3) Protein-RNA co-structures greatly contributed to understanding protein-RNA interactions mediated by globular protein domains. Conversely, disordered domains represent a challenge for crystallization approaches. Because RBDmap can define RBDs within both globular and disordered regions, it complements structural studies. Moreover, RBDmap can be used to instruct CLIP-seq approaches by providing the RNA-binding profiles for many RBPs of interest. 4) RBDmap is here applied to steady state cell cultures, but it can be used to study in a system-wide manner the plasticity of RBDs in response to physiological alterations. 5) RBDmap further validates hundreds of novel RBPs discovered by human RNA interactome studies (Figure 1G) (Baltz et al., 2012; Castello et al., 2012) and assigns them a RNA-protein interface. It is important to highlight that the buffers used here include high salt (500 mM LiCl) and chaotropic detergents (0.5% LiDS) that efficiently remove non-covalent binders from purified RNA (Baltz et al., 2012; Castello et al., 2012; Castello et al., 2013b), as illustrated by the low protein content present in non-irradiated samples . RBDmap applies protease digestion to identify RBDs. This generates peptides of ~17 amino acids (Figure 1A), disrupting protein-protein interactions that might have withstood the stringent washing conditions.

Note that RBDmap does not cover all the proteins identified by RNA interactome capture (Figure 1G). Although experimentally related, RNA interactome capture and RBDmap differ in key aspects that may affect peptide identification by MS. Compared to RNA interactome capture, RBDmap includes a protease (LysC or ArgC) treatment prior to a second oligo(dT) purification step, as described above (Figure 1A). These additional steps reduce sample complexity and background level, facilitating the identification of additional peptides (Figure 1H). On the other hand, RBDmap may fail to assign RNA-binding sites to a number of proteins detected by RNA interactome capture for the following reasons: 1) LysC/ArgC treatment can impair peptide identification when the resulting RNA-bound peptide is identical to the tryptic peptide and no "neighboring" MS-detectable peptide can be released after trypsin treatment. Due to the frequent occurrence of arginines and lysines in RBPs, these cases may not be infrequent. 2) The two-round purification workflow of RBDmap causes increased material loss compared to RNA interactome capture and, indeed, we find that RNA recovery is reduced to about 60%. Therefore, the reduction in background described above is also accompanied with a decrease in signal. 3) We apply highly stringent statistical criteria to report a peptide as an RBDpep. The coverage of the HeLa RNA interactome would be much higher if "CandidateRBDpeps" [10% false discovery rate (FDR) instead of 1% FDR] would also be considered. Taking this set of peptides into account, RBDmap would cover most of the RBPs reported in the HeLa RNA interactome. However, to minimize the incidence of wrongly assigned RBDs (false positives), we opted to apply highly stringent 1% FDR cut-off. Since "candidateRBDpeps" could provide valuable information, this dataset is accessible in Table S1 and online (http://www-huber.embl.de/users/befische/RBDmap).

Selection of the first protease for RBDmap

An *in silico* digest of all protein sequences of the HeLa mRNA interactome (Castello et al., 2012) provided a set of theoretical proteolytic fragments for each of the eleven proteases commonly used in proteomics. Tryptic peptides identified in the HeLa mRNA interactome were mapped onto the proteolytic fragments predicted for each protease. We set a theoretical RNA-binding site in the center of the protein and monitored the number of cases where the protease fragment covers the theoretical binding site. The

RBDmap resolution for each protease was determined as the number of proteins for which a given protease can narrow down the RNA-binding site to less than 20% of the actual protein length. LysC and ArgC were identified as the proteases that theoretically would perform better in a higher number of proteins of the HeLa RNA interactome. However, other proteases may outcompete LysC and ArgC in a case-dependent manner.

The RBDmap protocol

HeLa cells were grown overnight on six 500cm^2 dishes in DMEM medium supplemented with 10% fetal calf serum. Three of the plates were incubated overnight with 100 µM 4-thiouridine (4SU) for PAR-CL. After PBS wash, 0.15 J/cm^2 UV light at 254nm (for cCL) was applied on untreated cell monolayers (3 dishes) and 365nm (for PAR-CL) on 4SU-treated cell monolayers (3 dishes), as previously described (Castello et al., 2013b). Cells were harvested and lysed in a buffer containing 20mM pH 7.5 Tris HCl, 500mM LiCl, 0.5% LiDS, 1mM EDTA and 5 mM DTT and homogenized by passing the sample through a syringe with a narrow gauge needle (0.4 mm diameter). Proteins crosslinked to poly(A)$^+$ mRNAs were captured with oligo(dT)$_{25}$ magnetic beads (NE Biolabs). Subsequently, oligo(dT)$_{25}$ beads were washed with buffers containing decreasing concentrations of LiCl and LiDS, as previously described (Castello et al., 2013b). RNAs and crosslinked proteins were eluted with 20mM Tris HCl, pH 7.5 at 55°C for 3 min. 70 µl were taken for RNA and protein quality controls as previously described (Castello et al., 2013b). For RNA analysis, samples were digested with proteinase K, followed by RNA isolation with RNeasy (Qiagen). The remaining sample was treated with 1µg of LysC or ArgC, and supplemented with 1 µl of RNaseOUT (Promega) and 5x of the protease buffer as described by the manufacturer. After digestion at 37°C for 8h, 70 µl were taken for RNA and protein quality controls as described (Castello et al., 2013b). 1/3 of the sample from irradiated and non-irradiated cells was taken for mass spectrometry (input) and processed as indicated below. The rest of the sample was diluted 2 ml of 5x dilution buffer (2.5 M LiCl, 100mM pH 7.5 Tris HCl, 5 mM EDTA and 25 mM DTT) and H_2O (10 ml total volume), and incubated with 2 ml of oligo(dT) beads for 1 h. After separating the beads with a magnet, the supernatant was collected and kept at 4°C (released fraction). Beads are washed once with 500mM LiCl and 0.5% LiDS containing buffer, and with buffers containing decreasing concentrations of LiCl and LiDS as previously described (Castello et al., 2013b). The RNA-bound fraction is eluted with 20mM Tris HCl, pH 7.5 for 3 min at 55°C. All input, supernatant (released) and eluates (RNA-bound) are treated with RNase T1 and RNase A (Sigma). Samples were then processed for MS as described below.

Sample preparation for MS

Samples were processed according to standard protocols (Wisniewski et al., 2009) with minor modifications. Cysteines were reduced (5 mM DTT, 56˚C, 30 min) and alkylated (10 mM Iodoacetamide, 30 min in the dark). Samples were buffer-exchanged into 50 mM triethylammoniumbicarbonate, pH 8.5, using 3 kDa centrifugal filters (Millipore) and digested with sequencing grade trypsin (Promega, enzyme-protein ratio 1:50) at 37˚C for 18 h. Resulting peptides were desalted and labelled using stable isotope reductive methylation (Boersema et al., 2009) on StageTips (Rappsilber et al., 2007). Labels were swapped between replicates. Labeled samples were combined and fractionated into 12 fractions on an 3100 OFFGEL Fractionator (Agilent) using Immobiline DryStrips (pH 3–10 NL, 13 cm; GE Healthcare) according to the manufacturer's protocol. Isoelectric focusing was carried out at a constant current of 50 mA allowing a maximum voltage of 8000 V. When 20 kVh were reached the fractionation was stopped, fractions were collected and desalted using StageTips. Samples were dried in a vacuum concentrator and reconstituted in MS loading buffer (5% DMSO 1% formic acid).

LC-MS/MS

Samples were analyzed on a LTQ-Orbitrap Velos Pro mass spectrometer (Thermo Scientific) coupled to a nanoAcquity UPLC system (Waters). Peptides were loaded onto a trapping column (nanoAcquity Symmetry C_{18}, 5 µm, 180 µm × 20 mm) at a flow rate of 15 µl/min with solvent A (0.1% formic acid). Peptides were separated over an analytical column (nanoAcquity BEH C18, 1.7 µm, 75 µm × 200 mm) using a 110 min linear gradient from 7-40% solvent B (acetonitrile, 0.1% formic acid) at a constant flow rate of 0.3 µl/min. Peptides were introduced into the mass spectrometer using a Pico-Tip Emitter (360 µm outer diameter × 20 µm inner diameter, 10 µm tip, New Objective). MS survey scans were acquired from 300–1700 *m*/*z* at a nominal resolution of 30000. The 15 most abundant peptides were isolated within a 2 Da window and subjected to MS/MS sequencing using collision-induced dissociation in the ion trap (activation time 10 msec, normalized collision energy 40%). Only 2+/3+ charged ions were included for analysis. Precursors were dynamically excluded for 30 sec (exclusion list size was set to 500).

Peptide identification and quantification

Raw data were processed using MaxQuant (version 1.3.0.5) (Cox and Mann, 2008). MS/MS spectra were searched against the human UniProt database (version 12_2013) concatenated to a database containing protein sequences of common contaminants. Enzyme specificity was set to trypsin/P, allowing a maximum of two missed cleavages. Cysteine carbamidomethylation was set as fixed modification, and methionine oxidation and protein N-terminal acetylation were used as variable modifications. The minimal peptide length was set to six amino acids. The mass tolerances were set to 20 ppm for the first search, 6 ppm for the main search and 0.5 Da for product ion masses. False discovery rates for peptide and protein identification were set to 1%. Match between runs (time window 2 min) and re-quantify options were enabled.

Statistical Analysis

To identify the "input" peptides, the intensity of peptides in crosslinked was compared to non-crosslinked samples after oligo(dT) capture. To test whether the log2-intensity ratio of each peptide in three replicated experiments is different from zero, p-values were computed by a moderated t-test implemented in the R/Bioconductor package limma (Smyth, 2004). p-values were corrected for multiple testing by controlling the false discovery rate with the method of Benjamini-Hochberg. A peptide set with a false discovery rate (FDR) of 1% was used for further analysis.

To identify RNA-binding sites, the log2 intensity ratio in the RNA-bound to the released fraction was considered. The distribution of the log2-ratios is bi-modal, representing the released and RNA-bound peptides. The log2-ratios are normalized to the location of the left mode using a robust estimate. Log2-ratios of each peptide in three replicate experiments were tested against zero by a moderated t-test from the R/Bioconductor package limma (Smyth, 2004), and p-values were corrected for multiple testing by the method of Benjamini-Hochberg. Peptides with a 1% FDR are termed 'RBDpep'. Peptides extending this set to a 10% FDR are called 'CandidateRBDpep'. For further analysis and to identify the protein set covered by these peptides, only peptides uniquely mapping to a gene model are considered.

Computational validation of identified binding sites by correlation with domain annotations

To validate the identified binding sites and to distinguish them from non-binding sites, all proteins with at least one RBDpep covering a classical RBD and one RBDpep mapping outside a classical RBD were considered. RBDpeps were sorted by their log2- RNA-bound/released intensity ratios. For each window of 101 peptides, comprising the RBDpep under consideration plus 50 peptides on either side of this viewpoint, the probability that the RBDpep is within a classical RBD were considered. The probability that the RBDpep is within a classical RBD is computed as the fraction of RBDpeps that cover classical RBDs over the fraction of peptides mapping outside the RBD.

RBD maps: data display and interpretation

MS-identified tryptic peptides enriched in the RNA-bound or released fractions, respectively, are mapped back to proteins and extended to the two adjacent LysC or ArgC cleavage sites to recall the original proteolytic fragment. LysC and ArgC proteolytic fragments are plotted regarding their position within the protein (x axis: N- to C-termini) and their fold change between the RNA-bound and released fractions (y axis), as exemplified in Figure 2D. 1% FDR RBDpeps and 10% FDR candidateRBDpeps are shown in red and salmon, respectively, while released fragments are shown in blue. Boxes below the plot are used to visualize the position of the protein's domains.

Frequently, a given domain is mapped by multiple RBDpeps, reflecting the reliability of RBDmap. In some instances two proteolytic fragments overlap partially or almost completely but display different RNA-bound/released fold changes. Because we only use uniquely mapped peptides, overlapping peptides can be explained as follows: 1) The peptides are non-identical (i.e. one or two amino acids longer or shorter). This can occur when the protease encounters multiple cleavage sites adjacent to each other, allowing differential proteolysis. Since proteases require a number of amino acids on both sides of the scission site, cleavage at a given amino acid may abrogate cleavage at an adjacent site. 2) The two peptides are generated by different proteases. To facilitate the interpretation we indicate the protease from which it originates (L for LysC; A for ArgC) adjacent to the RBDpep. In the online version (http://www-huber.embl.de/users/befische/RBDmap), the identity of the protease can be seen by passing the cursor over the peptide line. In most cases, overlapping LysC and ArgC fragments exhibit comparable RNA-bound/released ratios, confirming the same RNA-binding sites within a protein with two independent proteases. As a general rule, the shorter RBDpep provides the higher resolution. However, in rare cases, a given region can be found to be RNA-bound with one protease and released with the other. This outcome implies that one of the peptides harbors the RNA-binding site, thus qualifying as RBDpep, and the other does not.

To integrate data from homologous and non-homologous proteins, we classified the proteins based on the domains identified as RBDs (e.g. FKBP protein family). We aligned the domain exhibiting RNA-binding activity (e.g. FKBP fold) from homologs and non-homologs harboring it. The relative position of each RBDpep was extracted and plotted as a "block". The number of independent peptide "blocks" accumulated at a given position reflects the prevalence of an RNA-binding site across the proteins sharing the same domain (e.g. Figure S2A). RBD classification can be visualized and browsed under "globular domains" on the website http://www-huber.embl.de/users/befische/RBDmap/.

Characterization of RBDpeps

Domain enrichment. For gene set enrichment analysis of RBDs, we used the Pfam domain annotation (Finn et al., 2014) in the Interpro database (Hunter et al., 2012; McDowall and Hunter, 2011). For each identified LysC/ArgC proteolytic fragment in the RNA-bound fraction or in the input, we scored whether it overlaps with a Pfam domain or not. Fisher's exact test was used to compute p-values for enrichment. p-values were corrected for multiple testing by the method of Benjamini-Hochberg. Pfam domains with a false discovery rate of 10% are reported.

Identification of disordered fragments. The intrinsically unstructured or disordered parts of a protein were predicted by "iupred" (Dosztanyi et al., 2005). Amino acids with an iupred score of >0.4 were considered as being present in a disordered region. A proteolytic fragment of identified peptides is regarded as disordered, if the average iupred score is larger than 0.4.

Amino acid composition. The amino acid composition of all RBDpep or released fragments is compared to the amino acid composition of all input fragments. For analysis of disordered or globular RNA-binding sites, RNA-bound or released proteolytic fragments overlapping with disordered or globular protein segments were compared to disordered or globular input fragments. Over-/underrepresentation of a given amino acid was tested by Fisher's exact test, and p-values were corrected for multiple testing by the method of Benjamini-Hochberg.

Tripeptide enrichment. p-values for motif enrichment of triplet amino acids were computed by a binomial test using the fraction of the total length of all RBDpep fragments over the total length of all fragments as the hypothesized probability of success. P-values were Benjamini-Hochberg corrected for multiple testing.

Motif alignment. To identify specific sequences that occur within disordered RNA-binding sites, the RBDmap fragments were mapped onto the proteins. The detected RNA-binding sites were dissected into half-overlapping sequences of a maximum length of 11 amino acids. The multiple sequence alignment software clustal omega (Release 1.2.0) (Sievers et al., 2011) was used for multiple sequence alignment. The cluster tree is cut at h=10. Sequences within each cluster were aligned again. Sequence logos showing the information content of each amino acid position were plotted with weblogo (Release 3.3) (Crooks et al., 2004) for each cluster. The amino acid composition of the input fragments was used as background. Prevalent amino acids in the motif logo may bind RNA or be involved in other functions such as binding regulation (e.g. PTM) or disorder promotion (e.g. G, S and P).

Posttranslational modifications. Annotations of post-translational modifications (PTMs) were downloaded from Uniprot (Release 2013_12). PTM enrichment analysis was performed as for Pfam domains (see above). The amino acid enrichment in a window of +/- 6 amino acids around the PTM was computed for RNA-bound and input fragments. Sequence logos showing the relative entropy of the amino acid compositions were plotted.

Disease-associated mutations. Sequence variants associated with diseases from OMIM (Brandt, 1993; Castello et al., 2013a) and natural sequence variants were downloaded from Uniprot (Release 2013_12). Variants overlapping with RNA-bound or released proteolytic fragments were classified into disease-associated or non-pathological. Statistical significance of enrichment of disease variants in RNA-bound fragments was assessed by Fisher's exact test.

RBP abundance and isoelectric point: the mean normalized mRNA level over 16 arrays of HeLa cells extracted from the ArrayExpress atlas (ArrayExpress accession E-MTAB-62) was used to assess the mRNA levels of proteins within the HeLa whole proteome, RNA interactome, input fraction and RBDmap dataset. This approach was also employed to infer the abundance of previously known RBPs as well as proteins harboring novel globular or disordered RBDs. The isoelectric point (Ip) implemented in the *trans* proteomic pipeline was used to analyzed the Ip distribution of these protein groups.

RBDpep conservation: RNA-bound and released LysC/ArgC fragments were aligned to the whole proteomes of *Danio rerio*, *Drosophila melanogaster*, *Caenorhabditis elegans*, and *Saccharomyces cerevisiae* (UniProt release 2015_01) using BLASTP 2.2.26. A fragment was classified as conserved, if it matches a protein with an e-value <= 1. The fraction of RNA-bound peptides beyond all conserved peptides are tested against the hypothesis that it is equal to the fraction of RNA-bound peptides beyond all fragments (RNA-bound and released) using a binomial test.

Validation of identified binding sites based on PDB structures

For computational validation of RBDmap data, 3D structures of protein-RNA complexes deposited in the PDB databank were used (downloaded October 2013). Only NMR and x-ray diffraction structures with resolutions better than 5.0 Å were considered. Protein sequences in PDB structures were first aligned with RBDmap LysC and ArgC fragments (10% FDR) matching 305 human UniProt protein sequences (version 12_2013). We used local Smith-Waterman alignment (EMBOSS water, gap open penalty 10.0, gap extend penalty 0.5, EBLOSUM90). Because reported structures may contain short deletions and to allow alignment with highly conserved protein-RNA interactions from different organisms, hits with identity larger than 70% and with gaps less than 10% of the reported aligned region were considered further. This resulted in a total of 67 structures containing protein-RNA complexes (64 and 56 structures for LysC and ArgC, respectively, see Table S5), which were used for computational validation of RBDmap data.

Protein sequences of selected 3D structures were then segmented in silico into LysC or ArgC fragments, respectively. For each fragment, the distance from the closest amino acid (β-carbon) to the RNA atoms was calculated. Fragments at distances closer than 4.3 Å to RNA were classified as proximal (49% for LysC, 57% for ArgC), all others as non-proximal. Because most of the polypeptides used in these studies represent only the RBD of the protein (e.g. RRM of PABPC1), we observe a bias towards proximal peptides. Indeed, about half of the LysC and ArgC proteolytic fragments are classified as proximal (Fig 1k and Extended Data 2). However, the overlap between proximal fragments and RBDpeps is 70% for LysC and 81% for ArgC, implying that RBDmap highly significantly enriches for peptides in close proximity with the RNA. Significance of the overlap was calculated with the hypergeometric test.

Generation of high-resolution profiles using the ratio of X-link over released peptide coverage

We collected the superset of RBDpeps and released peptides mapping to each RBD type. The position of these peptides within the domain or in upstream or downstream protein regions is mapped to a linear scale from -1 to 2, with the RBD itself spanning the range from 0 to 1 and the flanking regions from -1 to 0 and from 1 to 2, respectively. We then subtracted the MS-identified portion of RBDpeps and released fragments. Through MS-identified N-link peptide removal we can infer the X-link moiety which represents the actual RNA-binding portion, as described in Figure 1A. Domain profiles in Figure 3B-E are generated by calculating the ratio between X-link and released peptide coverage at each position of the domain. To compute the ratio, a pseudo count of 3 was added to avoid artifacts with low count numbers. For normalization, the computed ratio was divided by the ratio between the complete pool of X-link peptides and RNA-released peptides mapping to the complete set of proteins harboring the domain under study, resulting in the displayed fold change.

Secondary structure was predicted using NetSurfP (version 1.1, (Petersen et al., 2009)) for the same protein domains and mapped to the scale of -1 to 2 as above. Thereby the probabilities for alpha-helices and beta-strands were linearly approximated. The profiles display the mean of all predictions.

Establishment of stable HeLa cell lines

Chimeric cDNAs obtained by PCR from a HeLa cDNA library were used as template for PCR. Inserts were inserted into pCDNA5/FRT/TO- eGFP (Strein et al., 2014) or pCDNA5/FRT/TO-FLAG-HA. These plasmids include a glycine(G)-serine(S) (GGSGGSGG) linker between the tag and the protein of interest. Generation of the stable cell lines was performed as described in the manufacturer's protocol (Flp In TRex, Invitrogen). Protein is induced by addition of tetracycline as described elsewhere (Castello et al., 2012).

Interactome capture for eGFP-tagged proteins

1x500 cm^2 dish at 50% confluence of HeLa TRex cells expressing the different eGFP-fusion proteins were induced overnight with 1µg/ml tetracycline. Cell monolayers were irradiated with 0.15 J/cm^2, 254 nm UV light, and lysed into 500 mM and 0.5% LiDS-containing buffer as in (Castello et al., 2013b). $Poly(A)^+$ RNAs and crosslinked proteins were captured with 500 µl of $oligo(dT)_{25}$ magnetic beads. Subsequently, $oligo(dT)_{25}$ beads were washed with buffers containing decreasing concentrations of LiCl and LiDS, as previously described (Strein et al., 2014). After elution into 20mM Tris HCl, pH 7.5 at 55°C for 3 min, eluates were concentrated in a 3KDa amicon device to 20µl final volume, cooled at 4°C for 10 min, and loaded in a 96 well plate with transparent bottom. eGFP measurement was performed as in (Strein et al., 2014).

PNK assay

Cells expressing FLAG-HA fusion proteins were UV-crosslinked on ice (150 mJ/cm^2), lysed (500 mM NaCL, 30 mM Tris pH 7.5, 5% glycerol, 0.1% Triton X-100, 2 mM Mg_2Cl, 4 mM β-mercaptoethanol and protease inhibitors), and homogenized passing the lysate through a narrow needle (22G) followed by pulsed ultrasonication (3 × 10 s, 50% amplitude, on ice). Cleared lysates were treated with 50 U/ml DNAseI (Takara) and RNaseI for 15 min at 37 °C, and used for immunoprecipitation with FLAG M2 coupled to magnetic beads (M8823, Sigma) for 2h at 4°C. Beads were washed once with lysis buffer and five times with wash buffer (100 mM NaCL, 30 mM Tris pH 7.5, 5% glycerol, 0.1% Triton X-100, 2 mM Mg_2Cl, 4 mM β-mercaptoethanol and protease inhibitors). RNA crosslinked to the tagged RBD is identified by radiolabeling with 0.1 µCi/µl γ-32P ATP by T4 polynucleotide kinase (1U/µl) in PNK buffer (50 mM NaCL, 50 mM Tris pH 7.5, 0.5% NP-40, 10 mM Mg_2Cl and 5 mM DTT) for 15 min at 850 rpm and 37°C. Beads were washed four to six times with PNK buffer and protein-RNA complexes were eluted with a 3-fold excess of FLAG peptide. Samples were analyzed by SDS PAGE and autoradiography.

Immunoprecipitation of eGFP and YFP fusion proteins

One 10 cm dish of Tet-inducible cell lines expressing eGFP- or YFP-tagged proteins were treated with tetracycline overnight and cell monolayers were UV irradiated (150 mJ/cm^2) on ice after two PBS washes. Cells were lysed with GBP (GFP-binding protein) lysis buffer (500 mM NaCl, 20 mM pH 7.5 Tris-HCl, 2 mM Mg2Cl, 0.025% SDS, 0.1% Triton X-100 and protease inhibitors). Cell lysates were homogenized by passing them through a narrow needle (22G). Extracts were incubated with 10 µl of equilibrated GFP_trap_A (Chromotek) for 2h at 4°C. Beads were washed three times with GBP lysis buffer and 3 times with GBP wash buffer (150 mM NaCl, 20 mM pH 7.5 Tris-HCl, 2 mM Mg2Cl, 0.01% Triton X-100 and protease inhibitors). Proteins were eluted with loading buffer for 5 min at 95°C. Eluates were analysed by SDS PAGE followed by silver staining (see below).

Silver staining analysis

Proteins co-isolated by oligo(dT) pull down or in immunoprecipitation experiments were analyzed by silver staining, according to standard protocols (Castello et al., 2012).

Data dissemination

The mass spectrometry proteomics data have been deposited with the ProteomeXchange Consortium (http://www.proteomexchange.org) via the PRIDE partner repository (Vizcaino et al., 2013) with the dataset identifier PXD000883".

The details:

ProteomeXchange title: RBDmap

ProteomeXchange accession: PXD000883

Reviewer account:

- Username: reviewer46276@ebi.ac.uk
- Password: xg0ioRX5
- To access the data please visit: http://tinyurl.com/pu7yodo

RBDmap analyses and protein profiles can be visualised at:

http://www-huber.embl.de/users/befische/RBDmap

SUPPLEMENTAL REFERENCES

Arif, A., Jia, J., Mukhopadhyay, R., Willard, B., Kinter, M., and Fox, P.L. (2009). Two-site phosphorylation of EPRS coordinates multimodal regulation of noncanonical translational control activity. Mol Cell *35*, 164-180.

Boersema, P.J., Raijmakers, R., Lemeer, S., Mohammed, S., and Heck, A.J. (2009). Multiplex peptide stable isotope dimethyl labeling for quantitative proteomics. Nat Protoc 4, 484-494.

Brandt, K.A. (1993). The GDB Human Genome Data Base: a source of integrated genetic mapping and disease data. Bull Med Libr Assoc 81, 285-292.

Cox, J., and Mann, M. (2008). MaxQuant enables high peptide identification rates, individualized p.p.b.-range mass accuracies and proteome-wide protein quantification. Nat Biotechnol 26, 1367-1372.

Crooks, G.E., Hon, G., Chandonia, J.M., and Brenner, S.E. (2004). WebLogo: a sequence logo generator. Genome Res 14, 1188-1190.

Chakrabarti, S., Jayachandran, U., Bonneau, F., Fiorini, F., Basquin, C., Domcke, S., Le Hir, H., and Conti, E. (2011). Molecular mechanisms for the RNA-dependent ATPase activity of Upf1 and its regulation by Upf2. Mol Cell 41, 693-703.

Dosztanyi, Z., Csizmok, V., Tompa, P., and Simon, I. (2005). IUPred: web server for the prediction of intrinsically unstructured regions of proteins based on estimated energy content. Bioinformatics 21, 3433-3434.

Finn, R.D., Bateman, A., Clements, J., Coggill, P., Eberhardt, R.Y., Eddy, S.R., Heger, A., Hetherington, K., Holm, L., Mistry, J., et al. (2014). Pfam: the protein families database. Nucleic Acids Res 42, D222-230.

Hunter, S., Jones, P., Mitchell, A., Apweiler, R., Attwood, T.K., Bateman, A., Bernard, T., Binns, D., Bork, P., Burge, S., et al. (2012). InterPro in 2011: new developments in the family and domain prediction database. Nucleic Acids Res 40, D306-312.

Kelley, L.A., and Sternberg, M.J. (2009). Protein structure prediction on the Web: a case study using the Phyre server. Nat Protoc 4, 363-371.

McDowall, J., and Hunter, S. (2011). InterPro protein classification. Methods Mol Biol 694, 37-47.

Mukhopadhyay, R., Ray, P.S., Arif, A., Brady, A.K., Kinter, M., and Fox, P.L. (2008). DAPK-ZIPK-L13a axis constitutes a negative-feedback module regulating inflammatory gene expression. Mol Cell 32, 371-382.

Petersen, B., Petersen, T.N., Andersen, P., Nielsen, M., and Lundegaard, C. (2009). A generic method for assignment of reliability scores applied to solvent accessibility predictions. BMC Struct Biol 9, 51.

Rappsilber, J., Mann, M., and Ishihama, Y. (2007). Protocol for micro-purification, enrichment, pre-fractionation and storage of peptides for proteomics using StageTips. Nat Protoc 2, 1896-1906.

Sievers, F., Wilm, A., Dineen, D., Gibson, T.J., Karplus, K., Li, W., Lopez, R., McWilliam, H., Remmert, M., Soding, J., et al. (2011). Fast, scalable generation of high-quality protein multiple sequence alignments using Clustal Omega. Mol Syst Biol 7, 539.

Smyth, G.K. (2004). Linear models and empirical bayes methods for assessing differential expression in microarray experiments. Stat Appl Genet Mol Biol 3, Article3.

Vizcaino, J.A., Cote, R.G., Csordas, A., Dianes, J.A., Fabregat, A., Foster, J.M., Griss, J., Alpi, E., Birim, M., Contell, J., et al. (2013). The Proteomics Identifications (PRIDE) database and associated tools: status in 2013. Nucleic Acids Research 41, D1063-D1069.

Wisniewski, J.R., Zougman, A., Nagaraj, N., and Mann, M. (2009). Universal sample preparation method for proteome analysis. Nat Methods 6, 359-362.
