# Supplementary material for: Comprehensive Identification of RNA-Binding Domains in Human Cells
Source: Mol Cell. 2016 Aug 18;63(4):696–710. doi: 10.1016/j.molcel.2016.06.029 (PMC5003815; doi:10.1016/j.molcel.2016.06.029)
Supplement: Document S2. Article plus Supplemental Information [file mmc4.pdf]

Resource

Molecular Cell

Comprehensive Identification of RNA-Binding Domains in Human Cells

Graphical Abstract

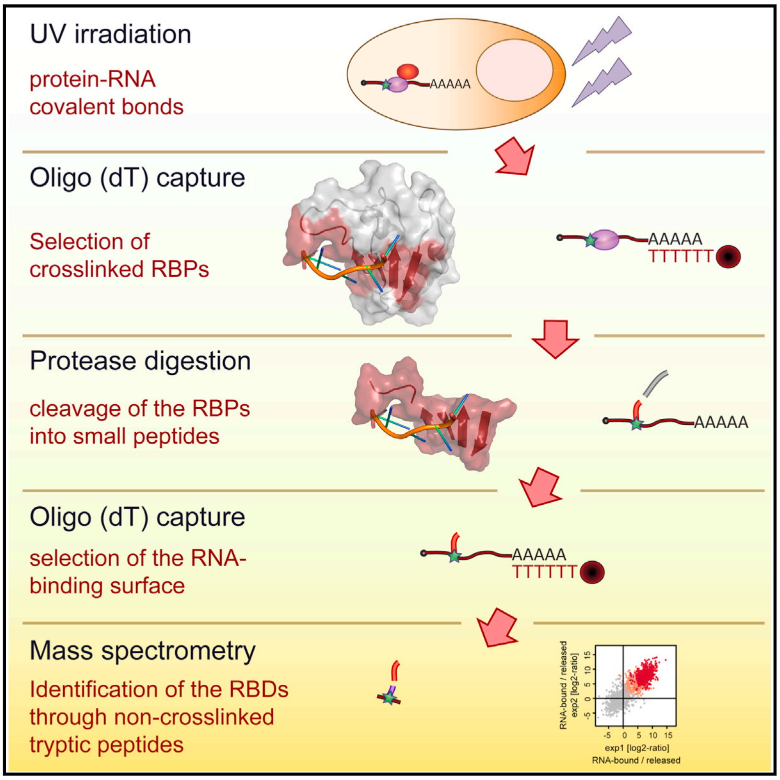

Highlights

- Experimental generation of an atlas of RNA-binding sites (RBS) in human cells
- RBS overlap with enzymatic cores and protein-protein interaction sites
- About half of the total RBS map to disordered protein regions
- RBS are enriched for phosphorylation, acetylation, and methylation sites

Authors

Alfredo Castello, Bernd Fischer, Christian K. Frese, ..., Tomaz Curk, Jeroen Krijgsveld, Matthias W. Hentze

Correspondence

hentze@embl.de

In Brief

Many recently discovered RNA-binding proteins (RBPs) do not show architectural similarities with classical RBPs, and their modes of interaction with RNA were unclear. We developed and employed RBDmap as a method for the comprehensive determination of the RNA-interacting sites of RBPs, identifying more than a thousand such sites. These data yield unprecedented insight into RNA-protein interactions in cells with implications for numerous biological contexts.

Accession Numbers

PXD000883

Castello et al., 2016, Molecular Cell *63*, 696–710
August 18, 2016 © 2016 The Author(s). Published by Elsevier Inc.
http://dx.doi.org/10.1016/j.molcel.2016.06.029

CellPress

Molecular Cell
Resource

Comprehensive Identification of RNA-Binding Domains in Human Cells

Alfredo Castello,[1,2,5] Bernd Fischer,[1,3,5] Christian K. Frese,[1] Rastislav Horos,[1] Anne-Marie Alleaume,[1] Sophia Foehr,[1] Tomaz Curk,[1,4] Jeroen Krijgsveld,[1,3] and Matthias W. Hentze[1,*]
[1]European Molecular Biology Laboratory (EMBL), Meyerhofstrasse 1, 69117 Heidelberg, Germany
[2]Department of Biochemistry, University of Oxford, South Parks Road, Oxford OX1 3QU, UK
[3]German Cancer Research Center (DKFZ), Im Neuenheimer Feld 280, 69120 Heidelberg, Germany
[4]Faculty of Computer and Information Science, University of Ljubljana, 1001 Ljubljana, Slovenia
[5]Co-first author
*Correspondence: hentze@embl.de
http://dx.doi.org/10.1016/j.molcel.2016.06.029

SUMMARY

Mammalian cells harbor more than a thousand RNA-binding proteins (RBPs), with half of these employing unknown modes of RNA binding. We developed RBDmap to determine the RNA-binding sites of native RBPs on a proteome-wide scale. We identified 1,174 binding sites within 529 HeLa cell RBPs, discovering numerous RNA-binding domains (RBDs). Catalytic centers or protein-protein interaction domains are in close relationship with RNA-binding sites, invoking possible effector roles of RNA in the control of protein function. Nearly half of the RNA-binding sites map to intrinsically disordered regions, uncovering unstructured domains as prevalent partners in protein-RNA interactions. RNA-binding sites represent hot spots for defined posttranslational modifications such as lysine acetylation and tyrosine phosphorylation, suggesting metabolic and signal-dependent regulation of RBP function. RBDs display a high degree of evolutionary conservation and incidence of Mendelian mutations, suggestive of important functional roles. RBDmap thus yields profound insights into native protein-RNA interactions in living cells.

INTRODUCTION

RNA metabolism relies on the dynamic interplay of RNAs with RNA-binding proteins (RBPs) forming ribonucleoprotein complexes, which control RNA fate from synthesis to decay (Glisovic et al., 2008). Due to their central role in cell biology, it is unsurprising that mutations in RBPs underlie numerous hereditary diseases (Castello et al., 2013a; Lukong et al., 2008).

Many RBPs are modular, built from a limited pool of RNA-binding domains (RBDs), including the RNA recognition motif (RRM) and other canonical RBDs (Lunde et al., 2007). These domains have been characterized biochemically and structurally, furthering our understanding of protein-RNA interactions. The identification of unorthodox RBPs lacking canonical RBDs expands the scope of physiologically important protein-RNA interactions (e.g., Jia et al., 2008).

System-wide approaches to identify RBPs have recently been developed, including immobilization of RNA probes (Butter et al., 2009) or proteins (Scherrer et al., 2010; Tsvetanova et al., 2010), followed by in vitro selection of their interaction partners. These experiments identified numerous proteins previously unknown to bind RNA. While informative, in vitro protein-RNA interactions may arise non-physiologically from the electrostatic properties of RNA. To address this limitation, in vivo UV crosslinking has been used to covalently stabilize native protein-RNA interactions occurring in living cells. After cell lysis, proteins covalently bound to polyadenylated [poly(A)] RNAs are isolated by oligo(dT) selection and identified by quantitative mass spectrometry (Baltz et al., 2012; Castello et al., 2012). This approach (named RNA interactome capture) identified over a thousand RBPs in HeLa and HEK293 cells, hundreds of which were previously unknown to bind RNA. Subsequently, similar data sets were obtained from mouse embryonic stem cells, *Saccharomyces cerevisiae*, and *Caenorhabditis elegans* (Beckmann et al., 2015; Kwon et al., 2013; Matia-González et al., 2015; Mitchell et al., 2013), confirming earlier findings and further uncovering the repertoire of RBPs.

Several of the unorthodox RBPs identified in these studies have been characterized for their physiological roles in RNA biology. These include metabolic enzymes (Beckmann et al., 2015), regulators of alternative splicing (Papasaikas et al., 2015; Tejedor et al., 2015), the E3 ubiquitin ligase TRIM25 (Choudhury et al., 2014), or the FAST kinase domain-containing protein 2 (FASTKD2) (Popow et al., 2015). However, the RNA-binding regions of these unorthodox RBPs remain largely unknown.

To identify the interaction sites of such proteins with RNA, UV crosslinking followed by extensive RNase treatment has been used to detect the peptide mass shift induced by the crosslinked RNA remnant via mass spectrometry (Schmidt et al., 2012). While conceptually simple, the mass heterogeneity of the nucleotide remnant has rendered this approach challenging in practice. Some RBDs have been characterized in vitro using this approach (reviewed in Schmidt et al., 2012), and a sophisticated algorithm allowed assignment of 257 binding sites from 124 proteins in yeast (Kramer et al., 2014). While informative, this data

 © 2016 The Author(s). Published by Elsevier Inc.
This is an open access article under the CC BY license (http://creativecommons.org/licenses/by/4.0/).

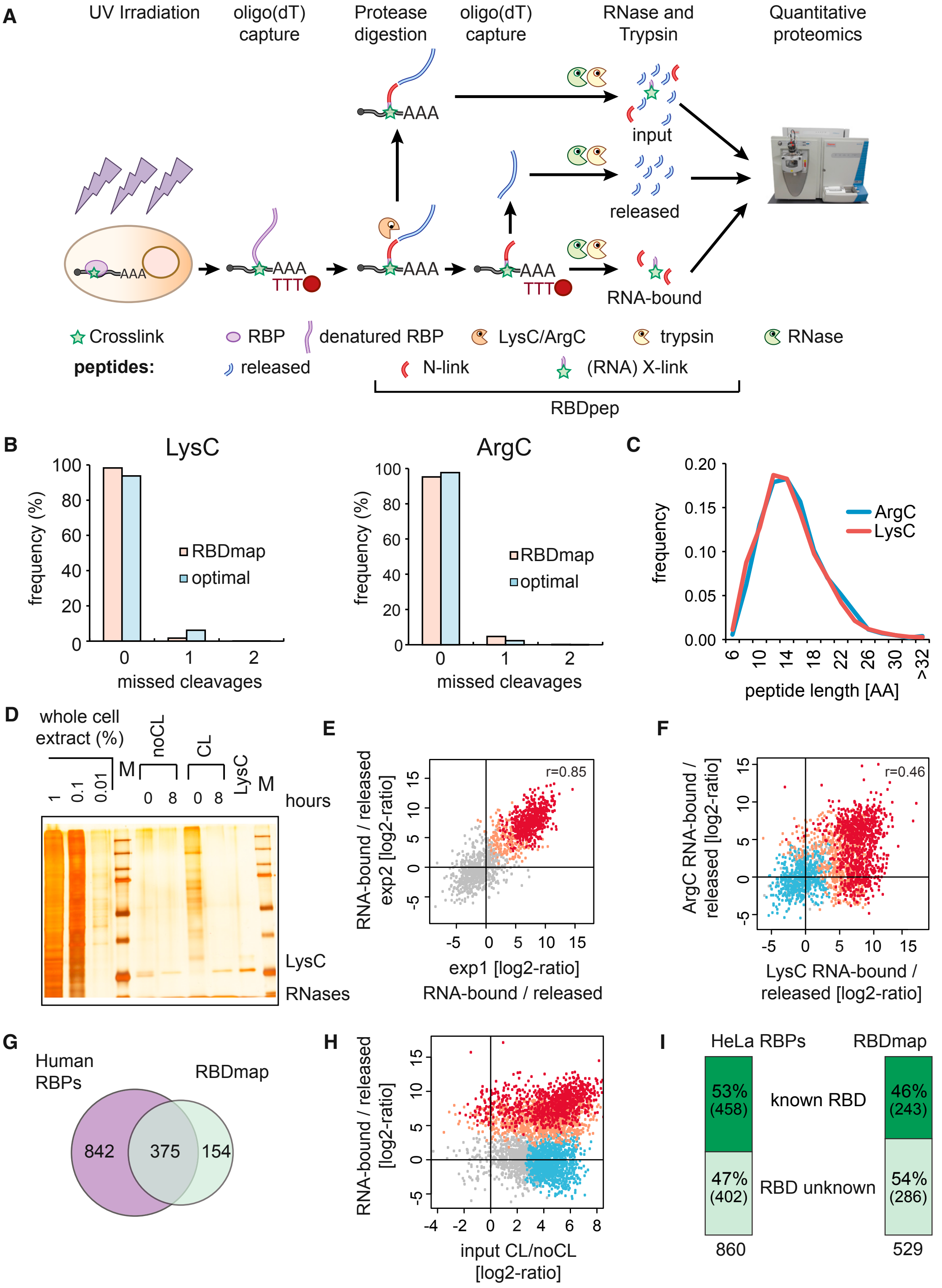

Figure 1. In Vivo Identification of RBDs by RBDmap

(A) Schematic representation of the RBDmap workflow.

(B) LysC- and ArgC-mediated proteolysis was monitored without trypsin treatment. The protease digestion under RBDmap conditions or in buffers typically used in MS studies (optimal) were compared to in silico digestions defining 0% miscleavage. The missed cleavages were calculated and plotted.

(C) Distribution of MS-identified LysC/ArgC fragments based on their number of amino acids.

(legend continued on next page)

set is strongly enriched for interactions mediated by RRMs, because the challenging identification of peptides with aberrant mass spectra requires both abundance and high crosslinking efficiency for detection. Nonetheless, 10% of the identified interaction sites mapped to non-canonical RBDs, supporting the existence of unanticipated modes of RNA binding.

Here, we develop and exploit RBDmap as a method for the in vivo identification of RBDs on a proteome-wide scale. We identified 1,174 high-confidence RNA-binding sites in 529 RBPs from HeLa cells, generating an unprecedented atlas of RNA-binding architectures in vivo.

RESULTS AND DISCUSSION

Proteome-wide Mapping of RBDs by RBDmap

To define how RBPs bind to RNA in living cells, we extended RNA interactome capture (Castello et al., 2013b) by addition of an analytical protease digestion step followed by a second round of oligo(dT) capture and mass spectrometry (Figure 1A). First, UV light is applied to cell monolayers to covalently stabilize native protein-RNA interactions taking place at "zero" distance (Pashev et al., 1991). While UV exposure using dosages exceeding those used here can potentially promote protein-protein crosslinking (Davidenko et al., 2016; Suchanek et al., 2005), we could not detect such crosslinks under our conditions, evidenced by the lack of UV-dependent, high molecular weight complexes in RNase-treated samples (Figures S1A and S4A; Strein et al., 2014).

Proteins crosslinked to poly(A) RNA are isolated using oligo(dT) magnetic beads and purified by stringent washes that include 500 mM LiCl and chaotropic detergents (0.5% LiDS), efficiently removing non-covalent binders (Castello et al., 2012, 2013b). After elution, RBPs are proteolytically digested by either LysC or ArgC. These proteases were selected as best suited for RBDmap by an in silico simulation of their predicted cleavage patterns of known HeLa RBPs (Castello et al., 2012) and their compatibility with subsequent tryptic digestion (Figure S1B). Analysis by mass spectrometry (MS) of LysC- and ArgC-treated samples revealed an excellent match with the in silico predictions, as reflected by the low number of missed cleavages (Figures 1B and 1C). The extensive proteolysis of HeLa RBPs is achieved without compromising RNA integrity (Figures 1D and S1C–S1E). The average peptide length after LysC and ArgC treatment is ∼17 amino acids, which defines the resolution of RBDmap (Figure 1C). Note that the extensive protease treatment disrupts protein integrity, and thus protein-protein complexes that might have withstood the experimental conditions will be released into the supernatant.

We collected an input sample aliquot after UV irradiation, oligo(dT) selection, and protease digestion, which in principle should reflect the RNA interactome (Figure 1A). When compared to a non-irradiated specificity control, the resulting high-confidence RBPs overlap 82% with the previously published human RNA interactomes (Baltz et al., 2012; Beckmann et al., 2015; Castello et al., 2012). This high concordance shows that LysC and ArgC treatments are fully compatible with the RNA interactome capture protocol. The remaining two thirds of the LysC or ArgC-treated samples were subjected to a second round of oligo(dT) purification leading to two peptide pools (Figure 1A): (1) peptides released from the RNA into the supernatant, and (2) peptides remaining covalently bound to the RNA, representing the RNA-binding sites of the respective RBPs. Importantly, subsequent tryptic digestion of the RNA-bound LysC/ArgC fragments yields two classes of peptides: the portion that still remains crosslinked to the RNA (X-link) and its neighboring peptides (N-link) (Figure 1A). While the directly crosslinked peptides (X-link) are difficult to identify due to the heterogeneous mass shift induced by the residual nucleotides (Kramer et al., 2014; Schmidt et al., 2012), the native peptides adjacent to the crosslinking site (N-link) can be identified by standard MS and peptide search algorithms. The original RNA-bound region of the RBP (i.e., RBDpep; Figure 1A), which includes both the crosslinked peptide (X-link) and its unmodified neighboring peptides (N-link), is then re-derived in silico by extending the MS-identified peptides to the two nearest LysC or ArgC cleavage sites.

Analysis of the RNA-bound and released fractions by quantitative proteomics shows high correlation of the resulting peptide intensity ratios between independent biological replicates. These ratios follow a bimodal distribution with one mode representing the released peptides (gray) and the other the RNA-bound ones (red; Figures 1E and S1F). We detected 909 and 471 unique N-link peptides as significantly enriched in the RNA-bound fractions of LysC- or ArgC samples, respectively (1% false discovery rate, FDR) (Figure S1G). Notably, computed RNA-bound/released peptide intensity ratios also correlate between the LysC and ArgC data sets (Figure 1F), supporting the robustness of the workflow. Due to their different specificities, each protease also contributes unique 1% FDR RBDpeps to the complete peptide superset (Figure S1G), covering 529 RBPs that highly overlap with human RNA interactomes (Figure 1G) (Baltz et al., 2012; Beckmann et al., 2015; Castello et al., 2012). Proteins within the RBDmap data set range from

(D) Silver staining shows the protein pattern of purified RBPs prior to and after LysC treatment (crosslinking: CL).
(E) Scatter plot comparing the peptide intensity ratios between RNA-bound and released fractions. The peptides enriched in the RNA-bound fraction at 1% (RBDpep) and 10% FDR (candidate RBDpep) are shown in red and salmon, respectively (Pearson correlation coefficient: r).
(F) Peptide intensity ratios between LysC and ArgC experiments computed from three biological replicates. The dots represent released peptides (blue), RBDpeps (red), candidate RBDpeps (salmon), and background peptides (gray).
(G) Venn diagram comparing the proteins within the RBDmap data set and the HeLa, HEK293, and Huh-7 RNA interactomes.
(H) Comparison of the peptide intensity ratios from three biological replicates between UV-irradiated and non-irradiated inputs (x axis) and between RNA-bound and released fractions (y axis) (color code as above).
(I) Number of proteins harboring recognizable or unknown RBDs in the HeLa mRNA interactome (left) and in RBDmap dataset (right).
See also Table S1 and Figure S1.

low to high abundance (Figure S1H), following a similar distribution as the input fraction and the HeLa RNA interactome (Castello et al., 2012). Thus, RBDmap is not selective for highly abundant proteins. There were 154 additional RBPs that were identified here, helped by the reduction of sample complexity and of experimental noise by the additional proteolytic step and the second oligo(dT) capture. In agreement with this explanation, the relative abundance of corresponding RBDpeps is higher in the RNA-bound fractions than in the "input" samples (Figures 1H and S1I). Thus, RBDmap detects RNA-binding regions within hundreds of RBPs in one approach, even if it does not cover all RBPs identified by RNA interactome capture (Figure 1G). Proteins will be missed by RBDmap when (1) binding to non-polyadenylated RNAs, (2) displaying low crosslinking efficiency, (3) interacting with the phospho-sugar backbone, but not the nucleotide bases, or (4) lacking suitable cleavage sites for trypsin within the LysC and ArgC proteolytic fragments and hence lacking MS-identifiable N-link peptides. Thus, the distribution of arginines (R) and lysines (K) will influence whether a given RBP can be studied by RBDmap, and we used two different proteases to maximize the identification of RBDpeps.

About half of the RBPs covered by RBDpeps harbor well-established RBDs and play known functions in RNA biology, reflected by a strong and significant enrichment of RNA-related protein domains and biological processes comparable to the HeLa RNA interactome (Figures 1I and S1J). Note that the reduced RBP coverage of RBDmap compared to RNA interactome capture equally affects both well-established and unorthodox RBPs (Figures 1I and S1J).

RBDmap "Rediscovers" Classic RBDs

Interestingly, RNA-bound and released proteolytic fragments display distinct chemical properties. Released peptides are rich in negatively charged and aliphatic residues, which are generally underrepresented in RNA-binding protein surfaces (Figures 2A, 2B, and S2A). Conversely, RBDpeps are significantly enriched in amino acids typically involved in protein-RNA interactions, including positively charged and aromatic residues. These data show that the chemical properties of the RBDpeps resemble those expected of bona fide RNA-binding surfaces. As a notable exception, glycine (G) is enriched in RBDpeps, but depleted from protein-RNA interfaces derived from available structures (Figures 2A and 2B). Flexible glycine tracks can contribute to RNA binding via shape-complementarity interactions as described for RGG boxes (Phan et al., 2011). Hence, lack of glycine at binding sites of protein-RNA co-structures reflects the technical limitations of crystallographic studies regarding disordered protein segments.

Validating the RBDmap data, classical RBDs such as RRM, KH, cold shock domain (CSD), and Zinc finger CCHC, are strongly enriched in the RNA-bound fraction (Figure 2C). This enrichment can also be appreciated at the level of individual protein maps (Figures 2D and S2B–S2D). To evaluate the capacity of RBDmap to identify bona fide RBDs, we focused on RBPs that harbor at least one classical RBD (as listed in Lunde et al., 2007). MS-identified peptides from these proteins were classified as "within" or "outside" a classical RBD, according to their position within the proteins' architecture (Figure 2E). The relative fraction of peptides within versus outside of the RBD was then plotted for each possible RNA-bound/released intensity ratio (Figure 2F). Correct re-identification of classical RBDs would lead to an ascending line (i.e., within/outside ratios should grow in parallel to the RNA-bound/release ratios; Figure 2E), while a random distribution of peptides within and outside of classical RBDs would yield a horizontal line (i.e., within/outside ratios do not vary in accordance with the RNA-bound/released ratios; Figure 2E). As shown in Figure 2F, the relative fraction of peptides mapping within classical RBDs increases in parallel with the RNA-bound/released ratios. Thus, RBDmap correctly assigns RNA-binding activity to well-established RBDs.

Unexpected initially, helicase domains are underrepresented in the RNA-bound fraction (Figure 2C). However, the high number of released helicase peptides likely reflects (1) the transitory and dynamic interactions that helicases establish with RNA, (2) the large protein segments of the domain situated far from the RNA, and (3) the predominance of interactions with the phospho-sugar backbone over nucleotide bases (Figures S2C–S2E) (Bono et al., 2006). Nevertheless, high-confidence RBDpeps are found at the exit of the helicase tunnel, as discussed below (Figures S2C–S2E).

High-Resolution Determination of RNA-Binding Sites

For direct validation of the RBDmap data, we selected all those RBPs for which protein-RNA co-structures are available within the Protein Data Bank (PDB) repository. These were "digested" in silico with either LysC or ArgC, and the predicted proteolytic fragments were considered as "proximal" to RNA when the distance to the closest RNA molecule is 4.3 Å or less; otherwise, they were categorized as non-proximal (Figure 3A). About half of all LysC and ArgC fragments are proximal to RNA by this criterion, reflecting that many RBP structures are incomplete and focused on the RBDs (average protein coverage ~50%). By contrast, 70.3% (LysC) and 81% (ArgC), respectively, of RBDpeps qualify as proximal, showing that RBPmap highly significantly enriches for peptides in close proximity to the RNA (Figure 3A). Several factors suggest that the pool of peptides classified as proximal in the analyzed structures even underestimates the performance of RBDmap: (1) in several structures of RBPs that harbor two or more RBDs, only one of the RBDs displays the interaction with RNA (e.g., PDB 3NNC) (Teplova et al., 2010). At least in some of these cases, structures lack RNA contacts of RBDs that likely occur in vivo. (2) Proteins are normally co-crystallized with short nucleic acids (5 to 8 nucleotides), and their physiological RNA partners likely establish additional interactions with the RBP. (3) RNA-protein co-structures usually reflect one interaction state, while protein-RNA interactions are typically more dynamic in vivo (Ozgur et al., 2015; Safaee et al., 2012).

RBDmap also correctly assigns RNA-binding regions within large protein complexes such as the nuclear cap-binding complex. The small nuclear cap-binding protein (NCBP) 2 (or CBP20) directly contacts mRNA via the cap structure (m7GpppG), while the larger NCBP1 (CBP80) interacts with NCBP2 (Mazza et al., 2002). In agreement, RBDmap defines the RNA-binding region of NCBP2 within the m7GpppG-binding pocket and no RBDpep is assigned to the large NCBP1

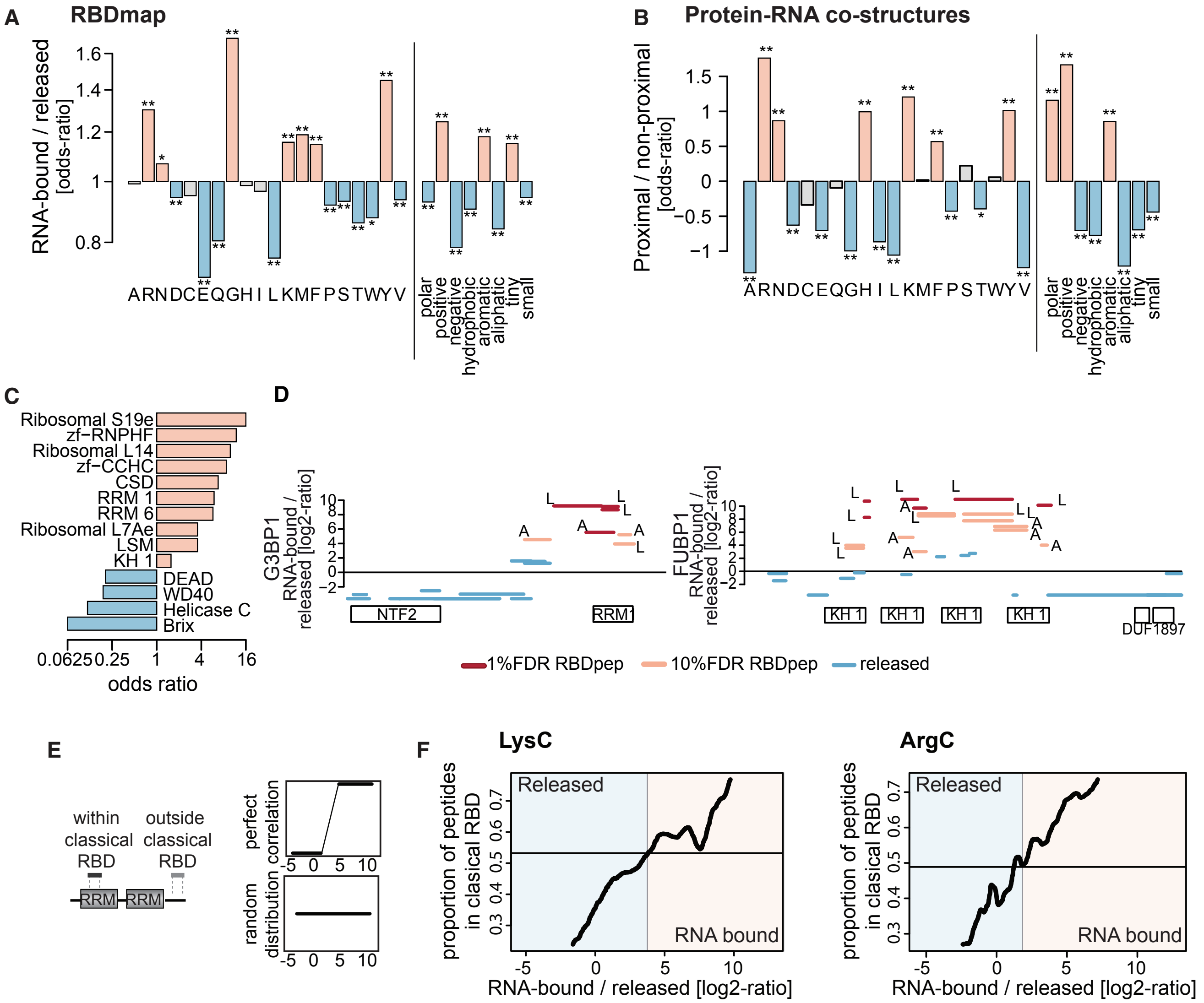

Figure 2. Identification of Well-Established RBDs by RBDmap

(A) Amino acid enrichment within RBDpeps (salmon) over released (blue) proteolytic fragments (*, 10% FDR and **, 1% FDR).

(B) Amino acid enrichment within RNA-binding protein surfaces (≤4.3 Å to the RNA) over distant regions (>4.3 Å from the RNA) extracted from protein-RNA co-structures.

(C) Bar plot showing the odds ratio of the most enriched known RBDs.

(D) Distribution of RBDpeps and released fragments in a classical RBP. The x axis represents the protein sequence from N to C terminus, and the y axis shows the RNA-bound/released peptide intensity ratios. The protein domains are shown in boxes under the x axis (LysC: L and ArgC: A).

(E) Schematic representation of RBDpeps mapping within or outside of classical RBDs (left). The idealized outcome of a perfect correlation between RBDpeps and classical RBDs (top right) and random distribution are shown (bottom right).

(F) Computed ratio of peptides mapping within known RBDs versus outside RBDs, regarding their peptide RNA-bound to released ratios. The horizontal line represents the baseline for uncorrelated data (i.e., the proportion of peptides mapping to classical RBD in the whole validation set in absence of enrichment; see E bottom).

See also Table S2 and Figure S2.

(Figure S3A). Moreover, RBDmap defines the corresponding RNA-binding sites within NCBP2 (Mazza et al., 2002) and its cytoplasmic counterpart eIF4E (Brown et al., 2007) (Figure S3B), in spite of their low sequence identity. The glutamyl-prolyl-tRNA synthetase (EPRS) represents a large non-canonical RBP that harbors two tRNA synthase domains separated by three WHEP motifs (Figures S3C and S3D). The first and second WHEP motif bind the GAIT RNA element present in the 3′ UTRs of a number of pro-inflammatory mRNAs (Jia et al., 2008), in complete agreement with the RBDmap data.

To test whether RNA-binding assignments of RBDmap can reach near single-amino acid resolution, we collected the complete set of RBDpeps and released peptides mapping to a given RBD class (e.g., RRM) and assessed their relative position within

CellPress

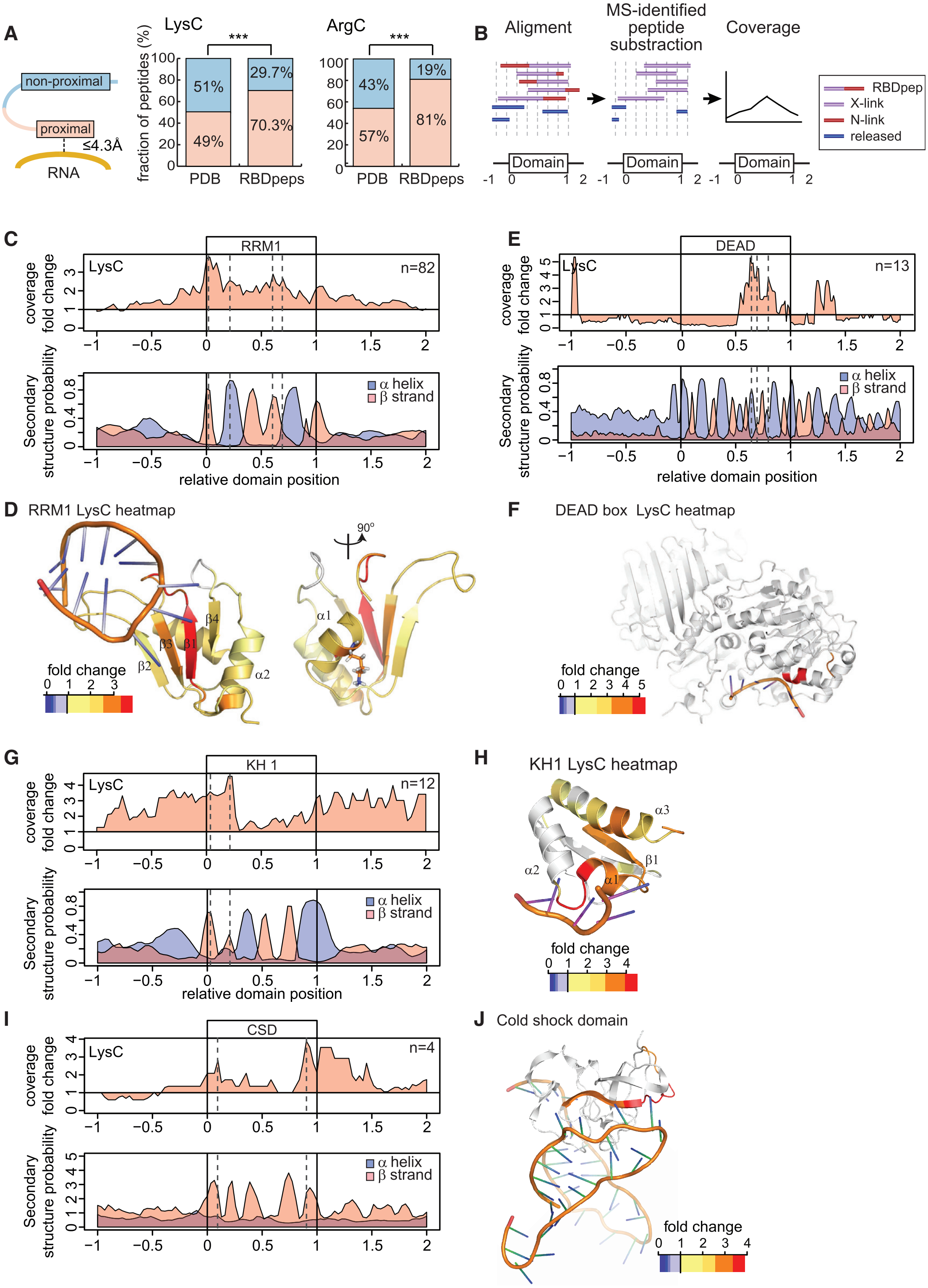

Figure 3. RBDmap Identifies RNA-Binding Regions with High Accuracy

(A) Schematic representation of proximal and non-proximal peptides (left). The proteins within protein-RNA co-structures were digested in silico with LysC or ArgC and predicted fragments aligned with the RBDpep supersets. The left bars represent the proportion of proximal and non-proximal LysC/ArgC fragments in the complete structure superset (random probability). The right bars show the % of aligned RBDpeps that are RNA proximal or non-proximal (***p < 0.001).

(legend continued on next page)

the domain (from 0 to 1) as well as its adjacent upstream (from −1 to 0) and downstream regions (from 1 to 2) (Figure 3B). The MS-identified part (N-link) of each RBDpep was then subtracted to infer the RNA-crosslinked (X-link) moiety(s), which cannot be identified by conventional MS due to their nucleotide remnant (Figures 1A and 3B). The X-link/released peptide ratio was calculated for each position in the domain, where high prevalence of X-link over released peptides will indicate RNA binding (Figure 3B). The high accuracy of this analysis is illustrated by the example profile obtained for RRMs. As shown in Figures 3C, 3D, and S3E, the highest X-link/released peptide ratio points to β strand 1, 2, and 3 as partners in the interaction with RNA, in agreement with the dozens of RNA-RRM co-structures available. Note that the LysC and ArgC proteases dissected the RRM in a differential manner: while LysC points to β strand 1 and 3, ArgC identifies β strand 2 as RNA-binding site, reflecting that the mapping capacity by these proteases depends on the distribution of lysines and arginines. Moreover, these data support the complementarity of the LysC and ArgC data sets to build accurate and comprehensive RNA-binding maps. Unexpectedly, we observed two discrete peaks of high X-link/released peptide ratio within the α helices placed at the back of the RRM. These peaks coincide with amino acids projected from the α helix to the RNA in several structures (Figure S3F) (Safaee et al., 2012; Teplova et al., 2010) and hence confirm the accuracy of RBDmap.

This analysis also successfully assigned correct RNA-binding sites to KH, DEAD-box helicase, and CSD, as shown in Figures 3E–3J, S3G, and S3H. The DEAD box helicase domain establishes interactions primarily with the phospho-sugar backbone of the RNA, while nucleotide bases project away from the protein core (Figure S3I). X-link peptide coverage of RBDmap for the DEAD box domain identifies one alpha helix in the helicase tunnel exit that coincides with the only position in RNA-protein co-crystals where multiple amino acids establish direct contacts with nucleotide bases. Interestingly, different binding orientations of the double-stranded RNA-binding motif (DSRM) have been observed in structural studies (Figure S3J) (Fu and Yuan, 2013; Ramos et al., 2000). The X-link peptide coverage analysis of the DSRM domain highlights the loop separating the second and third β strands as interaction partners with the double-stranded RNA (Figures S3J and S3K). Note that this loop is shown in several RNA-protein co-structures to be projected into the minor grove of the double-stranded RNA helix, establishing numerous interactions with the Watson-Crick paired bases (Lunde et al., 2007). In summary, RBDmap faithfully re-identifies the protein surfaces of canonical RBDs that contact nucleotide bases.

Identification of Non-canonical RBDs

For more than half of the RBPs characterized by RBDmap, no functional or domain annotation related to RNA biology is currently available (Figures 1I and S1J). RBDpeps identify dozens of unorthodox globular RBDs associated with different molecular functions, including DNA binding, enzymatic cores, mediators of protein-protein interactions, or of protein localization (Figure 4A; Table S2). As an illustrative example, thioredoxin (TXN) catalyzes disulfide bond formation and has recently been discovered in RNA interactomes (Beckmann et al., 2015; Castello et al., 2012). RBDmap identifies an RBDpep at the N terminus of TXN (Figure 4B; Table S1) that overlaps with two solvent-exposed lysines (K3 and 8) highlighted as potential binding sites in the X-link coverage analysis for the TXN fold (Figures 4B and 4C). To evaluate this assignment functionally, we expressed TXN-eGFP fusion proteins in HeLa cells. Following in vivo UV crosslinking, oligo(dT) capture, and stringent washes, green fluorescence in eluates was measured to quantify RNA binding (Figure 4D) (Castello et al., 2013b; Strein et al., 2014). We used unfused eGFP as negative control and the well-established RNA-binding helicase MOV10 as a positive control for RNA binding (Gregersen et al., 2014). Although all the fusion proteins are expressed at similar levels in cells, only TXN-eGFP and MOV10-YFP co-purify with poly(A) RNAs significantly above background (Figure 4E). Mutation of K3 and/or K8 to glutamic acid (E) totally abrogates TXN RNA-binding activity. Conversely, conservative mutation to arginine (R) is tolerated. These results experimentally validate the accurate identification of a previously unknown RNA-binding region by RBDmap.

We also noticed clusters of RBDpeps within enzymes. Peptidyl prolyl *cis/trans* isomerases are classified based on their domain architecture into two groups: PPI and FKBP. This protein superfamily has close links to RNA metabolism, and two members, PPIE and PPIL4, harbor classical RRMs (Mesa et al., 2008). However, RNA interactome studies found 11 additional members of this family that lack RRMs as RBPs, suggesting the existence of a still unknown mechanism of RNA binding (Castello et al., 2012). RBDmap reveals this RNA-binding activity within both the PPI and FKBP folds (Tables S1 and S2). Although lacking sufficient peptide coverage to perform an X-link peptide analysis, we noticed two clusters of RBDpeps at the N- and C-termini of the FKBP fold that are located far apart in primary sequence, but close

(B) Schematic representation of the X-link peptide coverage analysis.
(C) x axis represents the relative position of the RRM (from 0 to 1) and their upstream (−1 to 0) and downstream (1 to 2) regions. The ratio of the X-link over released peptides at each position of the RRM and surrounding regions using the LysC data set was plotted (top). The secondary structure prediction for each position of the RRM and flanking regions is shown (bottom).
(D) The ratio of X-link over released peptides was plotted in a representative RRM-RNA structural model (PDB 2FY1) using a heatmap color code.
(E) As in (C), but for the DEAD-box domain.
(F) As in (D), but using the PDB 2J0S as a DEAD-box helicase model.
(G) As in (C), but for the KH domain.
(H) As in (D), but using the PDB 4B8T as a model for a KH domain.
(I) As in (C), but for the CSD.
(J) As in (D), but with the PDB 3TS2 as a model for a CSD.
See also Table S2 and Figure S3.

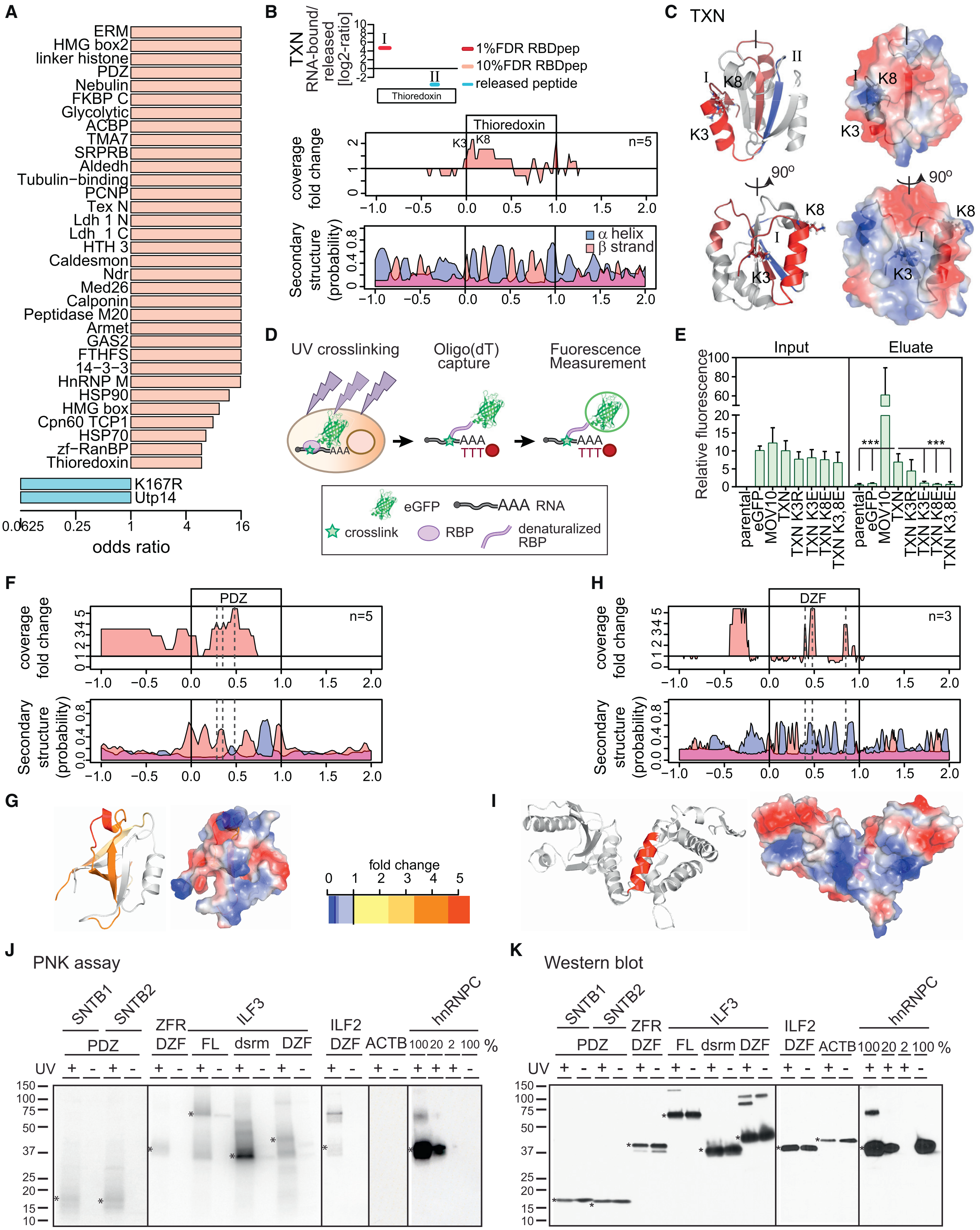

(legend on next page)

in 3D structure (Figures S4B and S4C). The mapped candidate RBD opposes the catalytic site.

Furthermore, we noticed clusters of RBDpeps in six chaperones of the heat shock protein (HSP) 90 and 70 families (Figure S4D). HSPs are induced by cellular stress and prevent protein misfolding and subsequent aggregation, which typically occur in disordered regions of RBPs in health and disease (Weber and Brangwynne, 2012). Indeed, HSPs have been functionally linked to RNA metabolism and translation (Iwasaki et al., 2010; Willmund et al., 2013). Chaperone domain binding to RNA may help to increase the local concentration of the chaperone machinery at ribonucleoprotein complexes to avoid the accumulation of pathological aggregates.

Apparently, numerous enzymes of intermediary metabolism bind RNA through regions in close proximity to their substrate-binding pockets. Specifically, the di-nucleotide binding domain (or Rossmann fold) and mono-nucleotide binding folds emerge as bona fide RBDs with 12 proteins mapped by RBDmap (Table S3), extending earlier observations (Cieśla, 2006; Nagy and Rigby, 1995). RBDpeps mapping to Aldolase (ALDO) A and C delimit the fructose 1,6 bisphosphate interacting domain (Figures S4E and S4F), suggesting that RNA and metabolite may compete for this binding pocket. Overall, the RBDpeps identified within metabolic enzymes show that the few well-characterized examples such as aconitase 1 (iron regulatory protein 1, IRP1), glyceraldehyde-3-phophate dehydrogenase, and thymidylate synthase may represent the tip of the iceberg of a more general engagement of metabolic enzymes with RNA (reviewed in Castello et al., 2015).

RBDmap also uncovers RNA-binding activities within PDZ, 14-3-3, ERM, and the tubulin-binding domains, which are involved in protein-protein interactions and protein localization (Figures 4F, 4G, and S4G–S4I). Due to the high peptide coverage of the PDZ domain, we could generate an X-link analysis (Figures 4F and 4G). This map shows a discrete RNA-binding site within a basic cavity formed by a short α helix and two β strands.

RBDmap also identifies RNA-binding sites within domains of unknown function such as NDR and DZF. N-myc downstream-regulated genes (NDRGs) represent a family of proteins with unknown function. NDRG1 is a metastasis suppressor relevant for cancer progression and prognosis (Chang et al., 2014), its exact molecular function has remained unknown. RBDmap resolves a conserved RNA-binding region within the NDR domain of NDRG1, NDRG2, and NDRG4. RBDpeps reproducibly map to the helix-loop-β strand structure at the C terminus of the NDR fold (Figures S4J and S4K). DZF is predicted to harbor nucleotidyltransferase activity (Kuchta et al., 2009) and to promote protein dimerization (Wolkowicz and Cook, 2012). The X-link peptide coverage analysis maps the RNA-binding region to a deep, basic cleft between two symmetrical domain subunits (Figures 4H and 4I). The RNA-binding activity of the DZF domain is compatible with its proposed nucleotidyltransferase function.

To independently assess RNA-binding of PDZ and DZF domains, we used the T4 polynucleotide kinase (PNK) assay as an orthogonal approach. In brief, cells are irradiated with UV light and, after lysis, RNA is trimmed with RNase I. Proteins of interest are immunoprecipitated under stringent conditions and the presence of RNA revealed by 5′ end phosphorylation with PNK and [γ-^{32}P]-ATP, followed by SDS-PAGE and autoradiography. We generated Tet-inducible HeLa cell lines expressing the PDZ domain of β-1-syntrophin (SNTB) 1 and SNTB2, as well as the DZF domains of Zinc finger RNA-binding protein (ZFR) and interleukin enhancer-binding factor (ILF) 2 and ILF3, all fused to a FLAG-HA tag. As positive controls, we used the full-length ILF3 (FL), its DSRM domain alone, and hnRNPC, while actin (ACTB) was used as a negative control. The PNK assay shows radioactive bands of the expected molecular weight for all tagged PDZ and DFZ domains and only when UV light was applied to the cultured cells (Figures 4J and 4K). By contrast, no signal is detectable for the control ACTB. As expected, the DSRM domain of ILF3 also displays RNA-binding activity. Taken together, these data corroborate the RBDmap assignment of PDZ and DZF domains as RBDs.

Even if functional studies will have to define the physiological roles of these unconventional RBDs in the future, their biological relevance warrants consideration. It is possible that these RBDs may endow RBPs with "moonlighting" activities in posttranscriptional regulation, akin to cytosolic aconitase (IRP1) (Muckenthaler et al., 2008). Alternatively, the RBDs could serve as "docking sites" for regulatory or scaffolding RNAs that inhibit, activate, or modify protein functions. In analogy, innate immune effectors such as PKR, TLR3, TLR7, TLR8, or RIG-I, can be controlled by pathogen-derived RNAs (Barbalat et al., 2011; Yu and Levine, 2011). RNA may also serve to recruit proteins to RNPs, akin to NEAT1 RNA in paraspeckle formation (Clemson et al., 2009). The identification of these RBDs and the mapping of the RNA-interaction sites for hundreds of proteins serve as a

Figure 4. Globular RBDs Discovered by RBDmap

(A) Odds ratios for the most highly enriched RBDs.

(B) RBDpep and released peptides mapping to TXN as in Figure 2D (top). The ratio of the X-link over released peptide coverage at each position of the TXN fold as in Figure 3C is shown (middle). The secondary structure prediction for each position of the TXN fold and flanking regions is shown (bottom).

(C) Crystal structure of human TXN (PDB 3M9J), K3 and K8 are highlighted, and the identified RBDpep is shown in red.

(D) Schematic representation of the protocol for measurement of RNA-binding using eGFP fusion proteins.

(E) Relative total (input) or RNA-bound (eluate) green fluorescence signal from cells expressing different eGFP fusion proteins (***$p < 0.01$, t test, and n = 9).

(F) As in (B), but for PDZ domain.

(G) Ratio of X-link over released peptides plotted as a heatmap in a PDZ homology model.

(H) As in (B), but for DZF domain.

(I) As in (G), but using a DZF homology model.

(J) Autoradiography of FLAG-HA tagged proteins after PNK assay.

(K) Western blotting using an antibody against the HA tag. The polypeptides of the expected molecular masses are indicated by asterisks.

See also Tables S2, S3, and S5 and Figure S4.

critical step toward definition of the biological functions of these RBPs in detail.

Disordered Regions Emerge as Frequent RNA Interaction Sites In Vivo

A high proportion of the human RBPs lack native 3D structure (Castello et al., 2012), and these disordered regions can occasionally engage in non-canonical protein-RNA interactions (45 examples reviewed in Järvelin et al., 2016). In some instances, these interactions can induce co-folding of both molecules (Phan et al., 2011). While this mode of interaction emerged recently, the scope of disordered motifs involved in RNA-binding remained unknown. Strikingly, half of the RBDpeps map to disordered regions, and RBDmap identifies a disordered RBD as the sole detectable RNA-binding site for 170 RBPs (Figures 5A 5B,, and S5A). Disordered RBDpeps largely mirror the chemical properties of the whole RBDpep superset, apart from the expected enrichment for disorder-promoting residues (proline [P], serine [S], and glycine [G]), as well as R and glutamine (Q) (Figures 5C and S5B).

Detailed analysis identifies clusters of disordered RBDpeps that can be classified on the basis of sequence motifs. While a few R-rich, RGG, and SR repeats have previously been shown to bind RNA experimentally (Järvelin et al., 2016), RBDmap expands the RNA-binding role of these motifs by dozens of additional examples (Figures 5D and S5C). The superset of RNA-binding RGG boxes can be subclassified by the lengths of the glycine linkers (Thandapani et al., 2013). Because glycines can position arginines and contribute to RNA binding providing shape complementarity, G-linker length could serve in setting the motif's specificity for RNA. In agreement, both arginine and glycine substitutions impair RGG-RNA recognition (Phan et al., 2011).

Aromatic residues are typically found in hydrophobic cores. However, histidines (H), phenylalanines (F), and especially tyrosines (Y) occur within the RNA-binding disordered regions (Figures 5D and S5C). YGG repeats (also called [G/S]Y[G/S]) can promote protein aggregation in vitro, inducing hydrogel formation and amyloid-like fibers, as well as dynamic phase transitions in vivo (Han et al., 2012; Kato et al., 2012). Since YGG repeats are identified as a potential RNA-binding motif in our data set, it will be important to elucidate whether their RNA-binding capacity is affected by the aggregation state and, conversely, whether RNA-binding to such disordered linear motifs can affect phase transitions and granule formation (Zhang et al., 2015).

Lysine (K) combines with negatively charged residues, G, P, or Q, to form distinctive RNA-binding motifs (Figures 5D and S5C). The stoichiometry and distances between lysines and other amino acids are similar across analogous K-rich motifs present in non-homologous proteins (Figure 5E). Several copies of a repeat combining basic and acidic residues within the neuroblast differentiation-associated protein AHNAK are identified by RBDmap (Figure S5D), suggesting that low complexity regions can contribute to modular RNA-binding architectures, similar to globular RBDs. Interestingly, the K-rich regions within RBPs display similarities with the basic tails of DNA-binding proteins. The large capture radius of these disordered regions play important roles in transcription factor activity by favoring "hopping" and "sliding" over 3D diffusion to reach their target sequences (Vuzman et al., 2010). K-rich sequences may play similar roles in RBPs.

To validate the disordered regions identified by RBDmap as bona fide RNA-binding motifs, we fused the RGG-rich and the K-rich sequences from FUS and Methyl-CpG-binding protein 2 (MECP2), respectively, to eGFP and tested the fusion proteins with the same assay as in Figure 4D: both short motifs suffice to confer RNA-binding to eGFP (Figures 5F and 5G).

The biological function and mode of interaction of disordered regions with RNA should be further investigated.

Uncovering Biological Properties of RBDs

Previously unknown RNA-binding globular and disordered regions display similar mean isoelectric points as known RBDs (Figure 6A), while their released counterparts exhibit a significantly lower isoelectric point, as expected. Thus, (1) both previously unknown and well-characterized RBDs share common chemical properties, (2) they differ from released fragments, and (3) the unorthodox RBDs do not artificially associate with RNA due to an abnormally high isoelectric point. Established RBPs and proteins harboring previously unknown globular and disordered RBDs display very similar mRNA abundance profiles, ranging from low to high levels, with a slight tendency to lower abundance for the unconventional folded and disordered RNA-binding regions (Figures 6B and 6C). Thus, proteins with unorthodox RBDs are not biased toward high abundance. Notably, RBDpeps in both globular and disordered RBDs are more highly conserved throughout evolution than their released counterparts (Figure 6D), suggesting functional relevance.

Cross-referencing of the RBDpep data sets with databases of curated posttranslational modifications shows that RNA-binding sites represent hot spots for defined post-translational modifications (PTMs, $p = 2.025 \times 10^{-08}$), including tyrosine phosphorylation, methylation, acetylation, and malonylation (Figure 6E). This finding suggests that, reminiscent of chromatin remodeling, RBDs are posttranslationally regulated and respond to signaling and metabolic cues. The conserved amino acid contexts of these PTMs implicate sequence-selective modifying enzymes (Figure 6F). Interestingly, acetylation frequently occurs in a lysine two positions upstream of a conserved proline (Figure 6F). Proline isomerization in the basic tail of histone H3 is regulated by acetylation of adjacent lysines and has notable consequences for protein conformation (Howe et al., 2014). Our results suggest the possibility that this regulatory mechanism could also apply to RBP regulation.

Our data also show that Mendelian disease mutations cluster within RBDs compared to natural variants ($p = 0.0001796$) (Figure 6G; Table S4). For example, one RBDpep maps to an RGG-box in FUS that is a hotspot for disease-associated mutations (Figure 6H) (Shang and Huang, 2016), and the RNA-binding activity of this region is validated here by an orthogonal approach (Figures 4D and 5G). Interestingly, a mutation in this region (R495X) causes amyotrophic lateral sclerosis (ALS) and correlates with impaired interaction of FUS with the SMN complex and reduced localization to nuclear gems (Yamazaki et al., 2012). The relationship between altered RNA-binding

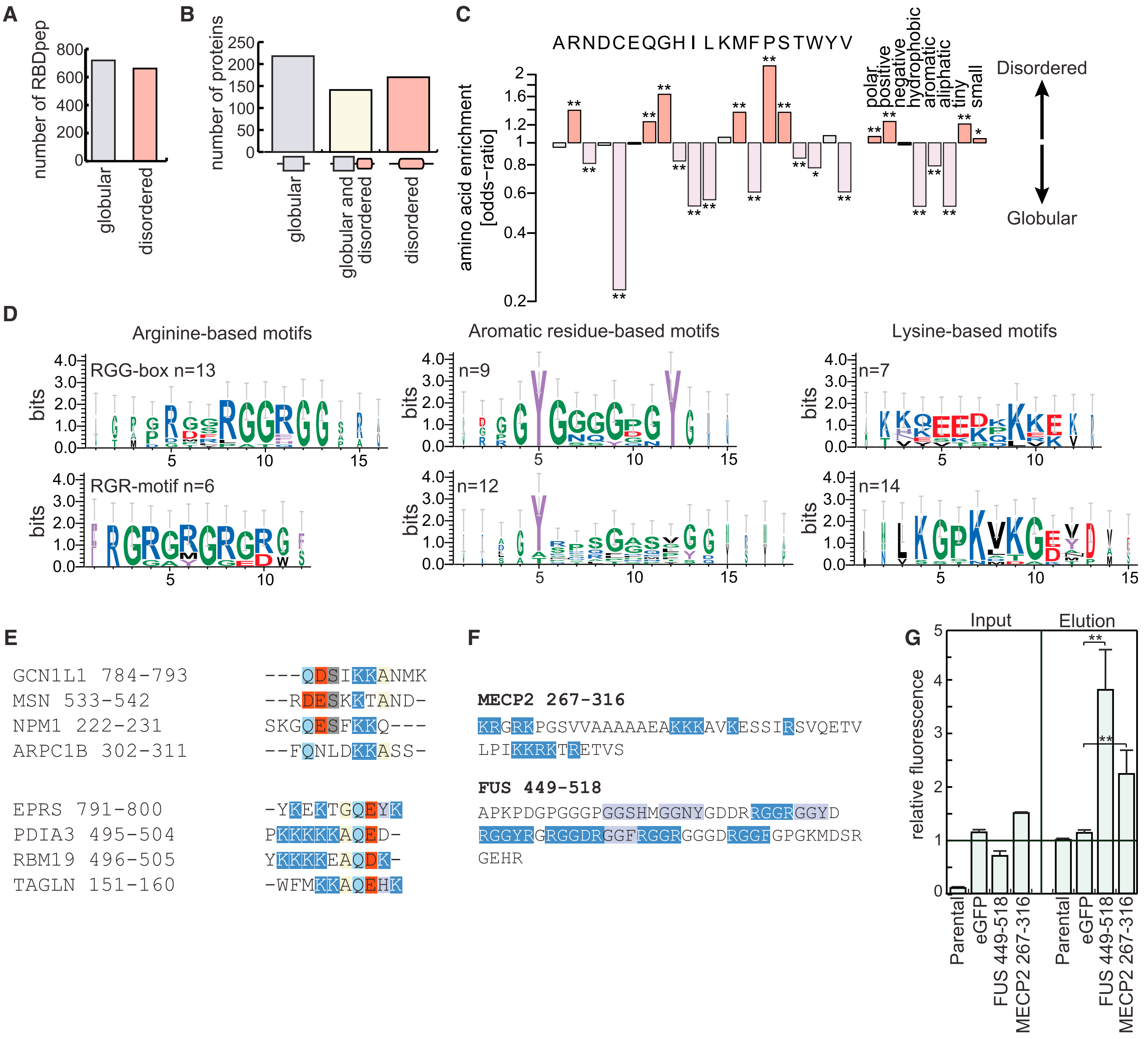

Figure 5. Disordered Protein Regions as RBDs

(A) Number of RBDpeps mapping to globular and disordered domains.

(B) Number of proteins mapped by at least one RBDpep solely in globular domains, in globular and disordered domains, or only in disordered motifs.

(C) Amino acid enrichment between globular (violet) and disordered (pink) RBDs (*, 10% FDR and **, 1% FDR).

(D) Multiple sequence alignment of short, disordered RBDpeps with clustal omega. The sequence logos were extracted from aligned disordered fragments.

(E) Examples of alignment of K-rich protein motifs.

(F) Disordered RNA-binding motifs from FUS and MECP2 expressed as eGFP fusion.

(G) Relative total (input) or RNA-bound (eluate) green fluorescence signal from cells expressing $FUS_{449–518}$-eGFP, $MECP2_{267–316}$-eGFP, or unfused eGFP as a negative control (**p < 0.01, t test, and n = 6).

See also Figure S5.

and disease phenotypes in this and other proteins deserves further exploration.

Conclusions

RBDmap provides an unprecedented identification of RNA-binding regions of RBPs in living cells. It describes 1,174 high confidence (1% FDR) RNA-binding sites within 529 proteins. These sites have been validated as a whole by stringent statistical analyses (Figure 1) and cross-correlation with well-established RBPs and domains, previously studied by biochemical and structural means (Figures 2 and 3). We also validated a small number of previously unknown RBDs (TXN, PDZ, DZF, and the

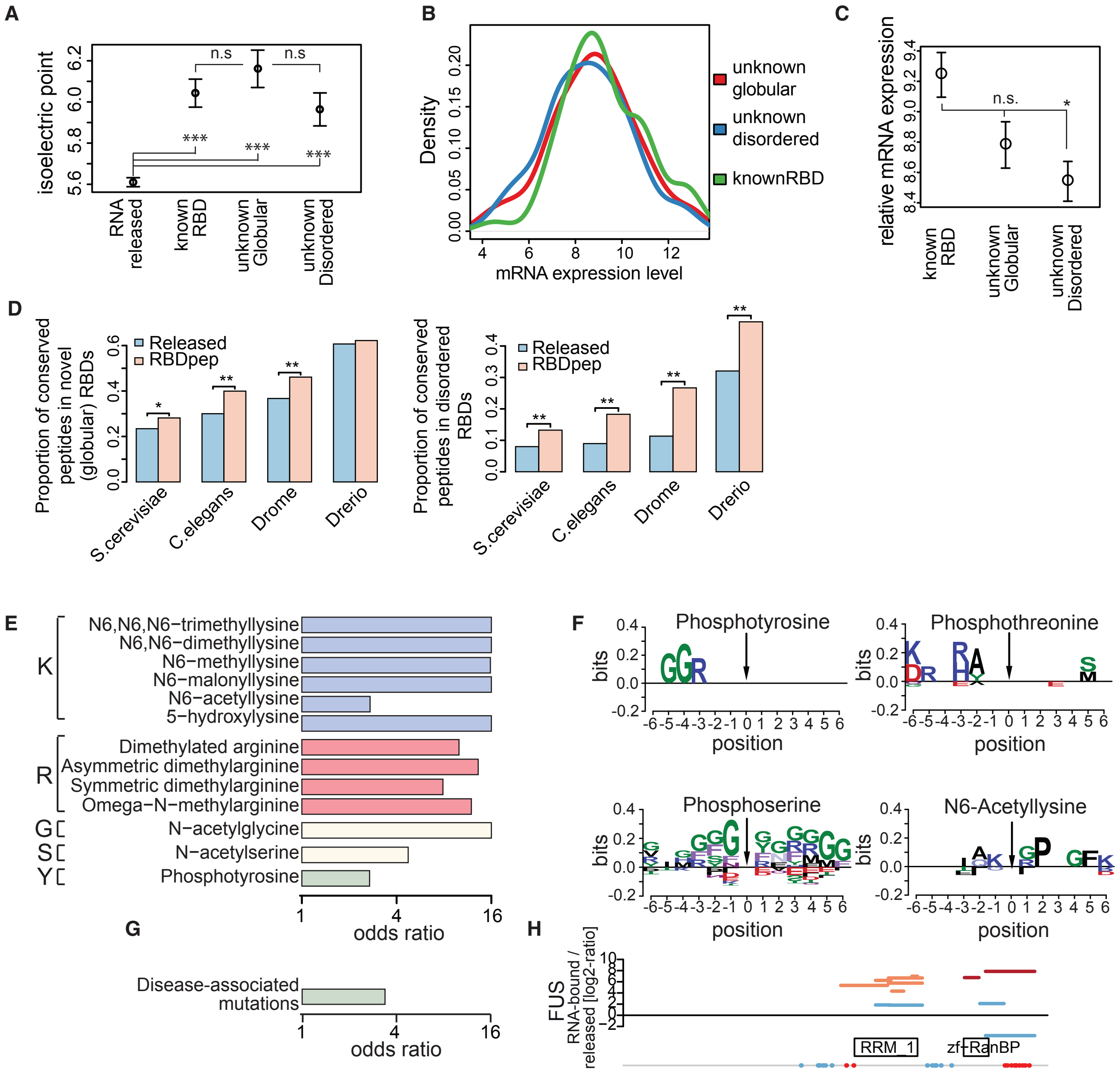

Figure 6. Features of Known and Previously Unknown RBDs

(A) Dots show the mean isoelectric point of all LysC and ArgC fragments (the bars represent SEM) (***$p < 0.01$ and not statistically significant: n.s.).

(B) Density plot comparing mRNA abundances of known RBPs and previously unknown globular and disordered RBPs.

(C) Dots show the mean of the mRNA abundance of the protein groups described in (B) (*$p < 0.05$ and not statistically significant: n.s.).

(D) Bar plot showing the conservation of RBDpeps and released fragments using *Homo sapiens* as reference (*$p < 0.05$ and **$p < 0.01$).

(E) Odds ratios for the most enriched PTMs in RBDpeps versus released fragments.

(F) Sequence logos of conserved amino acids around posttranslational modifications. A position weight matrix is computed from all 12-mer sequences around the modified residue (10% FDR amino acids are shown).

(G) Bar plot showing the odds ratio of Mendelian mutations occurring in RNA-bound over released fragments.

(H) RBDmap of FUS. The position of the disease-associated mutations is represented as red or blue colored circles if mapping within or outside an RBDpep, respectively.

See also Table S4.

disordered regions of MECP2 and FUS) individually, applying orthogonal methods (Figures 4 and 5). Against this background, we recommend similar validation experiments for any individual RBD of interest before further in depth analyses.

Our data suggest that multifunctional globular domains, which combine RNA-binding with enzymatic functions or protein-protein interaction surfaces, are commonplace, not rare exceptions. These invoke additional functions for RNA, including the (allosteric or competitive) control of catalytic activities and of protein-protein interactions. Moreover, disordered regions are found to play common roles in native protein-RNA interactions, comprising half of the total RNA-binding sites identified.

The RNA-binding motifs identified here share physico-chemical features of well-established RBDs, are conserved across evolution, and represent hot spots for posttranslational modifications and disease-associated mutations. Individually and in combination, these features suggest important biological roles.

As a method, RBDmap can now be applied to other cell types and organisms such as *S. cerevisiae*, *Caenorhabditis elegans*, or *Drosophila melanogaster* to study the evolution of RBDs. It can also be applied to cells subjected to different experimental conditions to investigate the responses of RBPs to physiological cues such as e.g., stress, starvation, or differentiation.

EXPERIMENTAL PROCEDURES

RBDmap

Initial UV crosslinking and oligo(dT) purification followed the mRNA interactome capture protocol (Castello et al., 2013b). Complete proteolytic digestions were performed with LysC or ArgC for 8 hr at 37°C. Polyadenylated RNA and crosslinked peptides were diluted in 20 mM Tris-HCl, 500 mM LiCl, 1 mM DTT, and 0.5 mM EDTA and recaptured on oligo(dT) beads. The supernatant was processed for MS (released peptides). oligo(dT) beads were washed as in Castello et al. (2013b). All fractions were treated with trypsin and labeled with stable isotopes in vitro (Boersema et al., 2008). Peptides were analyzed on a liquid chromatography-tandem MS (LC-MS/MS) platform. The R-scripts used for the analyses can be found in the R/Bioconductor data-package RBDmapHeLa (http://www.bioconductor.org). RBDmap data can be accessed under http://www-huber.embl.de/users/befische/RBDmap.

MS, Protein Identification, and Quantification

Proteins were processed following standard protocols, and the resulting peptides were labeled with stable isotopes in vitro, fractionated, and analyzed on a nano-HPLC system (Proxeon) or nano-Acquity UPLC system (Waters) coupled directly to an LTQ Orbitrap Velos (Thermo Fisher Scientific).

Data Analysis

A complete description of data analysis can be found in the Supplemental Information.

Fluorescence-Based Method to Measure RNA-Binding In Vivo and PNK Assay

Tet-on HeLa cells expressing eGFP fusion proteins were generated as described elsewhere (Castello et al., 2012). Upon induction, cells were UV irradiated and subjected to small scale RNA interactome capture (Castello et al., 2013b). Eluates were measured in a plate reader. For PNK assays, cell monolayers were irradiated with 150 mJ/cm2 UV_{254} (Castello et al., 2013b). After cell lysis and RNase treatment, FLAG-HA tagged proteins were immunoprecipitated with an anti-FLAG antibody coupled to magnetic beads (M8823, Sigma Aldrich) and processed as in Beckmann et al. (2015). More detailed information can be found in the Supplemental Information.

ACCESSION NUMBERS

The accession number for the proteomics data reported in this paper is ProteomeXchange Consortium (http://www.proteomexchange.org): PXD000883.

SUPPLEMENTAL INFORMATION

Supplemental Information includes Supplemental Experimental Procedures, five figures, and five tables and can be found with this article online at http://dx.doi.org/10.1016/j.molcel.2016.06.029.

AUTHOR CONTRIBUTIONS

A.C., B.F., and M.W.H. contributed to the conception and design of the project. A.C., R.H., and A.-M.A. carried out the experimental work. C.K.F., S.F., and J.K. performed the proteomic analyses. B.F., T.C., A.C., C.K.F., J.K., and M.W.H. performed the data analyses. A.C. and M.W.H. wrote the manuscript with input from all authors.

ACKNOWLEDGMENTS

We thank Drs. Benedikt Beckmann and the M.W.H. group for helpful discussions. A.C. is funded by MRC Career Development Award #MR/L019434/1. M.W.H. acknowledges support by ERC Advanced Grant ERC-2011-ADG_20110310 and the Virtual Liver Network of the German Ministry for Science and Education. C.K.F. is supported by EMBO postdoctoral fellowship LTF1006-2013.

Received: September 17, 2015
Revised: May 31, 2016
Accepted: June 20, 2016
Published: July 21, 2016

REFERENCES

Baltz, A.G., Munschauer, M., Schwanhäusser, B., Vasile, A., Murakawa, Y., Schueler, M., Youngs, N., Penfold-Brown, D., Drew, K., Milek, M., et al. (2012). The mRNA-bound proteome and its global occupancy profile on protein-coding transcripts. Mol. Cell *46*, 674–690.

Barbalat, R., Ewald, S.E., Mouchess, M.L., and Barton, G.M. (2011). Nucleic acid recognition by the innate immune system. Annu. Rev. Immunol. *29*, 185–214.

Beckmann, B.M., Horos, R., Fischer, B., Castello, A., Eichelbaum, K., Alleaume, A.M., Schwarzl, T., Curk, T., Foehr, S., Huber, W., et al. (2015). The RNA-binding proteomes from yeast to man harbour conserved enigmRBPs. Nat. Commun. *6*, 10127.

Boersema, P.J., Aye, T.T., van Veen, T.A., Heck, A.J., and Mohammed, S. (2008). Triplex protein quantification based on stable isotope labeling by peptide dimethylation applied to cell and tissue lysates. Proteomics *8*, 4624–4632.

Bono, F., Ebert, J., Lorentzen, E., and Conti, E. (2006). The crystal structure of the exon junction complex reveals how it maintains a stable grip on mRNA. Cell *126*, 713–725.

Brown, C.J., McNae, I., Fischer, P.M., and Walkinshaw, M.D. (2007). Crystallographic and mass spectrometric characterisation of eIF4E with N7-alkylated cap derivatives. J. Mol. Biol. *372*, 7–15.

Butter, F., Scheibe, M., Mörl, M., and Mann, M. (2009). Unbiased RNA-protein interaction screen by quantitative proteomics. Proc. Natl. Acad. Sci. USA *106*, 10626–10631.

Castello, A., Fischer, B., Eichelbaum, K., Horos, R., Beckmann, B.M., Strein, C., Davey, N.E., Humphreys, D.T., Preiss, T., Steinmetz, L.M., et al. (2012). Insights into RNA biology from an atlas of mammalian mRNA-binding proteins. Cell *149*, 1393–1406.

Castello, A., Fischer, B., Hentze, M.W., and Preiss, T. (2013a). RNA-binding proteins in Mendelian disease. Trends Genet. *29*, 318–327.

Castello, A., Horos, R., Strein, C., Fischer, B., Eichelbaum, K., Steinmetz, L.M., Krijgsveld, J., and Hentze, M.W. (2013b). System-wide identification of RNA-binding proteins by interactome capture. Nat. Protoc. *8*, 491–500.

Castello, A., Hentze, M.W., and Preiss, T. (2015). Metabolic enzymes enjoying new partnerships as RNA-binding proteins. Trends Endocrinol. Metab. *26*, 746–757.

Chang, X., Xu, X., Ma, J., Xue, X., Li, Z., Deng, P., Zhang, S., Zhi, Y., Chen, J., and Dai, D. (2014). NDRG1 expression is related to the progression and prognosis of gastric cancer patients through modulating proliferation, invasion and cell cycle of gastric cancer cells. Mol. Biol. Rep. *41*, 6215–6223.

Choudhury, N.R., Nowak, J.S., Zuo, J., Rappsilber, J., Spoel, S.H., and Michlewski, G. (2014). Trim25 is an RNA-specific activator of Lin28a/TuT4-mediated uridylation. Cell Rep. *9*, 1265–1272.

Cieśla, J. (2006). Metabolic enzymes that bind RNA: yet another level of cellular regulatory network? Acta Biochim. Pol. *53*, 11–32.

Clemson, C.M., Hutchinson, J.N., Sara, S.A., Ensminger, A.W., Fox, A.H., Chess, A., and Lawrence, J.B. (2009). An architectural role for a nuclear noncoding RNA: NEAT1 RNA is essential for the structure of paraspeckles. Mol. Cell *33*, 717–726.

Davidenko, N., Bax, D.V., Schuster, C.F., Farndale, R.W., Hamaia, S.W., Best, S.M., and Cameron, R.E. (2016). Optimisation of UV irradiation as a binding site conserving method for crosslinking collagen-based scaffolds. J. Mater. Sci. Mater. Med. *27*, 14.

Fu, Q., and Yuan, Y.A. (2013). Structural insights into RISC assembly facilitated by dsRNA-binding domains of human RNA helicase A (DHX9). Nucleic Acids Res. *41*, 3457–3470.

Glisovic, T., Bachorik, J.L., Yong, J., and Dreyfuss, G. (2008). RNA-binding proteins and post-transcriptional gene regulation. FEBS Lett. *582*, 1977–1986.

Gregersen, L.H., Schueler, M., Munschauer, M., Mastrobuoni, G., Chen, W., Kempa, S., Dieterich, C., and Landthaler, M. (2014). MOV10 Is a 5′ to 3′ RNA helicase contributing to UPF1 mRNA target degradation by translocation along 3′ UTRs. Mol. Cell *54*, 573–585.

Han, T.W., Kato, M., Xie, S., Wu, L.C., Mirzaei, H., Pei, J., Chen, M., Xie, Y., Allen, J., Xiao, G., and McKnight, S.L. (2012). Cell-free formation of RNA granules: bound RNAs identify features and components of cellular assemblies. Cell *149*, 768–779.

Howe, F.S., Boubriak, I., Sale, M.J., Nair, A., Clynes, D., Grijzenhout, A., Murray, S.C., Woloszczuk, R., and Mellor, J. (2014). Lysine acetylation controls local protein conformation by influencing proline isomerization. Mol. Cell *55*, 733–744.

Iwasaki, S., Kobayashi, M., Yoda, M., Sakaguchi, Y., Katsuma, S., Suzuki, T., and Tomari, Y. (2010). Hsc70/Hsp90 chaperone machinery mediates ATP-dependent RISC loading of small RNA duplexes. Mol. Cell *39*, 292–299.

Järvelin, A.I., Noerenberg, M., Davis, I., and Castello, A. (2016). The new (dis)order in RNA regulation. Cell Commun. Signal. *14*, 9.

Jia, J., Arif, A., Ray, P.S., and Fox, P.L. (2008). WHEP domains direct noncanonical function of glutamyl-Prolyl tRNA synthetase in translational control of gene expression. Mol. Cell *29*, 679–690.

Kato, M., Han, T.W., Xie, S., Shi, K., Du, X., Wu, L.C., Mirzaei, H., Goldsmith, E.J., Longgood, J., Pei, J., et al. (2012). Cell-free formation of RNA granules: low complexity sequence domains form dynamic fibers within hydrogels. Cell *149*, 753–767.

Kramer, K., Sachsenberg, T., Beckmann, B.M., Qamar, S., Boon, K.L., Hentze, M.W., Kohlbacher, O., and Urlaub, H. (2014). Photo-cross-linking and high-resolution mass spectrometry for assignment of RNA-binding sites in RNA-binding proteins. Nat. Methods *11*, 1064–1070.

Kuchta, K., Knizewski, L., Wyrwicz, L.S., Rychlewski, L., and Ginalski, K. (2009). Comprehensive classification of nucleotidyltransferase fold proteins: identification of novel families and their representatives in human. Nucleic Acids Res. *37*, 7701–7714.

Kwon, S.C., Yi, H., Eichelbaum, K., Föhr, S., Fischer, B., You, K.T., Castello, A., Krijgsveld, J., Hentze, M.W., and Kim, V.N. (2013). The RNA-binding protein repertoire of embryonic stem cells. Nat. Struct. Mol. Biol. *20*, 1122–1130.

Lukong, K.E., Chang, K.W., Khandjian, E.W., and Richard, S. (2008). RNA-binding proteins in human genetic disease. Trends Genet. *24*, 416–425.

Lunde, B.M., Moore, C., and Varani, G. (2007). RNA-binding proteins: modular design for efficient function. Nat. Rev. Mol. Cell Biol. *8*, 479–490.

Matia-González, A.M., Laing, E.E., and Gerber, A.P. (2015). Conserved mRNA-binding proteomes in eukaryotic organisms. Nat. Struct. Mol. Biol. *22*, 1027–1033.

Mazza, C., Segref, A., Mattaj, I.W., and Cusack, S. (2002). Large-scale induced fit recognition of an m(7)GpppG cap analogue by the human nuclear cap-binding complex. EMBO J. *21*, 5548–5557.

Mesa, A., Somarelli, J.A., and Herrera, R.J. (2008). Spliceosomal immunophilins. FEBS Lett. *582*, 2345–2351.

Mitchell, S.F., Jain, S., She, M., and Parker, R. (2013). Global analysis of yeast mRNPs. Nat. Struct. Mol. Biol. *20*, 127–133.

Muckenthaler, M.U., Galy, B., and Hentze, M.W. (2008). Systemic iron homeostasis and the iron-responsive element/iron-regulatory protein (IRE/IRP) regulatory network. Annu. Rev. Nutr. *28*, 197–213.

Nagy, E., and Rigby, W.F. (1995). Glyceraldehyde-3-phosphate dehydrogenase selectively binds AU-rich RNA in the NAD(+)-binding region (Rossmann fold). J. Biol. Chem. *270*, 2755–2763.

Ozgur, S., Buchwald, G., Falk, S., Chakrabarti, S., Prabu, J.R., and Conti, E. (2015). The conformational plasticity of eukaryotic RNA-dependent ATPases. FEBS J. *282*, 850–863.

Papasaikas, P., Tejedor, J.R., Vigevani, L., and Valcárcel, J. (2015). Functional splicing network reveals extensive regulatory potential of the core spliceosomal machinery. Mol. Cell *57*, 7–22.

Pashev, I.G., Dimitrov, S.I., and Angelov, D. (1991). Crosslinking proteins to nucleic acids by ultraviolet laser irradiation. Trends Biochem. Sci. *16*, 323–326.

Phan, A.T., Kuryavyi, V., Darnell, J.C., Serganov, A., Majumdar, A., Ilin, S., Raslin, T., Polonskaia, A., Chen, C., Clain, D., et al. (2011). Structure-function studies of FMRP RGG peptide recognition of an RNA duplex-quadruplex junction. Nat. Struct. Mol. Biol. *18*, 796–804.

Popow, J., Alleaume, A.M., Curk, T., Schwarzl, T., Sauer, S., and Hentze, M.W. (2015). FASTKD2 is an RNA-binding protein required for mitochondrial RNA processing and translation. RNA *21*, 1873–1884.

Ramos, A., Grünert, S., Adams, J., Micklem, D.R., Proctor, M.R., Freund, S., Bycroft, M., St Johnston, D., and Varani, G. (2000). RNA recognition by a Staufen double-stranded RNA-binding domain. EMBO J. *19*, 997–1009.

Safaee, N., Kozlov, G., Noronha, A.M., Xie, J., Wilds, C.J., and Gehring, K. (2012). Interdomain allostery promotes assembly of the poly(A) mRNA complex with PABP and eIF4G. Mol. Cell *48*, 375–386.

Scherrer, T., Mittal, N., Janga, S.C., and Gerber, A.P. (2010). A screen for RNA-binding proteins in yeast indicates dual functions for many enzymes. PLoS ONE *5*, e15499.

Schmidt, C., Kramer, K., and Urlaub, H. (2012). Investigation of protein-RNA interactions by mass spectrometry–techniques and applications. J. Proteomics *75*, 3478–3494.

Shang, Y., and Huang, E.J. (2016). Mechanisms of FUS mutations in familial amyotrophic lateral sclerosis. Brain Res., S0006-8993(16)30165-2.

Strein, C., Alleaume, A.M., Rothbauer, U., Hentze, M.W., and Castello, A. (2014). A versatile assay for RNA-binding proteins in living cells. RNA *20*, 721–731.

Suchanek, M., Radzikowska, A., and Thiele, C. (2005). Photo-leucine and photo-methionine allow identification of protein-protein interactions in living cells. Nat. Methods *2*, 261–267.

Tejedor, J.R., Papasaikas, P., and Valcárcel, J. (2015). Genome-wide identification of Fas/CD95 alternative splicing regulators reveals links with iron homeostasis. Mol. Cell *57*, 23–38.

Teplova, M., Song, J., Gaw, H.Y., Teplov, A., and Patel, D.J. (2010). Structural insights into RNA recognition by the alternate-splicing regulator CUG-binding protein 1. Structure *18*, 1364–1377.

Thandapani, P., O'Connor, T.R., Bailey, T.L., and Richard, S. (2013). Defining the RGG/RG motif. Mol. Cell *50*, 613–623.

Tsvetanova, N.G., Klass, D.M., Salzman, J., and Brown, P.O. (2010). Proteome-wide search reveals unexpected RNA-binding proteins in *Saccharomyces cerevisiae*. PLoS One *5*, e12671.

Vuzman, D., Azia, A., and Levy, Y. (2010). Searching DNA via a "Monkey Bar" mechanism: the significance of disordered tails. J. Mol. Biol. *396*, 674–684.

Weber, S.C., and Brangwynne, C.P. (2012). Getting RNA and protein in phase. Cell *149*, 1188–1191.

Willmund, F., del Alamo, M., Pechmann, S., Chen, T., Albanèse, V., Dammer, E.B., Peng, J., and Frydman, J. (2013). The cotranslational function of ribosome-associated Hsp70 in eukaryotic protein homeostasis. Cell *152*, 196–209.

Wolkowicz, U.M., and Cook, A.G. (2012). NF45 dimerizes with NF90, Zfr and SPNR via a conserved domain that has a nucleotidyltransferase fold. Nucleic Acids Res. *40*, 9356–9368.

Yamazaki, T., Chen, S., Yu, Y., Yan, B., Haertlein, T.C., Carrasco, M.A., Tapia, J.C., Zhai, B., Das, R., Lalancette-Hebert, M., et al. (2012). FUS-SMN protein interactions link the motor neuron diseases ALS and SMA. Cell Rep. *2*, 799–806.

Yu, M., and Levine, S.J. (2011). Toll-like receptor, RIG-I-like receptors and the NLRP3 inflammasome: key modulators of innate immune responses to double-stranded RNA viruses. Cytokine Growth Factor Rev. *22*, 63–72.

Zhang, H., Elbaum-Garfinkle, S., Langdon, E.M., Taylor, N., Occhipinti, P., Bridges, A.A., Brangwynne, C.P., and Gladfelter, A.S. (2015). RNA controls polyQ protein phase transitions. Mol. Cell *60*, 220–230.

Molecular Cell, Volume *63*

Supplemental Information

Comprehensive Identification

of RNA-Binding Domains in Human Cells

Alfredo Castello, Bernd Fischer, Christian K. Frese, Rastislav Horos, Anne-Marie Alleaume, Sophia Foehr, Tomaz Curk, Jeroen Krijgsveld, and Matthias W. Hentze

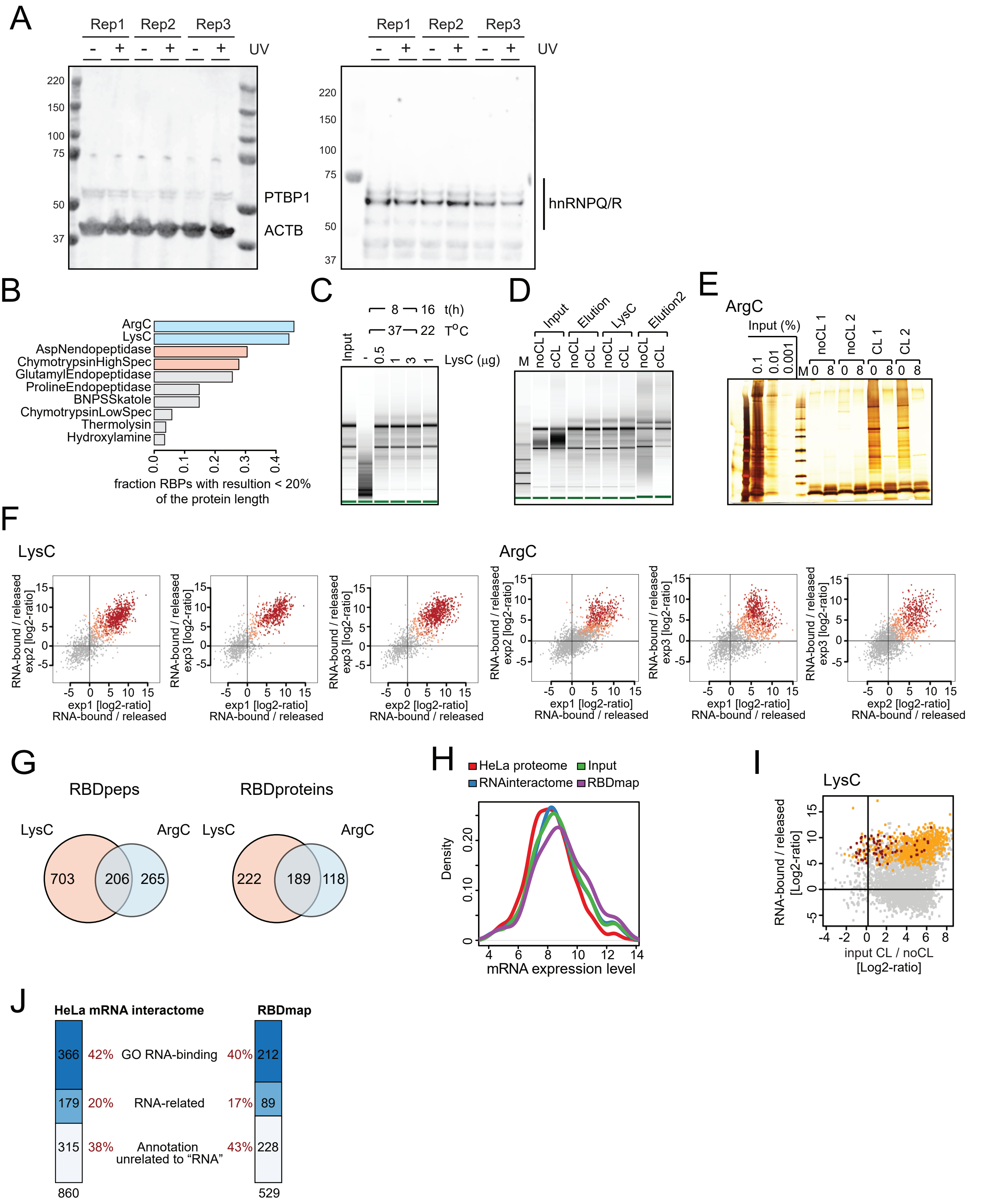
A
Rep1 Rep2 Rep3
- + - + - + UV
220 150 100 75 50 37
PTBP1
ACTB
220 150 100 75 50 37
hnRNPQ/R
B
ArgC
LysC
AspNendopeptidase
ChymotrypsinHighSpec
GlutamylEndopeptidase
ProlineEndopeptidase
BNPSSkatole
ChymotrypsinLowSpec
Thermolysin
Hydroxylamine
0.0 0.1 0.2 0.3 0.4
fraction RBPs with resultion < 20% of the protein length
C
8 16 t(h)
37 22 T°C
Input - 0.5 1 3 1 LysC (µg)
D
M noCL cCL noCL cCL noCL cCL noCL cCL
Input Elution LysC Elution2
E
ArgC
Input (%)
0.1 0.01 0.001 M
noCL 1 noCL 2 CL 1 CL 2
0 8 0 8 0 8 0 8
F
LysC
ArgC
RNA-bound / released exp2 [log2-ratio]
RNA-bound / released exp3 [log2-ratio]
exp1 [log2-ratio] RNA-bound / released
exp2 [log2-ratio] RNA-bound / released
-5 0 5 10 15
G
RBDpeps
RBDproteins
LysC ArgC
703 206 265
222 189 118
H
HeLa proteome Input
RNAinteractome RBDmap
Density
0 0.10 0.20
4 6 8 10 12 14
mRNA expression level
I
LysC
RNA-bound / released [Log2-ratio]
-5 0 5 10 15
-4 -2 0 2 4 6 8
input CL / noCL [Log2-ratio]
J
HeLa mRNA interactome
RBDmap
366 42% GO RNA-binding 40% 212
179 20% RNA-related 17% 89
315 38% Annotation unrelated to "RNA" 43% 228
860
529

Figure S1

Figure S1. Identification of RBDs by RBDmap. Related to Figure 1 and Table S1.
A) Western blot against ACTB, PTBP1 and hnRNPQ/R using whole cell lysates of UV_{254} irradiated and non-irradiated HeLa cells from three independent biological replicates. B) Computational simulation of protease efficiencies in RBDmap experiments. The RBPs of the HeLa mRNA interactome (Castello et al., 2012) were digested *in silico* using the different proteases available for MS experiments. The peptides identified in (Castello et al., 2012) were used as a proxy for protein coverage of an RBDmap experiment performed with the same cell line. We then selected the peptides that do not span the cleavage sites predicted for each protease and assumed the existence of the putative RNA-binding site at the centre of each RBP to calculate the best theoretical RBD resolution associated with each protease. The fractural number of proteins mapped for which the RBD was resolved to at least 20% of the actual protein length is represented. C) RNA integrity analysis under different LysC digestion conditions of oligo(dT)-purified samples (input). Samples were treated with proteinase K and monitored by bioanalyser. D) RNA analysis using bioanalyser of a representative LysC RBDmap experiment. E) Protein quality control of two independent experiments using ArgC. Poly(A) RNA extracted from UV irradiated (CL) and non-irradiated (noCL) cells was purified by oligo(dT) selection. Co-purified proteins were treated with 1µg of ArgC and analysed by silver staining prior to and after protease digestion. Optimization of LysC digestion of UV-irradiated oligo(dT) purified samples (input) applying different protease concentrations, incubation times and temperatures. F) Scatter plots comparing the peptide intensity ratios between RNA-bound and released fractions of three independent LysC and ArgC experiments. The peptides enriched in the RNA-bound over the released fraction at 1% and 10% FDR, respectively, are shown in red and salmon. G) Venn diagram comparing LysC and ArgC datasets at the peptide or protein level at 1% FDR. H) Density of mRNA levels of the whole HeLa proteome (red), the HeLa RNA interactome (Castello et al., 2012) (blue), the input sample (i.e. equivalent to the HeLa mRNA interactome - green), and proteins assigned with at least one 1% FDR RNA-binding site by RBDmap (purple). I) Scatter plot comparing the average peptide intensity ratios from three biological replicates between UV irradiated and non-irradiated samples (X axis) and between RNA-bound and released fractions (Y axis). Red represents RBDpeps (1% FDR) belonging to newly discovered proteins, while yellow peptides represent the rest of RBDpeps. J) Number of proteins annotated with the GO term RNA-binding, with a GO term related to RNA, or with an annotation unrelated to RNA in the HeLa mRNA interactome (left) and in RBDmap datasets (right).

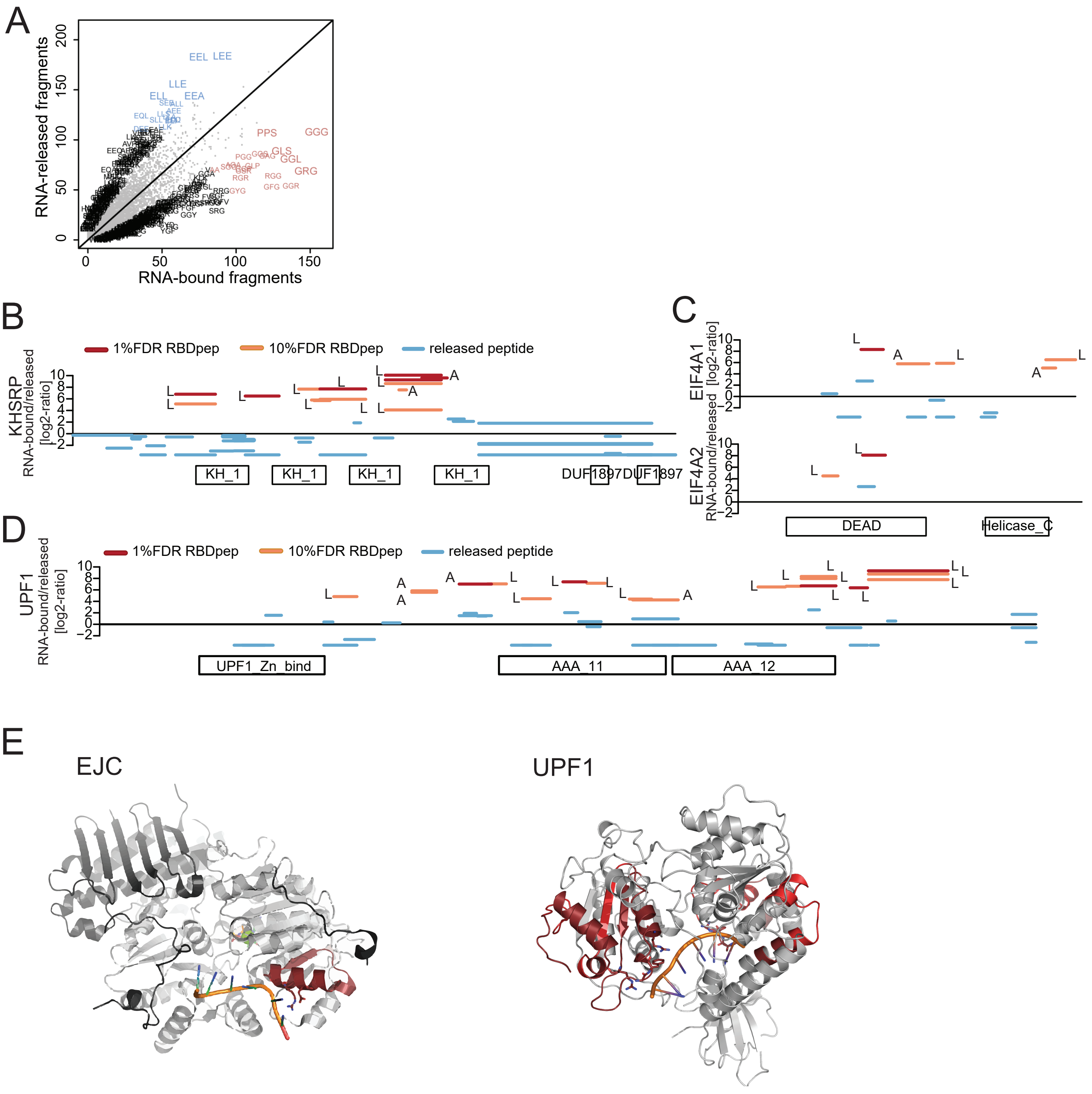

Figure S2. Benchmarking RBDmap. Related to Figure 2 and Table S2.

A) Enrichment of peptide trimers in RNA-bound (X axis) and released (Y axis) proteolytic fragments. In salmon and blue are the most abundant trimers in RNA-bound or released fractions. B-D) LysC and ArgC proteolytic fragment distribution of an illustrative KH-domain (B), DEAD box- (C) or AAA_11/AAA_12- (D) containing RBP. X axes represent proteins from N- to C-termini, while the Y axes show the RNA-bound/released peptide intensity ratios. Positions of the protein domains are shown in boxes under the X axis. E) The RBDpep (red) conserved between EIF4A1 and EIF4A2 was placed in the structure of their homolog EIF4A3 (light grey), which was crystalized in a complex with MAGOH, Y14 and barentz (dark grey) forming the exon junction complex (EJC, PDB 2j0s) (Bono et al., 2006). This region is highly conserved between the three homologs (EIF4A3 LDYGQ-HVVAGTPGRVFD-MIRRRSLRTR; EIF4A1, LQMEAPHIIVGTPGRVFDMLNRRYLSPK EIF4A2 LQAEAPHIVVGTPGRVFDMLNRRYLSPK) and is placed at the exit of the RNA tunnel (left panel). Right panel shows the RBDpeps (red) within UPF1, projected in the crystal structure of UPF1 with RNA (PDB 2xzo) (Chakrabarti et al., 2011).

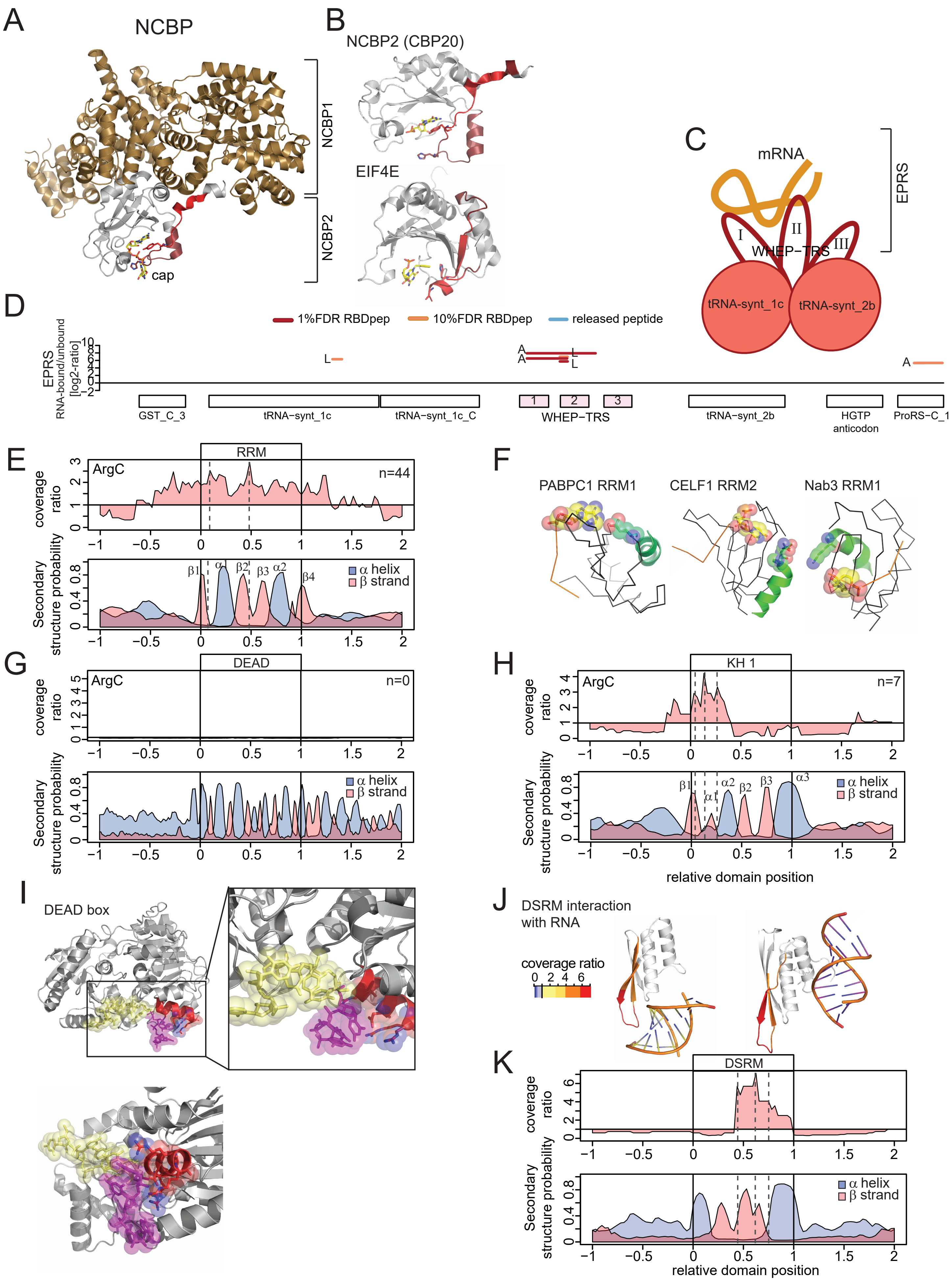
A
NCBP
NCBP1
NCBP2
cap
B
NCBP2 (CBP20)
EIF4E
C
mRNA
EPRS
I
II
III
WHEP-TRS
tRNA-synt_1c
tRNA-synt_2b
D
1%FDR RBDpep
10%FDR RBDpep
released peptide
EPRS
RNA-bound/unbound [log2-ratio]
GST_C_3
tRNA-synt_1c
tRNA-synt_1c_C
WHEP-TRS
tRNA-synt_2b
HGTP anticodon
ProRS-C_1
E
RRM
ArgC
n=44
coverage ratio
Secondary structure probability
α helix
β strand
F
PABPC1 RRM1
CELF1 RRM2
Nab3 RRM1
G
DEAD
ArgC
n=0
H
KH 1
ArgC
n=7
relative domain position
I
DEAD box
J
DSRM interaction with RNA
coverage ratio
K
DSRM
relative domain position

Figure S3

Figure S3. RBDmap identifies well-established RNA-binding surfaces in known RBPs with high accuracy. Related to Figure 3 and S2.

A) Crystal structure of the nuclear cap-binding complex bound to the cap structure (PDB 1h2t) (Mazza et al., 2001). NCBP2 is depicted in grey and NCBP1 in gold. RBDpeps are shown in red. B) Location of the RBDpep in NCBP2 (PDB 1h2t) (Mazza et al., 2001) and its cytoplasmic homolog EIF4E (PDB 2v8x). C) Schematic representation of the reported interaction mechanism of EPRS with mRNAv (Jia et al., 2008). D) The RBDpep distribution of the EPRS protein matches the biochemical and functional data reported in (Arif et al., 2009; Jia et al., 2008; Mukhopadhyay et al., 2008). E) X axis represents the relative position of the RRM (from 0 to 1) and their upstream (-1 to 0) and downstream (1 to 2) regions. The ratio of the X-link over released peptides at each position of the RRM and surrounding regions using the ArgC dataset was computed and plotted (top). Secondary structure prediction for each position of the RRM and flanking regions (bottom). F) Crystal structures showing the interaction of amino acids in the α-helices of the RRM with the RNA (PDBs 4f02, 3nnc, 2l41). These structures agree with the LysC X-link coverage analysis in Figure 3C. G) As in (E) but for DEAD box domain. H) As in (E) but for KH1. I) Detail of eIF4A3 (DEAD-box) interacting with RNA (PDB 2j0s). RNA is shown in pale yellow, except for the ribonucleotides that are contacted by amino acids projected from the DEAD-box domain, which are shown in magenta. The protein region enriched in the X-link peptide coverage analysis is shown in red. J) The ratio of X-link over released peptides was plotted for two structures in which the DSRM domain is bound to double stranded RNA in different orientations (PDBs 3vyx, 3adl) using a heat map color code. K) As in (E) but for DSRM.

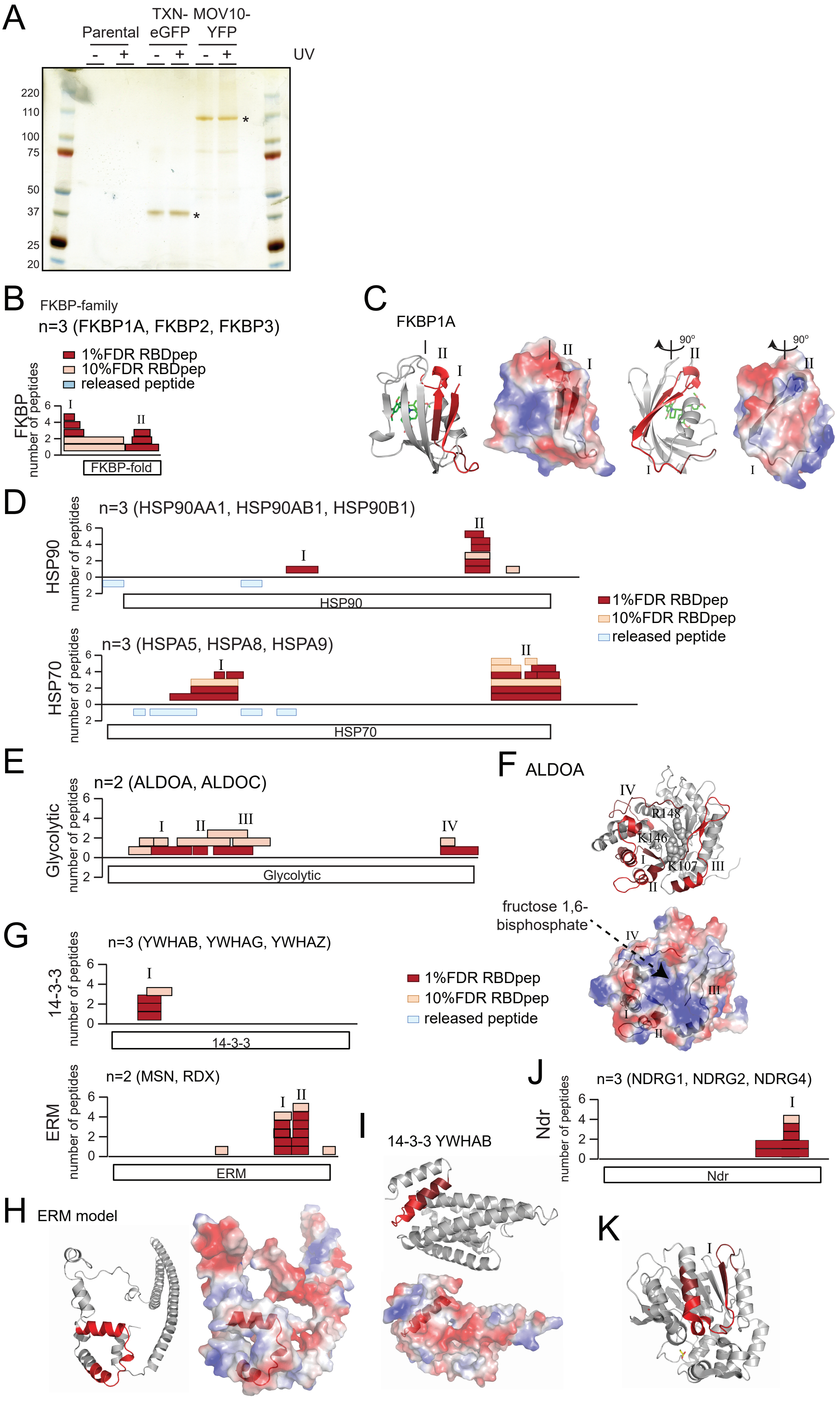
A
Parental
TXN-eGFP
MOV10-YFP
UV
B
FKBP-family
n=3 (FKBP1A, FKBP2, FKBP3)
1%FDR RBDpep
10%FDR RBDpep
released peptide
FKBP
number of peptides
FKBP-fold
C
FKBP1A
D
n=3 (HSP90AA1, HSP90AB1, HSP90B1)
HSP90
n=3 (HSPA5, HSPA8, HSPA9)
HSP70
E
n=2 (ALDOA, ALDOC)
Glycolytic
F
ALDOA
fructose 1,6-bisphosphate
R148
K146
K107
G
n=3 (YWHAB, YWHAG, YWHAZ)
14-3-3
n=2 (MSN, RDX)
ERM
H
ERM model
I
14-3-3 YWHAB
J
n=3 (NDRG1, NDRG2, NDRG4)
Ndr
K

Figure S4

Figure S4. Novel globular RBDs. Related to Figure 4, Table S2 and S3.
A) HeLa Flip-In Trex (parental), TXN-eGFP and MOV10-YFP were induced overnight with tetracycline. Cells were UV-irradiated or with 254 nm UV light or left untreated. Lysates from these cells were used for immunoprecipitation of GFP/YFP fusion proteins with GFP_Trap_A, and eluates were analyzed by silver staining. B) RBDpep distribution across all the FKBP protein family members characterized by RBDmap (FKBP1A, FKBP2, FKBP3). C) Crystal structure of FKBP1 bound to a synthetic ligand (PDB 1bl4). The electrostatic potential of the protein surface is shown in blue for basic and red for acidic surfaces. D) As in (B) but for HSP90 (top) and HSP70 (bottom) protein family members. E) As in (B), but for aldolase A and C. F) Ribbon diagram of ALDOA (top), where amino acids involved in the interaction with fructose 1,6 bisphosphate are shown as spheres (PDB 2ld). RBDpeps are shown in red. The electrostatic potential of the protein surface is shown in the bottom panel (blue, basic; red, acidic). G) As in (B) but for 14-3-3 and ERM protein families. H, I and K) Ribbon diagrams and the electrostatic potential of ERM (H), 14-3-3 (I) and Ndr (K) using homology models generated with Phyre2 (Kelley and Sternberg, 2009). J) As (B) but for NDRG protein family.

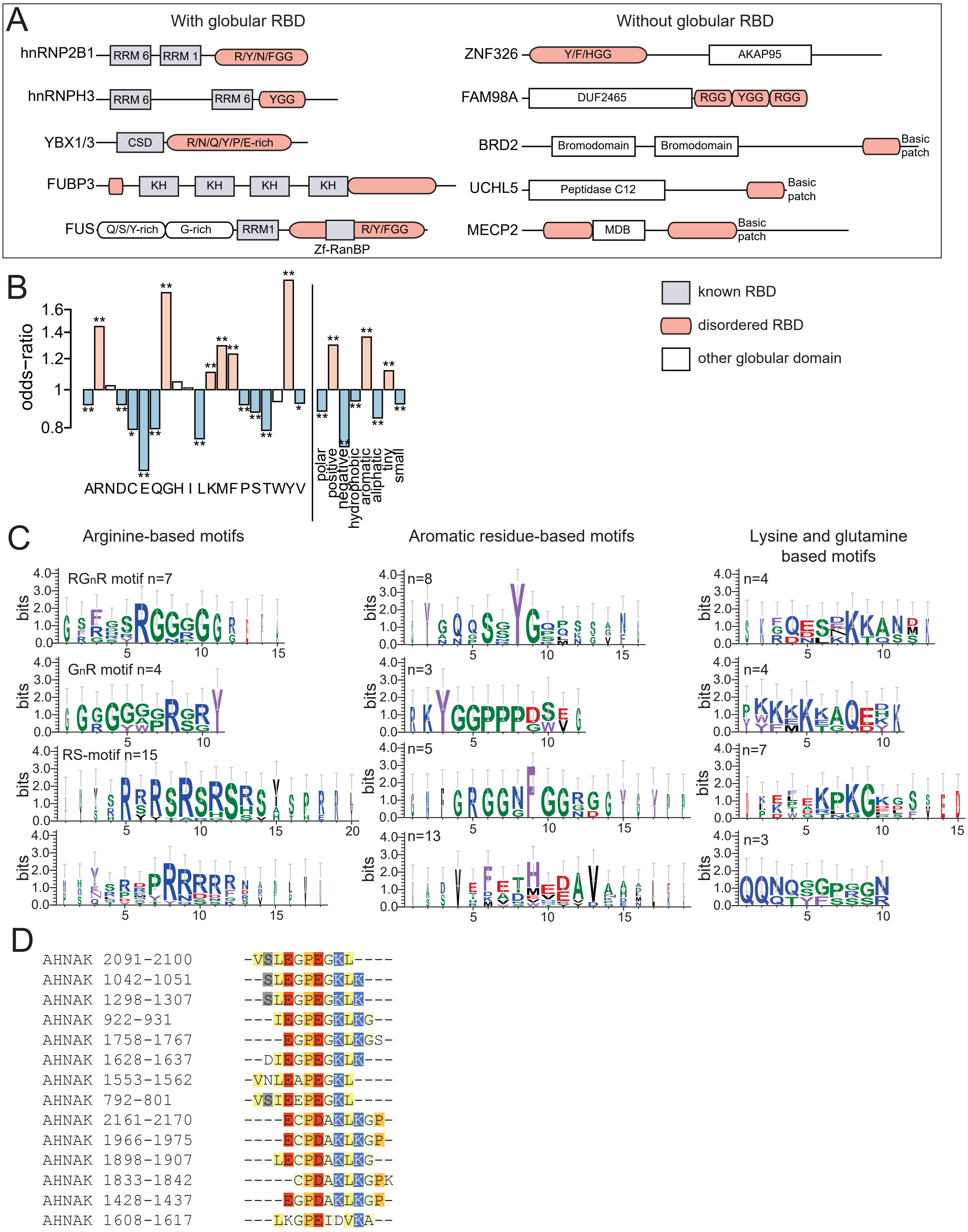

Figure S5. Disordered RNA-binding domains. Related to Figure 5

A) Schematic representation of the protein architecture of proteins harboring RNA-binding globular domains (violet) or/and disordered domains (pink). B) Amino acid enrichment within disordered RNA-bound over released proteolytic fragments mapping to disordered domains; *, 10% FDR; **, 1% FDR. C) Sequence logos extracted from aligned disordered motifs for R-based motifs, aromatic residue-based motifs and K/Q-based motifs. D) Complex pattern (VSLEGPEGKLKGP) found in multiple RBDpeps across AHNAK protein.

Pfam.name	p.value	odds.ratio	p.adj	boundPep	releasedPep
RRM_1	1.52E-82	5.953171222	1.69E-79	310	252
Pfam-B_2662	3.76E-30	4.490952613	2.10E-27	134	127
RRM_6	3.01E-20	5.749713541	1.12E-17	70	50
Pfam-B_1366	8.51E-20	6.740684806	2.37E-17	61	37
Pfam-B_4694	5.77E-13	3.724269703	1.08E-10	64	70
Pfam-B_14250	5.81E-13	9.182760264	1.08E-10	32	14
Pfam-B_7552	3.89E-11	5.513247616	6.20E-09	37	27
Pfam-B_11139	4.95E-11	16	6.91E-09	19	3
Pfam-B_6593	1.86E-10	16	2.31E-08	14	0
Pfam-B_2256	3.86E-10	3.80115872	4.31E-08	48	51
Pfam-B_2745	1.90E-09	7.990690535	1.93E-07	24	12
Pfam-B_659	2.13E-09	3.16435457	1.98E-07	54	69
DEAD	7.61E-09	0.203219496	6.53E-07	9	170
Pfam-B_12180	3.05E-08	6.556799279	2.27E-06	23	14
Pfam-B_15812	3.05E-08	6.556799279	2.27E-06	23	14
Pfam-B_1591	3.93E-08	5.140209469	2.74E-06	27	21
Pfam-B_19749	4.87E-08	16	3.20E-06	12	1
Pfam-B_19654	3.26E-07	6.877186121	2.02E-05	19	11
CSD	1.57E-06	6.759939713	9.20E-05	17	10
Pfam-B_1402	4.61E-06	16	0.000244857	9	1
ERM	4.61E-06	16	0.000244857	9	1
Pfam-B_6773	5.00E-06	16	0.000253519	10	2
Pfam-B_7751	1.02E-05	9.516635177	0.000495781	12	5
HMG_box_2	1.38E-05	16	0.000617555	7	0
Ribosomal_S19e	1.38E-05	16	0.000617555	7	0
Pfam-B_10135	1.46E-05	4.445449893	0.00062774	19	17
K167R	3.35E-05	0.0625	0.001382788	0	48
zf-CCHC	3.61E-05	8.717395407	0.001439234	11	5
Pfam-B_17918	3.80E-05	3.076407469	0.001463008	27	35
Pfam-B_2594	5.07E-05	9.900453408	0.001885413	10	4
Helicase_C	5.53E-05	0.116237005	0.001990639	2	67
zf-RNPHF	6.80E-05	11.86996167	0.002372407	9	3
Pfam-B_19575	0.000183073	3.312635041	0.006191206	20	24
HSP70	0.000271583	6.598431054	0.008914314	10	6
Pfam-B_5861	0.000337941	13.8327341	0.009014141	7	2
Pfam-B_16169	0.000337941	13.8327341	0.009014141	7	2
Pfam-B_18189	0.000339242	16	0.009014141	5	0
FKBP_C	0.000339242	16	0.009014141	5	0
Nebulin	0.000339242	16	0.009014141	5	0
PDZ	0.000339242	16	0.009014141	5	0
Linker_histone	0.000339242	16	0.009014141	5	0
Pfam-B_2659	0.000339242	16	0.009014141	5	0

Pfam-B_2097	0.000416153	3.965246813	0.010800626	14	14
WD40	0.000535201	0.188725376	0.013574652	3	62
HMG_box	0.000928926	9.222087255	0.023037374	7	3
Pfam-B_14494	0.001004204	0.093077819	0.024362871	1	42
Pfam-B_24	0.001328519	0.0625	0.030257706	0	32
Pfam-B_1911	0.001313311	11.84630165	0.030257706	6	2
HSP90	0.001313311	11.84630165	0.030257706	6	2
Pfam-B_3213	0.001708963	0.20917825	0.031265611	3	56
Tubulin-binding	0.00169282	16	0.031265611	5	1
Aldedh	0.001678458	16	0.031265611	4	0
SRPRB	0.001678458	16	0.031265611	4	0
Pfam-B_3205	0.001678458	16	0.031265611	4	0
Pfam-B_7330	0.001678458	16	0.031265611	4	0
TMA7	0.001678458	16	0.031265611	4	0
Pfam-B_1973	0.001678458	16	0.031265611	4	0
Pfam-B_14365	0.001678458	16	0.031265611	4	0
ACBP	0.001678458	16	0.031265611	4	0
Pfam-B_1644	0.001678458	16	0.031265611	4	0
Glycolytic	0.001678458	16	0.031265611	4	0
Pfam-B_2863	0.002108423	0.0625	0.037951613	0	30
Pfam-B_5724	0.002350131	0.10295157	0.041630888	1	38
Pfam-B_743	0.002585364	5.271311938	0.045082282	8	6
Pfam-B_741	0.00279851	4.449440301	0.048048267	9	8
Utp14	0.003240473	0.0625	0.053568835	0	27
Pfam-B_3767	0.003240473	0.0625	0.053568835	0	27
Cpn60_TCP1	0.003264051	7.899135924	0.053568835	6	3
Ribosomal_L14	0.004933175	9.868100083	0.079788745	5	2
Pfam-B_12462	0.005141681	0.0625	0.081973092	0	25
Pfam-B_17350	0.005321264	0.0625	0.083641283	0	26
Pfam-B_9281	0.008164941	0.0625	0.090807969	0	23
KH_1	0.007561801	1.559757252	0.090807969	57	146
LSM	0.00719125	3.558467397	0.090807969	9	10
Ribosomal_L7Ae	0.00719125	3.558467397	0.090807969	9	10
Pfam-B_14992	0.006766738	5.923626238	0.090807969	6	4
Thioredoxin	0.006766738	5.923626238	0.090807969	6	4
Pfam-B_3064	0.006766738	5.923626238	0.090807969	6	4
zf-RanBP	0.006766738	5.923626238	0.090807969	6	4
HnRNP_M	0.007035324	15.77814588	0.090807969	4	1
14-3-3	0.008299653	16	0.090807969	3	0
FTHFS	0.008299653	16	0.090807969	3	0
Pfam-B_3286	0.008299653	16	0.090807969	3	0
Pfam-B_7699	0.008299653	16	0.090807969	3	0
GAS2	0.008299653	16	0.090807969	3	0

WHEP-TRS	0.008299653	16	0.090807969	3	0
Armet	0.008299653	16	0.090807969	3	0
Peptidase_M20	0.008299653	16	0.090807969	3	0
Calponin	0.008299653	16	0.090807969	3	0
Med26	0.008299653	16	0.090807969	3	0
Ndr	0.008299653	16	0.090807969	3	0
Caldesmon	0.008299653	16	0.090807969	3	0
HTH_3	0.008299653	16	0.090807969	3	0
Ldh_1_C	0.008299653	16	0.090807969	3	0
Ldh_1_N	0.008299653	16	0.090807969	3	0
Pfam-B_1356	0.008299653	16	0.090807969	3	0
Tex_N	0.008299653	16	0.090807969	3	0
Pfam-B_6296	0.008299653	16	0.090807969	3	0
PCNP	0.008299653	16	0.090807969	3	0
Pfam-B_17673	0.008299653	16	0.090807969	3	0
Pfam-B_2728	0.008299653	16	0.090807969	3	0
Pfam-B_4483	0.008299653	16	0.090807969	3	0
Brix	0.008464474	0.0625	0.091712167	0	24

Table S2. Related to Figure 2, 3 and 4 and Table S1 and S3.
RBDs enriched in RBDmap LysC and ArgC experiments.

Gene name	Full protein name	Substrate	Class
HIBADH	3-hydroxyisobutyrate dehydrogenase, mitochondrial	NAD/NADH	di-nulceotide
PHGDH	D-3-phosphoglycerate dehydrogenase	NAD/NADH	di-nulcleotide
HADH	Trifunctional enzyme subunit alpha, mitochondrial	NAD/NADH	di-nucleotide
IDH2	Isocitrate dehydrogenase [NADP], mitochondrial	NADP/NADPH	di-nucleotide
NME1	Nucleoside diphosphate kinase A	ATP/ADP	mono-nucleotide
ADK	Adenosine kinase	ATP + adenosine > ADP + AMP	mon-nucleotide
MDH1	Malate dehydrogenase, cytoplasmic	NAD/NADH	di-nucleotide
MDH2	Malate dehydrogenase, mitochondrial	NAD/NADH	di-nucleotide
LDHB	L-lactate dehydrogenase B chain	NAD/NADH	di-nucleotide
ALDH18A1	Delta-1-pyrroline-5-carboxylate synthase	ATP/ADP	mono-nucleotide
ALDH6A1	Methylmalonate-semialdehyde dehydrogenase [acylating], mitochondrial	NAD/NADH	di-nucleotide
ALDH7A1	Alpha-aminoadipic semialdehyde dehydrogenase	NAD/NAHD; NADP/NADPH	di-nucleotide

Table S3. Related to Figure 4 and S4 and Table S2.
List of metabolic enzymes binding mono-nucleotides or di-nucleotides characterized by RBDmap.

PDB id	resolution	LysC data set	ArgC data set
1a9n	2.38	TRUE	TRUE
1aud	NMR		TRUE
1dz5	NMR		TRUE
1e8o	3.2	TRUE	TRUE
1fje	NMR	TRUE	TRUE
1fxl	1.8	TRUE	TRUE
1g2e	2.3	TRUE	TRUE
1k1g	NMR	TRUE	TRUE
1m8y	2.6	TRUE	TRUE
1rgo	NMR	TRUE	
1rkj	NMR	TRUE	TRUE
2adc	NMR	TRUE	TRUE
2fy1	NMR	TRUE	TRUE
2gxb	2.25	TRUE	TRUE
2hyi	2.3	TRUE	TRUE
2i2y	NMR	TRUE	TRUE
2j0q	3.2	TRUE	TRUE
2j0s	2.21	TRUE	TRUE
2kg1	NMR	TRUE	TRUE
2kxn	NMR	TRUE	TRUE
2l3j	NMR	TRUE	
2leb	NMR	TRUE	TRUE
2lec	NMR	TRUE	TRUE
2m8d	NMR	TRUE	TRUE
2py9	2.56	TRUE	TRUE
2rs2	NMR	TRUE	
2vod	2.1	TRUE	TRUE
2xb2	3.4	TRUE	TRUE
2xzm	3.93	TRUE	TRUE
2xzn	3.93	TRUE	TRUE
2xzo	2.4	TRUE	TRUE
2y9a	3.6	TRUE	TRUE
2y9b	3.6	TRUE	
2y9c	3.6	TRUE	TRUE
2y9d	3.6	TRUE	
2yh1	NMR	TRUE	TRUE
3a6p	2.92	TRUE	
3adl	2.2	TRUE	
3d2s	1.7	TRUE	TRUE
3ex7	2.3	TRUE	TRUE
3g9y	1.4	TRUE	TRUE
3nnc	2.2	TRUE	TRUE

3o2z	4	TRUE	TRUE
3o30	4	TRUE	TRUE
3o58	4	TRUE	TRUE
3o5h	4	TRUE	TRUE
3q0q	2	TRUE	TRUE
3q0r	2	TRUE	TRUE
3q0s	2	TRUE	TRUE
3q2t	3.06		TRUE
3rc8	2.9	TRUE	TRUE
3rw6	2.3	TRUE	TRUE
3siv	3.3	TRUE	TRUE
3snp	2.8	TRUE	
3ts2	2.01	TRUE	
3vyx	2.29	TRUE	TRUE
4b3g	2.85	TRUE	
4b8t	NMR	TRUE	TRUE
4boc	2.65	TRUE	TRUE
4bpe	3.7	TRUE	TRUE
4bpn	3.703	TRUE	TRUE
4bpo	3.7	TRUE	TRUE
4bpp	3.7	TRUE	TRUE
4ed5	2	TRUE	TRUE
4f02	2	TRUE	TRUE
4f3t	2.25	TRUE	TRUE
4krf	2.1	TRUE	

Table S5. Related to Figure 2 and 3.
List of PDB protein-RNA structures used for RBDmap validation.

ADDITIONAL FIGURE LEGENDS

Table S1. Related to Figure 1 and Figure S1.
List of RBDs and their respective peptides, identified by RBDmap.

Table S4. Related to Figure 6.
Mendelian mutations occurring within the RNA-bound fragments of RBPs and their associated diseases.

SUPPLEMENTAL EXPERIMENTAL PROCEDURES

Considerations regarding the design of RBDmap

RBDmap was designed to offer the following advances over existing methods: 1) identification of the domains of RBPs engaged with RNA in living cells, offering high-resolution RBD maps. 2) Characterization of hundreds of RBPs on a proteome-wide scale, providing the capacity for RBD "discovery" from both well-established RBPs and proteins previously unrelated to RNA. RBDmap scores endogenous protein-RNA interactions in a physiological context, since native protein-RNA pairs are covalently linked upon irradiation of cell monolayers. Note that UV crosslinking can only occur between nucleotides and amino acids in direct contact. In contrast to chemical crosslinking, UV crosslinking does not promote detectable protein-protein crosslinks (Figure S1A, Figure S4A) (Castello et al., 2013b; Pashev et al., 1991; Strein et al., 2014). 3) Protein-RNA co-structures greatly contributed to understanding protein-RNA interactions mediated by globular protein domains. Conversely, disordered domains represent a challenge for crystallization approaches. Because RBDmap can define RBDs within both globular and disordered regions, it complements structural studies. Moreover, RBDmap can be used to instruct CLIP-seq approaches by providing the RNA-binding profiles for many RBPs of interest. 4) RBDmap is here applied to steady state cell cultures, but it can be used to study in a system-wide manner the plasticity of RBDs in response to physiological alterations. 5) RBDmap further validates hundreds of novel RBPs discovered by human RNA interactome studies (Figure 1G) (Baltz et al., 2012; Castello et al., 2012) and assigns them a RNA-protein interface. It is important to highlight that the buffers used here include high salt (500 mM LiCl) and chaotropic detergents (0.5% LiDS) that efficiently remove non-covalent binders from purified RNA (Baltz et al., 2012; Castello et al., 2012; Castello et al., 2013b), as illustrated by the low protein content present in non-irradiated samples . RBDmap applies protease digestion to identify RBDs. This generates peptides of ~17 amino acids (Figure 1A), disrupting protein-protein interactions that might have withstood the stringent washing conditions.

Note that RBDmap does not cover all the proteins identified by RNA interactome capture (Figure 1G). Although experimentally related, RNA interactome capture and RBDmap differ in key aspects that may affect peptide identification by MS. Compared to RNA interactome capture, RBDmap includes a protease (LysC or ArgC) treatment prior to a second oligo(dT) purification step, as described above (Figure 1A). These additional steps reduce sample complexity and background level, facilitating the identification of additional peptides (Figure 1H). On the other hand, RBDmap may fail to assign RNA-binding sites to a number of proteins detected by RNA interactome capture for the following reasons: 1) LysC/ArgC treatment can impair peptide identification when the resulting RNA-bound peptide is identical to the tryptic peptide and no "neighboring" MS-detectable peptide can be released after trypsin treatment. Due to the frequent occurrence of arginines and lysines in RBPs, these cases may not be infrequent. 2) The two-round purification workflow of RBDmap causes increased material loss compared to RNA interactome capture and, indeed, we find that RNA recovery is reduced to about 60%. Therefore, the reduction in background described above is also accompanied with a decrease in signal. 3) We apply highly stringent statistical criteria to report a peptide as an RBDpep. The coverage of the HeLa RNA interactome would be much higher if "CandidateRBDpeps" [10% false discovery rate (FDR) instead of 1% FDR] would also be considered. Taking this set of peptides into account, RBDmap would cover most of the RBPs reported in the HeLa RNA interactome. However, to minimize the incidence of wrongly assigned RBDs (false positives), we opted to apply highly stringent 1% FDR cut-off. Since "candidateRBDpeps" could provide valuable information, this dataset is accessible in Table S1 and online (http://www-huber.embl.de/users/befische/RBDmap).

Selection of the first protease for RBDmap

An *in silico* digest of all protein sequences of the HeLa mRNA interactome (Castello et al., 2012) provided a set of theoretical proteolytic fragments for each of the eleven proteases commonly used in proteomics. Tryptic peptides identified in the HeLa mRNA interactome were mapped onto the proteolytic fragments predicted for each protease. We set a theoretical RNA-binding site in the center of the protein and monitored the number of cases where the protease fragment covers the theoretical binding site. The

RBDmap resolution for each protease was determined as the number of proteins for which a given protease can narrow down the RNA-binding site to less than 20% of the actual protein length. LysC and ArgC were identified as the proteases that theoretically would perform better in a higher number of proteins of the HeLa RNA interactome. However, other proteases may outcompete LysC and ArgC in a case-dependent manner.

The RBDmap protocol

HeLa cells were grown overnight on six 500cm^2 dishes in DMEM medium supplemented with 10% fetal calf serum. Three of the plates were incubated overnight with 100 µM 4-thiouridine (4SU) for PAR-CL. After PBS wash, 0.15 J/cm^2 UV light at 254nm (for cCL) was applied on untreated cell monolayers (3 dishes) and 365nm (for PAR-CL) on 4SU-treated cell monolayers (3 dishes), as previously described (Castello et al., 2013b). Cells were harvested and lysed in a buffer containing 20mM pH 7.5 Tris HCl, 500mM LiCl, 0.5% LiDS, 1mM EDTA and 5 mM DTT and homogenized by passing the sample through a syringe with a narrow gauge needle (0.4 mm diameter). Proteins crosslinked to poly(A)$^+$ mRNAs were captured with oligo(dT)$_{25}$ magnetic beads (NE Biolabs). Subsequently, oligo(dT)$_{25}$ beads were washed with buffers containing decreasing concentrations of LiCl and LiDS, as previously described (Castello et al., 2013b). RNAs and crosslinked proteins were eluted with 20mM Tris HCl, pH 7.5 at 55°C for 3 min. 70 µl were taken for RNA and protein quality controls as previously described (Castello et al., 2013b). For RNA analysis, samples were digested with proteinase K, followed by RNA isolation with RNeasy (Qiagen). The remaining sample was treated with 1µg of LysC or ArgC, and supplemented with 1 µl of RNaseOUT (Promega) and 5x of the protease buffer as described by the manufacturer. After digestion at 37°C for 8h, 70 µl were taken for RNA and protein quality controls as described (Castello et al., 2013b). 1/3 of the sample from irradiated and non-irradiated cells was taken for mass spectrometry (input) and processed as indicated below. The rest of the sample was diluted 2 ml of 5x dilution buffer (2.5 M LiCl, 100mM pH 7.5 Tris HCl, 5 mM EDTA and 25 mM DTT) and H_2O (10 ml total volume), and incubated with 2 ml of oligo(dT) beads for 1 h. After separating the beads with a magnet, the supernatant was collected and kept at 4°C (released fraction). Beads are washed once with 500mM LiCl and 0.5% LiDS containing buffer, and with buffers containing decreasing concentrations of LiCl and LiDS as previously described (Castello et al., 2013b). The RNA-bound fraction is eluted with 20mM Tris HCl, pH 7.5 for 3 min at 55°C. All input, supernatant (released) and eluates (RNA-bound) are treated with RNase T1 and RNase A (Sigma). Samples were then processed for MS as described below.

Sample preparation for MS

Samples were processed according to standard protocols (Wisniewski et al., 2009) with minor modifications. Cysteines were reduced (5 mM DTT, 56˚C, 30 min) and alkylated (10 mM Iodoacetamide, 30 min in the dark). Samples were buffer-exchanged into 50 mM triethylammoniumbicarbonate, pH 8.5, using 3 kDa centrifugal filters (Millipore) and digested with sequencing grade trypsin (Promega, enzyme-protein ratio 1:50) at 37˚C for 18 h. Resulting peptides were desalted and labelled using stable isotope reductive methylation (Boersema et al., 2009) on StageTips (Rappsilber et al., 2007). Labels were swapped between replicates. Labeled samples were combined and fractionated into 12 fractions on an 3100 OFFGEL Fractionator (Agilent) using Immobiline DryStrips (pH 3–10 NL, 13 cm; GE Healthcare) according to the manufacturer's protocol. Isoelectric focusing was carried out at a constant current of 50 mA allowing a maximum voltage of 8000 V. When 20 kVh were reached the fractionation was stopped, fractions were collected and desalted using StageTips. Samples were dried in a vacuum concentrator and reconstituted in MS loading buffer (5% DMSO 1% formic acid).

LC-MS/MS

Samples were analyzed on a LTQ-Orbitrap Velos Pro mass spectrometer (Thermo Scientific) coupled to a nanoAcquity UPLC system (Waters). Peptides were loaded onto a trapping column (nanoAcquity Symmetry C_{18}, 5 µm, 180 µm × 20 mm) at a flow rate of 15 µl/min with solvent A (0.1% formic acid). Peptides were separated over an analytical column (nanoAcquity BEH C18, 1.7 µm, 75 µm × 200 mm) using a 110 min linear gradient from 7-40% solvent B (acetonitrile, 0.1% formic acid) at a constant flow rate of 0.3 µl/min. Peptides were introduced into the mass spectrometer using a Pico-Tip Emitter (360 µm outer diameter × 20 µm inner diameter, 10 µm tip, New Objective). MS survey scans were acquired from 300–1700 *m/z* at a nominal resolution of 30000. The 15 most abundant peptides were isolated within a 2 Da window and subjected to MS/MS sequencing using collision-induced dissociation in the ion trap (activation time 10 msec, normalized collision energy 40%). Only 2+/3+ charged ions were included for analysis. Precursors were dynamically excluded for 30 sec (exclusion list size was set to 500).

Peptide identification and quantification

Raw data were processed using MaxQuant (version 1.3.0.5) (Cox and Mann, 2008). MS/MS spectra were searched against the human UniProt database (version 12_2013) concatenated to a database containing protein sequences of common contaminants. Enzyme specificity was set to trypsin/P, allowing a maximum of two missed cleavages. Cysteine carbamidomethylation was set as fixed modification, and methionine oxidation and protein N-terminal acetylation were used as variable modifications. The minimal peptide length was set to six amino acids. The mass tolerances were set to 20 ppm for the first search, 6 ppm for the main search and 0.5 Da for product ion masses. False discovery rates for peptide and protein identification were set to 1%. Match between runs (time window 2 min) and re-quantify options were enabled.

Statistical Analysis

To identify the “input” peptides, the intensity of peptides in crosslinked was compared to non-crosslinked samples after oligo(dT) capture. To test whether the log2-intensity ratio of each peptide in three replicated experiments is different from zero, p-values were computed by a moderated t-test implemented in the R/Bioconductor package limma (Smyth, 2004). p-values were corrected for multiple testing by controlling the false discovery rate with the method of Benjamini-Hochberg. A peptide set with a false discovery rate (FDR) of 1% was used for further analysis.

To identify RNA-binding sites, the log2 intensity ratio in the RNA-bound to the released fraction was considered. The distribution of the log2-ratios is bi-modal, representing the released and RNA-bound peptides. The log2-ratios are normalized to the location of the left mode using a robust estimate. Log2-ratios of each peptide in three replicate experiments were tested against zero by a moderated t-test from the R/Bioconductor package limma (Smyth, 2004), and p-values were corrected for multiple testing by the method of Benjamini-Hochberg. Peptides with a 1% FDR are termed ‘RBDpep’. Peptides extending this set to a 10% FDR are called ‘CandidateRBDpep’. For further analysis and to identify the protein set covered by these peptides, only peptides uniquely mapping to a gene model are considered.

Computational validation of identified binding sites by correlation with domain annotations

To validate the identified binding sites and to distinguish them from non-binding sites, all proteins with at least one RBDpep covering a classical RBD and one RBDpep mapping outside a classical RBD were considered. RBDpeps were sorted by their log2- RNA-bound/released intensity ratios. For each window of 101 peptides, comprising the RBDpep under consideration plus 50 peptides on either side of this viewpoint, the probability that the RBDpep is within a classical RBD were considered. The probability that the RBDpep is within a classical RBD is computed as the fraction of RBDpeps that cover classical RBDs over the fraction of peptides mapping outside the RBD.

RBD maps: data display and interpretation

MS-identified tryptic peptides enriched in the RNA-bound or released fractions, respectively, are mapped back to proteins and extended to the two adjacent LysC or ArgC cleavage sites to recall the original proteolytic fragment. LysC and ArgC proteolytic fragments are plotted regarding their position within the protein (x axis: N- to C-termini) and their fold change between the RNA-bound and released fractions (y axis), as exemplified in Figure 2D. 1% FDR RBDpeps and 10% FDR candidateRBDpeps are shown in red and salmon, respectively, while released fragments are shown in blue. Boxes below the plot are used to visualize the position of the protein’s domains.

Frequently, a given domain is mapped by multiple RBDpeps, reflecting the reliability of RBDmap. In some instances two proteolytic fragments overlap partially or almost completely but display different RNA-bound/released fold changes. Because we only use uniquely mapped peptides, overlapping peptides can be explained as follows: 1) The peptides are non-identical (i.e. one or two amino acids longer or shorter). This can occur when the protease encounters multiple cleavage sites adjacent to each other, allowing differential proteolysis. Since proteases require a number of amino acids on both sides of the scission site, cleavage at a given amino acid may abrogate cleavage at an adjacent site. 2) The two peptides are generated by different proteases. To facilitate the interpretation we indicate the protease from which it originates (L for LysC; A for ArgC) adjacent to the RBDpep. In the online version (http://www-huber.embl.de/users/befische/RBDmap), the identity of the protease can be seen by passing the cursor over the peptide line. In most cases, overlapping LysC and ArgC fragments exhibit comparable RNA-bound/released ratios, confirming the same RNA-binding sites within a protein with two independent proteases. As a general rule, the shorter RBDpep provides the higher resolution. However, in rare cases, a given region can be found to be RNA-bound with one protease and released with the other. This outcome implies that one of the peptides harbors the RNA-binding site, thus qualifying as RBDpep, and the other does not.

To integrate data from homologous and non-homologous proteins, we classified the proteins based on the domains identified as RBDs (e.g. FKBP protein family). We aligned the domain exhibiting RNA-binding activity (e.g. FKBP fold) from homologs and non-homologs harboring it. The relative position of each RBDpep was extracted and plotted as a "block". The number of independent peptide "blocks" accumulated at a given position reflects the prevalence of an RNA-binding site across the proteins sharing the same domain (e.g. Figure S2A). RBD classification can be visualized and browsed under "globular domains" on the website http://www-huber.embl.de/users/befische/RBDmap/.

Characterization of RBDpeps

Domain enrichment. For gene set enrichment analysis of RBDs, we used the Pfam domain annotation (Finn et al., 2014) in the Interpro database (Hunter et al., 2012; McDowall and Hunter, 2011). For each identified LysC/ArgC proteolytic fragment in the RNA-bound fraction or in the input, we scored whether it overlaps with a Pfam domain or not. Fisher's exact test was used to compute p-values for enrichment. p-values were corrected for multiple testing by the method of Benjamini-Hochberg. Pfam domains with a false discovery rate of 10% are reported.

Identification of disordered fragments. The intrinsically unstructured or disordered parts of a protein were predicted by "iupred" (Dosztanyi et al., 2005). Amino acids with an iupred score of >0.4 were considered as being present in a disordered region. A proteolytic fragment of identified peptides is regarded as disordered, if the average iupred score is larger than 0.4.

Amino acid composition. The amino acid composition of all RBDpep or released fragments is compared to the amino acid composition of all input fragments. For analysis of disordered or globular RNA-binding sites, RNA-bound or released proteolytic fragments overlapping with disordered or globular protein segments were compared to disordered or globular input fragments. Over-/underrepresentation of a given amino acid was tested by Fisher's exact test, and p-values were corrected for multiple testing by the method of Benjamini-Hochberg.

Tripeptide enrichment. p-values for motif enrichment of triplet amino acids were computed by a binomial test using the fraction of the total length of all RBDpep fragments over the total length of all fragments as the hypothesized probability of success. P-values were Benjamini-Hochberg corrected for multiple testing.

Motif alignment. To identify specific sequences that occur within disordered RNA-binding sites, the RBDmap fragments were mapped onto the proteins. The detected RNA-binding sites were dissected into half-overlapping sequences of a maximum length of 11 amino acids. The multiple sequence alignment software clustal omega (Release 1.2.0) (Sievers et al., 2011) was used for multiple sequence alignment. The cluster tree is cut at h=10. Sequences within each cluster were aligned again. Sequence logos showing the information content of each amino acid position were plotted with weblogo (Release 3.3) (Crooks et al., 2004) for each cluster. The amino acid composition of the input fragments was used as background. Prevalent amino acids in the motif logo may bind RNA or be involved in other functions such as binding regulation (e.g. PTM) or disorder promotion (e.g. G, S and P).

Posttranslational modifications. Annotations of post-translational modifications (PTMs) were downloaded from Uniprot (Release 2013_12). PTM enrichment analysis was performed as for Pfam domains (see above). The amino acid enrichment in a window of +/- 6 amino acids around the PTM was computed for RNA-bound and input fragments. Sequence logos showing the relative entropy of the amino acid compositions were plotted.

Disease-associated mutations. Sequence variants associated with diseases from OMIM (Brandt, 1993; Castello et al., 2013a) and natural sequence variants were downloaded from Uniprot (Release 2013_12). Variants overlapping with RNA-bound or released proteolytic fragments were classified into disease-associated or non-pathological. Statistical significance of enrichment of disease variants in RNA-bound fragments was assessed by Fisher's exact test.

RBP abundance and isoelectric point: the mean normalized mRNA level over 16 arrays of HeLa cells extracted from the ArrayExpress atlas (ArrayExpress accession E-MTAB-62) was used to assess the mRNA levels of proteins within the HeLa whole proteome, RNA interactome, input fraction and RBDmap dataset. This approach was also employed to infer the abundance of previously known RBPs as well as proteins harboring novel globular or disordered RBDs. The isoelectric point (Ip) implemented in the *trans* proteomic pipeline was used to analyzed the Ip distribution of these protein groups.

RBDpep conservation: RNA-bound and released LysC/ArgC fragments were aligned to the whole proteomes of *Danio rerio*, *Drosophila melanogaster*, *Caenorhabditis elegans*, and *Saccharomyces cerevisiae* (UniProt release 2015_01) using BLASTP 2.2.26. A fragment was classified as conserved, if it matches a protein with an e-value <= 1. The fraction of RNA-bound peptides beyond all conserved peptides are tested against the hypothesis that it is equal to the fraction of RNA-bound peptides beyond all fragments (RNA-bound and released) using a binomial test.

Validation of identified binding sites based on PDB structures

For computational validation of RBDmap data, 3D structures of protein-RNA complexes deposited in the PDB databank were used (downloaded October 2013). Only NMR and x-ray diffraction structures with resolutions better than 5.0 Å were considered. Protein sequences in PDB structures were first aligned with RBDmap LysC and ArgC fragments (10% FDR) matching 305 human UniProt protein sequences (version 12_2013). We used local Smith-Waterman alignment (EMBOSS water, gap open penalty 10.0, gap extend penalty 0.5, EBLOSUM90). Because reported structures may contain short deletions and to allow alignment with highly conserved protein-RNA interactions from different organisms, hits with identity larger than 70% and with gaps less than 10% of the reported aligned region were considered further. This resulted in a total of 67 structures containing protein-RNA complexes (64 and 56 structures for LysC and ArgC, respectively, see Table S5), which were used for computational validation of RBDmap data.

Protein sequences of selected 3D structures were then segmented in silico into LysC or ArgC fragments, respectively. For each fragment, the distance from the closest amino acid (β-carbon) to the RNA atoms was calculated. Fragments at distances closer than 4.3 Å to RNA were classified as proximal (49% for LysC, 57% for ArgC), all others as non-proximal. Because most of the polypeptides used in these studies represent only the RBD of the protein (e.g. RRM of PABPC1), we observe a bias towards proximal peptides. Indeed, about half of the LysC and ArgC proteolytic fragments are classified as proximal (Fig 1k and Extended Data 2). However, the overlap between proximal fragments and RBDpeps is 70% for LysC and 81% for ArgC, implying that RBDmap highly significantly enriches for peptides in close proximity with the RNA. Significance of the overlap was calculated with the hypergeometric test.

Generation of high-resolution profiles using the ratio of X-link over released peptide coverage

We collected the superset of RBDpeps and released peptides mapping to each RBD type. The position of these peptides within the domain or in upstream or downstream protein regions is mapped to a linear scale from -1 to 2, with the RBD itself spanning the range from 0 to 1 and the flanking regions from -1 to 0 and from 1 to 2, respectively. We then subtracted the MS-identified portion of RBDpeps and released fragments. Through MS-identified N-link peptide removal we can infer the X-link moiety which represents the actual RNA-binding portion, as described in Figure 1A. Domain profiles in Figure 3B-E are generated by calculating the ratio between X-link and released peptide coverage at each position of the domain. To compute the ratio, a pseudo count of 3 was added to avoid artifacts with low count numbers. For normalization, the computed ratio was divided by the ratio between the complete pool of X-link peptides and RNA-released peptides mapping to the complete set of proteins harboring the domain under study, resulting in the displayed fold change.

Secondary structure was predicted using NetSurfP (version 1.1, (Petersen et al., 2009)) for the same protein domains and mapped to the scale of -1 to 2 as above. Thereby the probabilities for alpha-helices and beta-strands were linearly approximated. The profiles display the mean of all predictions.

Establishment of stable HeLa cell lines

Chimeric cDNAs obtained by PCR from a HeLa cDNA library were used as template for PCR. Inserts were inserted into pCDNA5/FRT/TO- eGFP (Strein et al., 2014) or pCDNA5/FRT/TO-FLAG-HA. These plasmids include a glycine(G)-serine(S) (GGSGGSGG) linker between the tag and the protein of interest. Generation of the stable cell lines was performed as described in the manufacturer's protocol (Flp In TRex, Invitrogen). Protein is induced by addition of tetracycline as described elsewhere (Castello et al., 2012).

Interactome capture for eGFP-tagged proteins

1x500 cm^2 dish at 50% confluence of HeLa TRex cells expressing the different eGFP-fusion proteins were induced overnight with 1µg/ml tetracycline. Cell monolayers were irradiated with 0.15 J/cm^2, 254 nm UV light, and lysed into 500 mM and 0.5% LiDS-containing buffer as in (Castello et al., 2013b). $Poly(A)^+$ RNAs and crosslinked proteins were captured with 500 µl of $oligo(dT)_{25}$ magnetic beads. Subsequently, $oligo(dT)_{25}$ beads were washed with buffers containing decreasing concentrations of LiCl and LiDS, as previously described (Strein et al., 2014). After elution into 20mM Tris HCl, pH 7.5 at 55^oC for 3 min, eluates were concentrated in a 3KDa amicon device to 20µl final volume, cooled at 4^oC for 10 min, and loaded in a 96 well plate with transparent bottom. eGFP measurement was performed as in (Strein et al., 2014).

PNK assay

Cells expressing FLAG-HA fusion proteins were UV-crosslinked on ice (150 mJ/cm^2), lysed (500 mM NaCL, 30 mM Tris pH 7.5, 5% glycerol, 0.1% Triton X-100, 2 mM Mg_2Cl, 4 mM β-mercaptoethanol and protease inhibitors), and homogenized passing the lysate through a narrow needle (22G) followed by pulsed ultrasonication (3 × 10 s, 50% amplitude, on ice). Cleared lysates were treated with 50 U/ml DNAseI (Takara) and RNaseI for 15 min at 37 °C, and used for immunoprecipitation with FLAG M2 coupled to magnetic beads (M8823, Sigma) for 2h at 4°C. Beads were washed once with lysis buffer and five times with wash buffer (100 mM NaCL, 30 mM Tris pH 7.5, 5% glycerol, 0.1% Triton X-100, 2 mM Mg_2Cl, 4 mM β-mercaptoethanol and protease inhibitors). RNA crosslinked to the tagged RBD is identified by radiolabeling with 0.1 µCi/µl γ-32P ATP by T4 polynucleotide kinase (1U/µl) in PNK buffer (50 mM NaCL, 50 mM Tris pH 7.5, 0.5% NP-40, 10 mM Mg_2Cl and 5 mM DTT) for 15 min at 850 rpm and 37°C. Beads were washed four to six times with PNK buffer and protein-RNA complexes were eluted with a 3-fold excess of FLAG peptide. Samples were analyzed by SDS PAGE and autoradiography.

Immunoprecipitation of eGFP and YFP fusion proteins

One 10 cm dish of Tet-inducible cell lines expressing eGFP- or YFP-tagged proteins were treated with tetracycline overnight and cell monolayers were UV irradiated (150 mJ/cm^2) on ice after two PBS washes. Cells were lysed with GBP (GFP-binding protein) lysis buffer (500 mM NaCl, 20 mM pH 7.5 Tris-HCl, 2 mM Mg2Cl, 0.025% SDS, 0.1% Triton X-100 and protease inhibitors). Cell lysates were homogenized by passing them through a narrow needle (22G). Extracts were incubated with 10 µl of equilibrated GFP_trap_A (Chromotek) for 2h at 4°C. Beads were washed three times with GBP lysis buffer and 3 times with GBP wash buffer (150 mM NaCl, 20 mM pH 7.5 Tris-HCl, 2 mM Mg2Cl, 0.01% Triton X-100 and protease inhibitors). Proteins were eluted with loading buffer for 5 min at 95°C. Eluates were analysed by SDS PAGE followed by silver staining (see below).

Silver staining analysis

Proteins co-isolated by oligo(dT) pull down or in immunoprecipitation experiments were analyzed by silver staining, according to standard protocols (Castello et al., 2012).

Data dissemination

The mass spectrometry proteomics data have been deposited with the ProteomeXchange Consortium (http://www.proteomexchange.org) via the PRIDE partner repository (Vizcaino et al., 2013) with the dataset identifier PXD000883".

The details:

ProteomeXchange title: RBDmap

ProteomeXchange accession: PXD000883

Reviewer account:

- Username: reviewer46276@ebi.ac.uk
- Password: xg0ioRX5
- To access the data please visit: http://tinyurl.com/pu7yodo

RBDmap analyses and protein profiles can be visualised at:
http://www-huber.embl.de/users/befische/RBDmap

SUPPLEMENTAL REFERENCES

Arif, A., Jia, J., Mukhopadhyay, R., Willard, B., Kinter, M., and Fox, P.L. (2009). Two-site phosphorylation of EPRS coordinates multimodal regulation of noncanonical translational control activity. Mol Cell *35*, 164-180.

Boersema, P.J., Raijmakers, R., Lemeer, S., Mohammed, S., and Heck, A.J. (2009). Multiplex peptide stable isotope dimethyl labeling for quantitative proteomics. Nat Protoc 4, 484-494.

Brandt, K.A. (1993). The GDB Human Genome Data Base: a source of integrated genetic mapping and disease data. Bull Med Libr Assoc 81, 285-292.

Cox, J., and Mann, M. (2008). MaxQuant enables high peptide identification rates, individualized p.p.b.-range mass accuracies and proteome-wide protein quantification. Nat Biotechnol 26, 1367-1372.

Crooks, G.E., Hon, G., Chandonia, J.M., and Brenner, S.E. (2004). WebLogo: a sequence logo generator. Genome Res 14, 1188-1190.

Chakrabarti, S., Jayachandran, U., Bonneau, F., Fiorini, F., Basquin, C., Domcke, S., Le Hir, H., and Conti, E. (2011). Molecular mechanisms for the RNA-dependent ATPase activity of Upf1 and its regulation by Upf2. Mol Cell 41, 693-703.

Dosztanyi, Z., Csizmok, V., Tompa, P., and Simon, I. (2005). IUPred: web server for the prediction of intrinsically unstructured regions of proteins based on estimated energy content. Bioinformatics 21, 3433-3434.

Finn, R.D., Bateman, A., Clements, J., Coggill, P., Eberhardt, R.Y., Eddy, S.R., Heger, A., Hetherington, K., Holm, L., Mistry, J., et al. (2014). Pfam: the protein families database. Nucleic Acids Res 42, D222-230.

Hunter, S., Jones, P., Mitchell, A., Apweiler, R., Attwood, T.K., Bateman, A., Bernard, T., Binns, D., Bork, P., Burge, S., et al. (2012). InterPro in 2011: new developments in the family and domain prediction database. Nucleic Acids Res 40, D306-312.

Kelley, L.A., and Sternberg, M.J. (2009). Protein structure prediction on the Web: a case study using the Phyre server. Nat Protoc 4, 363-371.

McDowall, J., and Hunter, S. (2011). InterPro protein classification. Methods Mol Biol 694, 37-47.

Mukhopadhyay, R., Ray, P.S., Arif, A., Brady, A.K., Kinter, M., and Fox, P.L. (2008). DAPK-ZIPK-L13a axis constitutes a negative-feedback module regulating inflammatory gene expression. Mol Cell 32, 371-382.

Petersen, B., Petersen, T.N., Andersen, P., Nielsen, M., and Lundegaard, C. (2009). A generic method for assignment of reliability scores applied to solvent accessibility predictions. BMC Struct Biol 9, 51.

Rappsilber, J., Mann, M., and Ishihama, Y. (2007). Protocol for micro-purification, enrichment, pre-fractionation and storage of peptides for proteomics using StageTips. Nat Protoc 2, 1896-1906.

Sievers, F., Wilm, A., Dineen, D., Gibson, T.J., Karplus, K., Li, W., Lopez, R., McWilliam, H., Remmert, M., Soding, J., et al. (2011). Fast, scalable generation of high-quality protein multiple sequence alignments using Clustal Omega. Mol Syst Biol 7, 539.

Smyth, G.K. (2004). Linear models and empirical bayes methods for assessing differential expression in microarray experiments. Stat Appl Genet Mol Biol 3, Article3.

Vizcaino, J.A., Cote, R.G., Csordas, A., Dianes, J.A., Fabregat, A., Foster, J.M., Griss, J., Alpi, E., Birim, M., Contell, J., et al. (2013). The Proteomics Identifications (PRIDE) database and associated tools: status in 2013. Nucleic Acids Research 41, D1063-D1069.

Wisniewski, J.R., Zougman, A., Nagaraj, N., and Mann, M. (2009). Universal sample preparation method for proteome analysis. Nat Methods 6, 359-362.
